# Comparative analyses of *Legionella* species identifies genetic features of strains causing Legionnaires’ disease

**DOI:** 10.1186/s13059-014-0505-0

**Published:** 2014-11-03

**Authors:** Laura Gomez-Valero, Christophe Rusniok, Monica Rolando, Mario Neou, Delphine Dervins-Ravault, Jasmin Demirtas, Zoe Rouy, Robert J Moore, Honglei Chen, Nicola K Petty, Sophie Jarraud, Jerome Etienne, Michael Steinert, Klaus Heuner, Simonetta Gribaldo, Claudine Médigue, Gernot Glöckner, Elizabeth L Hartland, Carmen Buchrieser

**Affiliations:** Institut Pasteur, Biologie des Bactéries Intracellulaires, 28, rue du Dr. Roux, 75724 Paris, Cedex 15, France; CNRS UMR 3525, 75724 Paris, France; The ithree institute, University of Technology Sydney, Sydney, NSW 2007 Australia; CIRI, International Center for Infectiology Research, Inserm, U1111, CNRS, UMR5308, Université Lyon 1, École Normale Supérieure de Lyon, Lyon, F-69008 France; National Reference Center of Legionella, Hospices Civils de Lyon, 69677 Paris, France; Institut für Mikrobiologie, Technische Universität Braunschweig, Braunschweig, 38106 Germany; Cellular Interactions of Bacterial Pathogens, Centre for Biological Threats and Pathogens (ZBS2), Robert Koch-Institute, Nordufer 20, 13353 Berlin, Germany; Institut Pasteur, Unité Biologie Moléculaire du Gene chez les Extrêmophiles, Paris, 75015 France; CEA/DSV/IG/Genoscope & CNRS UMR8030, Laboratoire d’Analyses Bioinformatiques en Génomique et Métabolisme, Evry, 91000 France; Australian Animal Health Laboratories, CSIRO, Geelong, VIC 3220 Australia; Genomics, Institute for Biochemistry I, Medical Faculty, University of Cologne, D50931 Germany and Leibniz-Institute of Freshwater Ecology and Inland Fisheries, Berlin, 10997 Germany; Department of Microbiology and Immunology, University of Melbourne at the Peter Doherty Institute for Infection and Immunity, Melbourne, VIC 3010 Australia

**Keywords:** *Legionella*, Genome, Legionnaires’ disease, Mobilome, Eukaryotic like proteins

## Abstract

**Background:**

The genus *Legionella* comprises over 60 species. However, *L. pneumophila* and *L. longbeachae* alone cause over 95% of Legionnaires’ disease. To identify the genetic bases underlying the different capacities to cause disease we sequenced and compared the genomes of *L. micdadei*, *L. hackeliae* and *L. fallonii* (LLAP10), which are all rarely isolated from humans.

**Results:**

We show that these *Legionella* species possess different virulence capacities in amoeba and macrophages, correlating with their occurrence in humans. Our comparative analysis of 11 *Legionella* genomes belonging to five species reveals highly heterogeneous genome content with over 60% representing species-specific genes; these comprise a complete prophage in *L. micdadei*, the first ever identified in a *Legionella* genome. Mobile elements are abundant in *Legionella* genomes; many encode type IV secretion systems for conjugative transfer, pointing to their importance for adaptation of the genus. The Dot/Icm secretion system is conserved, although the core set of substrates is small, as only 24 out of over 300 described Dot/Icm effector genes are present in all *Legionella* species. We also identified new eukaryotic motifs including thaumatin, synaptobrevin or clathrin/coatomer adaptine like domains.

**Conclusions:**

*Legionella* genomes are highly dynamic due to a large mobilome mainly comprising type IV secretion systems, while a minority of core substrates is shared among the diverse species. Eukaryotic like proteins and motifs remain a hallmark of the genus *Legionella*. Key factors such as proteins involved in oxygen binding, iron storage, host membrane transport and certain Dot/Icm substrates are specific features of disease-related strains.

**Electronic supplementary material:**

The online version of this article (doi:10.1186/s13059-014-0505-0) contains supplementary material, which is available to authorized users.

## Background

Among the many pathogens provoking severe pneumonia, the Gram-negative bacteria *Legionella pneumophila* and *Legionella longbeachae* are responsible for Legionnaires’ disease, a severe pneumonia that can be deadly if not treated promptly [1]. Although several of the more than 60 species described in the genus *Legionella* may cause disease, *L. pneumophila* is the major agent, responsible for nearly 90% of all cases worldwide. *L. longbeachae* comes second, causing around 2 to 7% of cases with the exception of Australia and New Zealand, where it is associated with 30% of Legionnaires’ disease cases [2]. *Legionella micdadei*, *Legionella bozemanii*, *Legionella dumoffii*, *Legionella anisa*, *Legionella wadsworthii* and *Legionella feelei* are rarely found in humans and the remaining *Legionella* species have never or only once been isolated from humans [2]. This highly significant difference in disease incidence among *Legionella* species may be due to different environmental distributions and/or to different virulence potential for humans. Few studies have analyzed the environmental distribution of *Legionella*, although one survey in France showed that *L. pneumophila*, which had a prevalence of 95.4% in clinical isolates, was found in only 28.2% of the environmental samples tested, whereas *L. anisa* was isolated in 13.8% of the environmental samples but found only once (0.8%) in a clinical isolate [3]. Similarly, a more recent report from Denmark showed that only 4.5% of clinical cases were due to non-*L. pneumophila* strains and reported a strong discrepancy in the occurrence of different *Legionella* species in clinical and environmental isolates [4]. For example, *L. anisa* was highly abundant in the environment, but never found in clinical isolates. In contrast, *L. bozemanni*, *L. longbeachae* and *L. micdadei* were identified in clinical samples but never or rarely in environmental samples [4]. Furthermore, different *Legionella* species also seem to have a different host range and different capacities to infect human cells [5,6]. Taken together, independently of the environmental distribution, different *Legionella* species seem also to possess different abilities to infect eukaryotic cells and to cause disease in humans.

After publication of the *L. pneumophila* genome sequence in 2004 [7,8] and that of *L. longbeachae* in 2010 [9,10] several additional *L. pneumophila* strains have been sequenced [11-14] as well as a few draft genome sequences of other species. However, apart from *Legionella oakridgensis* [15] none has been analyzed in detail. Thus the vast majority of comprehensively analyzed genome sequences are from the major human pathogens *L. pneumophila* (eight genomes) and *L. longbeachae* (two genomes). In order to deepen our insight into species never or rarely found in human disease, we completely sequenced and analyzed the genomes of three *Legionella* species, *L. micdadei*, *Legionella hackeliae* and *Legionella fallonii* (LLAP10), selected based on their different epidemiological characteristics compared with *L. pneumophila* and *L. longbeachae. L. micdadei* is found in less than 1% of community-acquired pneumonia [2], *L. hackeliae* has been isolated from humans only once [16], and *L. fallonii* has never been reported to cause disease. *L. fallonii* was originally designated LLAP10 for ‘legionella-like amoebal pathogen 10’ [17], a term coined by Rowbotham for bacteria that caused legionella-like infections in amoebae, but could not be grown on agar media*.*

Here we analyze and compare the *L. micdadei*, *L. hackeliae* and *L. fallonii* genomes and compare them with seven previously completely sequenced *L. pneumophila* (Paris, Philadelphia, Lens, Corby, Alcoy, Lorraine and HL06041035) [7,8,11,14] and one *L. longbeachae* NSW150 genome sequence [9]. We confirm that the presence of 'eukaryotic-like proteins' (ELPs) is indeed a specific feature of the genus *Legionella* and extend the knowledge of these proteins further by identifying additional eukaryotic motifs. Analyses of the virulence of the different *Legionella* species in protozoan and human cells correlated with the genetic content and allowed us to identify specific features of human pathogenic *Legionella* and to define a core set of 24 type IV secretion system (T4SS) effectors present in the *Legionella* species examined to date.

## Results and discussion

### *L. micdadei*, *L. hackeliae* and *L. fallonii* show different virulence in amoeba or macrophages

**Figure 1:**
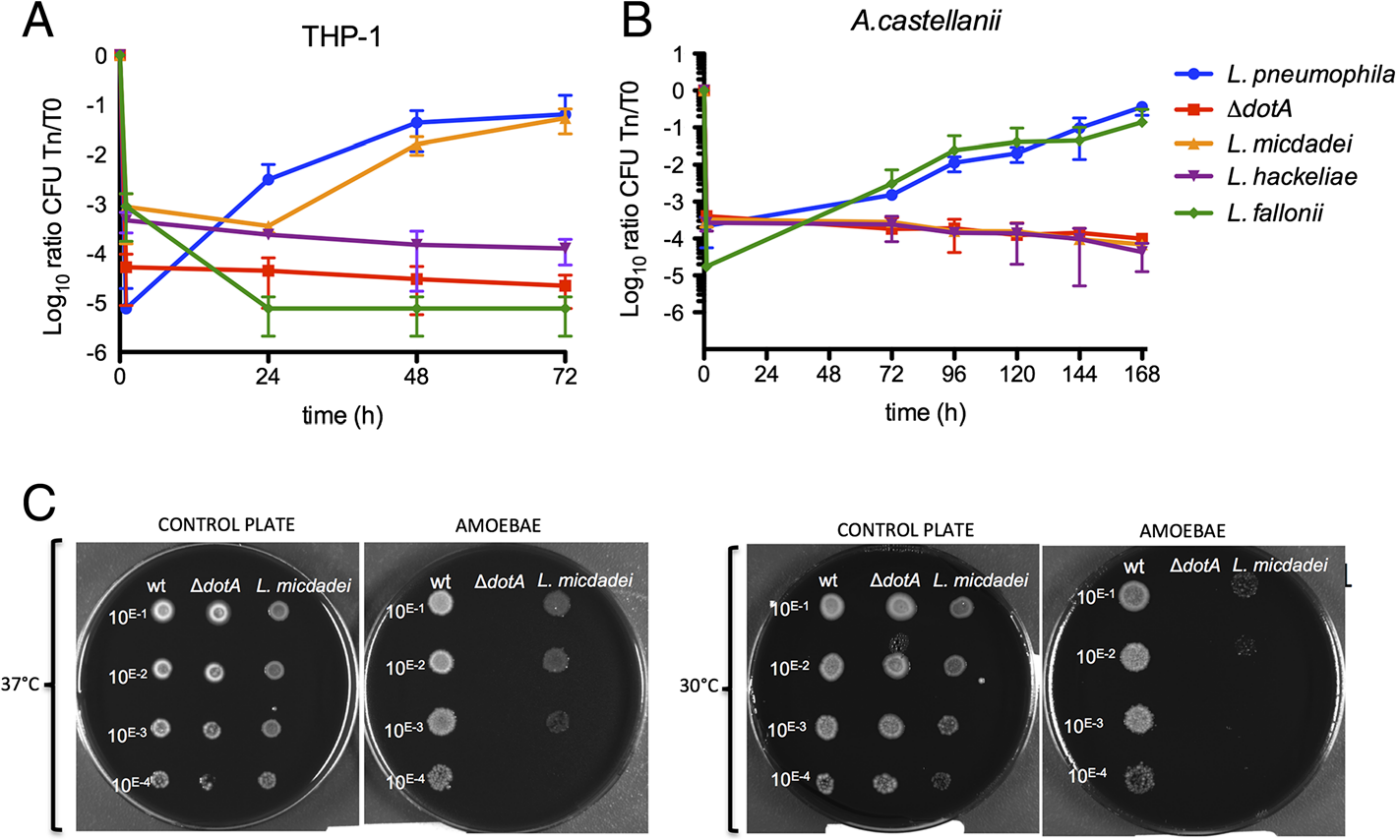
**Intracellular replication of** ***L. hackeliae*** **,** ***L. micdadei*** **and** ***L. fallonii*** **(LLAP10). (A)** THP-1 derived macrophages at 37°C. **(B)** *A. castellanii* culture at 20°C. **(C)** *A. castellanii* plate test at 37°C and 30°C. *L. pneumophila* strain Paris wild type (wt) and ∆*dotA* were used as positive and negative controls, respectively. Intracellular replication for each strain was determined by recording the number of colony-forming units (CFU) through plating on BCYE agar. Blue, *L. pneumophila* strain Paris; red, ∆*dotA*; orange, *L. micdadei*; violet, *L. hackeliae*; green, *L. fallonii* (LLAP10). Results are expressed as Log10 ratio CFU Tn/T0 and each point represents the mean ± standard deviation of two or three independent experiments. The error bars represent the standard deviation, but some were too small to clearly appear in the figure.

Little to nothing is known about the environmental distribution and the virulence of different *Legionella* species for human cells. Similarly, it is not known why *L. pneumophila* and *L. longbeachae* are so predominant in human disease compared with other *Legionella* species. As a first step to understand these differences we analyzed the capacity of *L. micdadei*, *L. hackeliae* and *L. fallonii* to infect the protozoan species *Acanthamoeba castellanii* and the human monocytic cell line THP-1. As shown in Figure 1A, *L. micdadei* replicated in THP-1 cells, similar to *L. pneumophila*, while *L. fallonii* and *L. hackeliae* were unable to replicate in these cells, although they are phagocytosed efficiently as seen from the higher numbers entering the cells after one hour of infection (Figure 1A). In contrast, *L. fallonii* was able to replicate in *A. castellanii* (Figure 1B). However, neither *L. hackeliae* nor *L. micdadei* replicated in this amoeba. Thus, additional experiments are necessary to analyze whether *A. castellani* is their environmental host or not (Figure 1B). Similar results have been obtained using *Dictyostelium discoideum* as a host where *L. micdadei* can replicate in this model amoeba but *L. hackeliae* cannot [6]. In contrast, it was reported that *L. micdadei* is able to replicate in *A. castellani* [6,18]. Puzzled by these contradicting results we further analyzed the infection capacity of *L. micdadei*. Our infection assays had been carried out at 20°C whereas Hägele and colleagues [6] performed their infections at 30°C. We thought that the different results might be due to the different temperatures used. We thus carried out infection assays at 30°C and also used amoeba plate testing [19] at 37°C and 30°C (Figure 1C). Indeed, *L. micdadei* was able to replicate in *A. castellani* at 37°C and also at 30°C, although to a lesser extent compared with *L. pneumophila* (Additional file 1). This suggested that the replication capacity of *L. micdadei* in *A. castellanii* is temperature dependent.

Taken together the replication capacity of the different *Legionella* species in amoeba and human cells differed in a way similar to the epidemiological data for these species. This suggests that common as well as species-specific mechanisms might be involved in *Legionella* infection and replication in human cells.

### The *Legionella* genomes have similar genomic features but very different genome content

**Figure 2:**
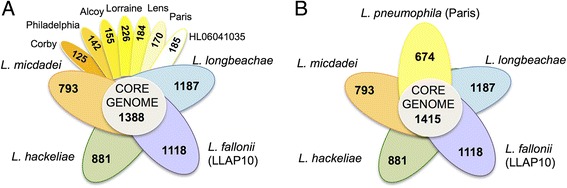
**Shared and specific content of the different** ***Legionella*** **species/strains analyzed in this study.** Each petal and color represents one genome. The number in the center of the diagram represents the orthologous genes shared by all the genomes. The number inside of each individual petal corresponds to the specific genes of each genome with non-orthologous genes in any of the other genomes. **(A)** Core genome of five *Legionella* species including seven *L. pneumophila* genomes. **(B)** Core genome when one representative of each *Legionella* species is taken into account.

At approximately 3.5 Mb, the genome sizes of *L. hackeliae* and *L. micdadei* are similar to that of *L. pneumophila* whereas that of *L. fallonii* is similar to that of *L. longbeachae* at approximately 4 Mb (Table 1). The GC content is highly homogenous (approximately 39%) and the gene order is relatively well conserved. Apart from *L. micdadei*, each strain contained one or two plasmids between 14 and 238 kb in size (Table 1). When five different *L. pneumophila* genomes were compared the pan-genome comprised 2,957 genes, the core-genome of the species *L. pneumophila* contained 1,979 genes and the calculation of the rarefraction curves indicated that *L. pneumophila* has an open pan-genome [11]. This held true when we analyzed 11 *Legionella* genomes here (seven *L. pneumophila* strains and one strain each of *L. longbeachae*, *L. micdadei*, *L. hackeliae* and *L. fallonii*); the *Legionella* pan-genome increased considerably to 9,194 genes and the core genome was 1,388 genes (Figure 2A) or 1,415 genes when comparing one strain of each sequenced species (*L. pneumophila* Paris as representative) (Figure 2B). Thus, the core genome of *Legionella* represents only about 15% of the pan-genome, indicating that the *Legionella* accessory genome is large. The complete annotation of these three newly sequenced genomes is available in the LegionellaScope database [20] and at the Institut Pasteur, LegioList [21].

**Table 1:** **General features of the** ***L. fallonii*** **,** ***L. micdadei*** **and** ***L. hackeliae*** **genomes compared with** ***L. pneumophila*** **and** ***L. longbeachae***

	***L. pneumophila***	***L. longbeachae***	***L. fallonii*** **LLAP10**		***L. micdadei***	***L. hackeliae***
	**Paris**	**NSW150**	**ATCC700992**		**ATCC33218**	**ATCC35250**
Genome size (Mb)	3.5	4.1	4.2		3.3	3.4
G + C content (%)	38	37	38		40	39
Genes	3,178	3,660	3,673		3,076	3,185
Protein coding genes	3,079	3,571	3,601		3,009	3,103
Pseudogenes	45	23	7		11	20
tRNA	43	46	46		43	43
16S/23S/5S	3/3/3	4/4/4	4/4/4		3/3/3	4/4/4
Coding density (%)	87	84	87		90	89
Plasmids	1	1	2		0	1
	Plasmid	Plasmid	Plasmid			Plasmid
			A	B		
Size (kb)	131.8	71.8	238.8	14.6		129.9
G + C content (%)	37	38	39	36		38
Genes	142	75	248	15		141
Protein coding genes	140	75	247	15		141
Pseudogenes	2	0	1	0		0
Coding density (%)	91	86	89	85		91

**Figure 3:**
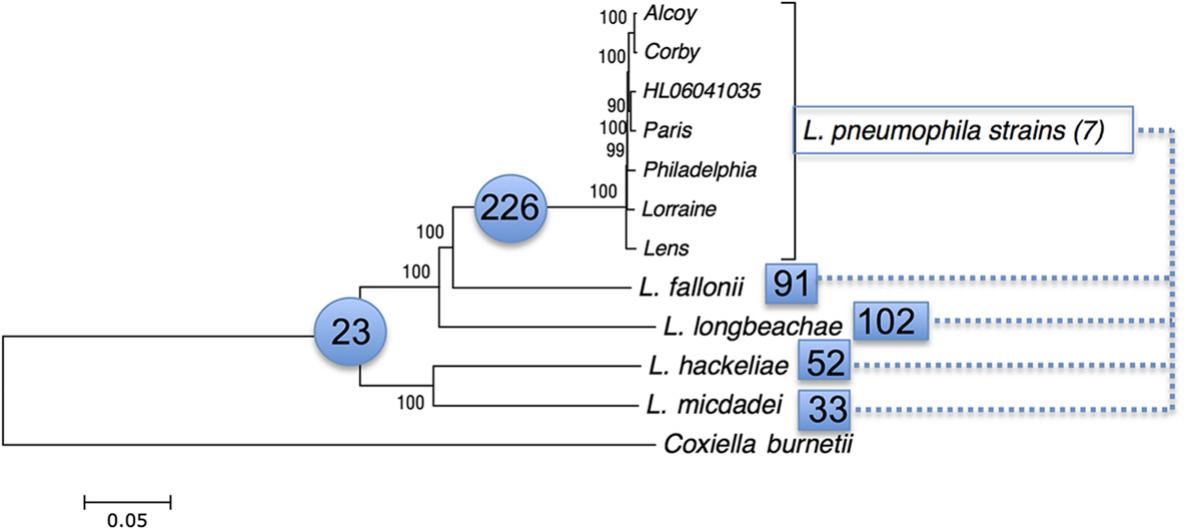
**Phylogenetic tree of six** ***Legionella*** **species and seven** ***L. pneumophila*** **strains and their shared Dot/Icm substrates.** Neighbor-joining tree based on the concatenation of 816 protein-coding genes from 11 *Legionella* genomes. *C. burnetii* was used as out-group. The tree was constructed using MEGA and JTT as model of evolution. The values above nodes indicate the bootstrap values. The values in blue circles represent the number of Dot/Icm substrates shared by the species in the corresponding cluster, suggesting that they were present in the common ancestor. The values inside blues squares are the number of Dot/Icm substrates shared between *L. pneumophila* strains and the remaining species (for example, the species *L. micdadei* and *L. pneumophila* share 33 Dot/Icm substrates).

To establish a whole genome-based phylogeny of these *Legionella* species we used either 29 housekeeping genes or 816 orthologous genes shared among the 11 *Legionella* strains analyzed. *Coxiella burnetii* was used as outgroup. Phylogenetic reconstructions using either the nucleotide or the amino acid sequences gave the same tree topology for the different species. In contrast, the tree topology of the *L. pneumophila* strains was different depending on the data set or the phylogenetic method used, probably due to the high recombination rate of this species [12,22]. Our phylogenetic analyses showed that *L. pneumophila*, *L. fallonii* and *L. longbeachae* group together, with *L. fallonii* being phylogenetically the closest to *L. pneumophila. L. micdadei* and *L. hackeliae* formed a second cluster (Figure 3). Except for the place of *L. fallonii*, this is in agreement with previous phylogenies of the genus *Legionella* [23,24]*.* In previous work *L. pneumophila* was described as phylogenetically closer to *L. longbeachae* than to *L. fallonii* [25] or *L. fallonii* closer to *L. longbeachae* than to *L. pneumophila* [26]. However, these studies are based on 16S RNA sequences and bootstrap values associated with the corresponding nodes to evaluate its statistical support are not provided.

In conclusion, the general features of the *Legionella* genomes are very similar but each *Legionella* species has a distinctive genomic content with about 60% of genes being species-specific. Interestingly, human pathogenic and non-pathogenic species were mixed in the phylogeny, which indicates that virulent traits favoring human infection have been acquired independently during the evolution of the genus.

### Type II and IVB secretion systems are part of the core genome of *Legionella*

Like in other bacterial genera, the core genome of *Legionella* contains the genes encoding fundamental metabolic pathways and the ribosomal machinery. In addition, the Dot/Icm type IVB secretion system (T4BSS) as well as the Lsp type II secretion system (T2SS), both indispensable for intracellular replication, also belong to the core genome of this genus. The chromosomal organization of the Dot/Icm and the Lsp secretion system is also conserved, except for the genes *icmD* and *icmC*, which are duplicated in *L. fallonii*. Interestingly, the degree of conservation of the different Dot/Icm proteins is very variable, ranging from >90% for DotB to proteins without any homology such as IcmR. Surprisingly, DotA, an integral inner membrane protein [27] indispensable for intracellular growth [28], is one of the least conserved proteins of the Dot/Icm T4SS (Additional file 2). Unexpectedly, the sequenced *L. hackeliae* strain (ATCC35250) had a stop codon in the gene coding for DotA, splitting it into 984 and 2,040 nucleotide fragments. Resequencing of the *dotA* gene confirmed the presence of the stop codon. As this strain was not able to replicate in *A. castellanii*, we thought that this might be due to the mutated *dotA* gene leading to a non-functional T4SS. To verify if this mutation was specific for the sequenced strain, we analyzed the *dotA* gene in a second *L. hackeliae* strain (ATCC35999). In this strain the *dotA* gene was intact. Thus, the *dotA* gene fragmentation in the sequenced strain probably occurred during storage. However, when testing the virulence of both *L. hackeliae* strains in *A. castellanii* using the amoeba plate test, neither were able to replicate at 30°C or at 37°C (data not shown). To analyze if the Dot/Icm secretion system was functional in the sequenced strains, we used the calmodulin-dependent adenylate cyclase (CyaA) gene fusion approach [29] and RalF from *L. pneumophila* [30] for *L. hackeliae*, *L. micdadei* and *L. fallonii.* However, several attempts to show secretion of RalF in one of these strains failed, as RalF was never expressed in them despite testing under several different conditions. Thus, further experiments are necessary to adapt this assay to the here newly sequenced *Legionella* species.

Another particularity of the Dot/Icm system is the *icmR* gene. Indeed, similar to what was reported for *L. hackeliae* and *L. micdadei* where *icmR* was replaced by a non-homologous gene with functional equivalence [31,32], a gene encoding a protein with no similarity to any previously described protein is present in the position of *icmR* in *L. fallonii*, possibly serving as a functional equivalent of *icmR* of *L. pneumophila.* Other variable genes include *icmX* and *icmG*. IcmG has been described as a component that interacts with the effector proteins [33], which may explain the high variability in different species. In contrast, the components *dotB*, *icmS*, *icmW* and *icmP* are highly conserved. Indeed, these four genes can functionally replace their homologues in *C. burnetii* [34].

### The *L. micdadei*, *L. hackeliae* and *L. fallonii* genomes encode surprising functions

#### L. fallonii *is able to synthesize cellulose*

**Figure 4:**
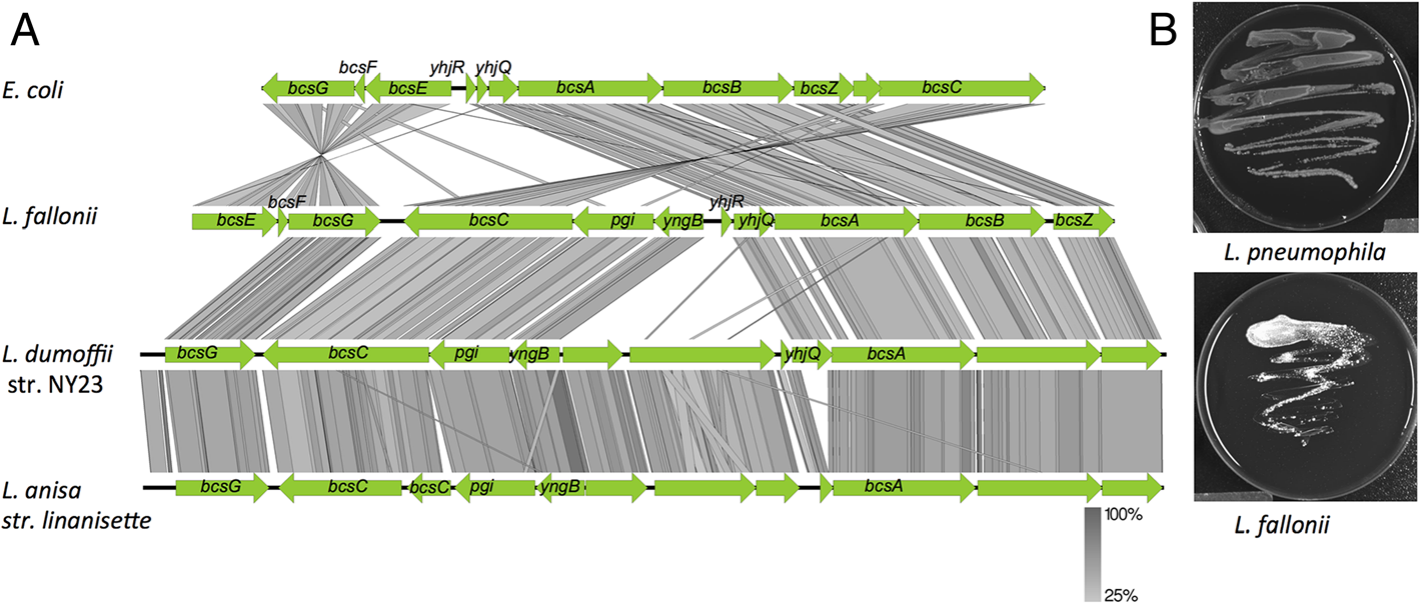
***L. fallonii*** **synthesizes cellulose. (A)** Genomic organization and Blastx comparison of the regions encoding the cellulose synthesis machinery in *E. coli*, *L. fallonii*, *L. dumofii* and *L. anisa*. The gray color code represents the Blast matches; the darker the gray the better the blast match. **(B)** Growth of *L. fallonii* on calcofluor agar plates that shows cellulose synthesis as visualized under long-wave UV light. *L. fallonii* is fluorescent due to the binding of calcofluor to cellulose. In contrast *L. pneumophila* that was used as negative control is not.

Enzymes degrading cellulose have been described in *L. longbeachae* and were also found in *L. fallonii.* However, in addition the *L. fallonii* genome encodes a complete machinery for the synthesis of cellulose (Figure 4A). Although the bacterial need for cellulose may be surprising, cellulose has been reported as a common component of biofilms of several bacterial species such as *Salmonella enterica* or *Escherichia coli* [35]. The bacterial genes for cellulose synthesis are called *bcsABZ*C. In *S. enterica* and *E. coli* a second operon necessary for cellulose biosynthesis named *bcsEFG* is present [35,36]. Both clusters (from *lfa3354* to *lfa3363* and *lfa2987* to *lfa2988*) are present in *L. fallonii*, although with some differences in organization (Figure 4A). To analyze whether *L. fallonii* is able to synthesize cellulose, we used agar plates containing calcofluor, which binds cellulose and leads to fluorescence under UV radiation. Indeed, *L. fallonii* showed strong fluorescence under long-wave UV light, in contrast to *L. pneumophila* (Figure 4B), demonstrating cellulose biosynthesis in the genus *Legionella* for the first time. A blast search identified genes homologous to the *L. fallonii* cellulose operon (except *bcsE* and *bcsF*) also in the draft genome sequences of *L. anisa* and *L. dumoffii* (Figure 4A)*.* This suggests that several *Legionella* species have the capacity to synthesize cellulose.

### L. fallonii *possesses genes encoding hopanoid biosynthesis and antibiotic resistance*

*L. fallonii* encodes genes for hopanoid biosynthesis currently not found in any other *Legionella* species. About 10% of all sequenced bacteria contain genes for hopanoid synthesis, in particular cyanobacteria, acetobacter, streptomycetes, methylotrophs and purple non-sulfur bacteria. Hopanoids have been proposed to enhance membrane stability and to decrease membrane permeability [37], similar to sterols in eukaryotic cell membranes [38]*.* In *Burkholderia cenocepacia* these genes are involved in sensitivity to low pH, detergent and antibiotics, and are related to motility [39]. In *Streptomyces coelicolor*, this cluster has been well studied. Although not all genes of the *S. coelicolor* cluster are conserved in *L. fallonii* (Additional file 3), to date all bacteria carrying the gene for squelene-hopene-cyclase produce hopanoids [39]. As *L. fallonii* also carries this gene, we expect this species is able to synthesize hopanoids, although their function in this species remains unknown.

Another peculiarity of *L. fallonii* is that it contains several antibiotic resistance genes not previously described in *Legionella*, including one encoding a chloramphenicol acetyltransferase (*lfa0269*) that is predicted to catalyze the acetyl-CoA-dependent acetylation of chloramphenicol. Furthermore, we identified a gene likely involved in erythromycin resistance, *ereA* (*lfa1884*) that is present also in *L. drancourtii* and *L. dumoffii.* This gene is located in gene clusters related to DNA mobility, such as integrases or prophage-related genes, and are rich in ELPs and repeats. These features indicate that these regions are putative genomic islands (Additional file 4).

### L. hackeliae *and* L. fallonii *encode chitin deacetylase activity*

*L. hackeliae* and *L. fallonii* each contain a different gene coding for a chitin deacetylase (*lha3256*/*lfa0697*), an enzyme involved in deacetylation of chitin. An *in vitro* test described by Vadake [40] suggests that *L. fallonii* does have chitin deacetylase activity whereas it was not possible to demonstrate this clearly for *L. hackeliae* (Additional file 5). Chitin, a homopolymer of N-acetyl-glucosamine, is one of the most abundant polymers in the Earth’s biomass, especially in marine environments. Interestingly it is also a component of the cyst wall of *Entamoeba invadens*, and enzymes responsible for chitin synthesis have been found in *Entamoeba* genomes [41]. The presence of chitin or chitin synthases has not been described in other protozoan genomes, but very few genomes of this group have been sequenced yet. Thus, chitin may be a common component of protozoa that are able to encyst. Although the other *Legionella* genomes analyzed here do not encode chitin deacetylase activity, all *Legionella* genomes encode chitinases. Chitinases are chitin-degrading enzymes leading to low molecular weight chito-oligomers whereas chitin decetylase degrades chitin to chitosan. Both products are of interest for industry and there is growing interest in organisms that produce chitosan. *Legionella* may be a new possible source of chitosan production.

### L. micdadei *contains the first putative complete prophage identified in a* Legionella *genome*

The analysis of the unique genes from *L. micdadei* identified a specific region encoding 73 proteins, at least 16 of which are phage-associated proteins that represent a putative complete prophage (Additional file 6). This region contains genes encoding the phage capsid tail and replication proteins. Complete prophages have never been described in *Legionella* despite the frequent presence of phage-related proteins scattered in their genomes. Most attempts to isolate prophages that exclusively infect *Legionella* have also failed, until recently when two groups isolated *Legionella* bacteriophages [42,43] from environmental water samples and organs of guinea pigs. Thus, *Legionella* do have phages, but they seem to be rare.

### L. fallonii *and* L. micdadei *contain additional flagella operons*

**Figure 5:**
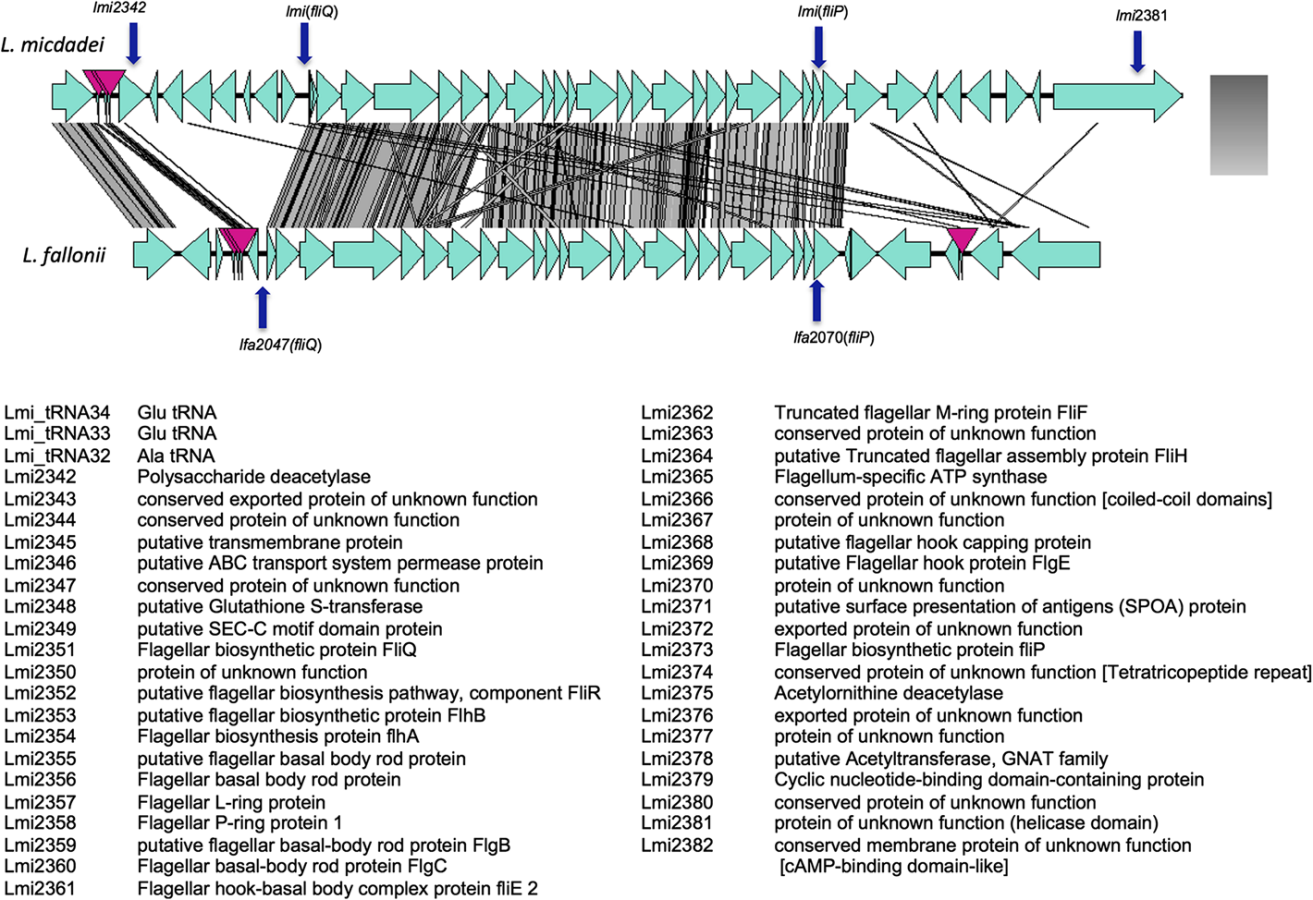
**The** ***L. micdadei*** **and** ***L. fallonii*** **genomes contain specific flagellar-encoding regions** ***.*** Genomic organization and Blastx comparison of the specific flagellar gene clusters in *L. micdadei* and *L. fallonii*. The gray color code represents the Blast matches; the darker the gray the better the blast match. Pink arrows point to tRNA genes. Protein names and their predicted function in *L. micdadei* are indicated below.

The comparison of the *L. pneumophila* and *L. longbeachae* genomes revealed that *L. longbeachae* does not contain genes allowing flagella biosynthesis [9]. As recognition of flagellin by Naip5 initiates host immune responses that control *L. pneumophila* infection in certain eukaryotic cells [44,45], the presence or absence of flagella is important for intracellular replication of *Legionella. L. hackeliae*, *L. fallonii* and *L. micdadei* also contain three flagella operons homologous to those described in *L. pneumophila* (Figure S5A-C in Additional file 7). Interestingly *L. fallonii* and *L. micdadei* encode a fourth region not previously described in any sequenced *Legionella* species that might also code for flagella (Figure 5).

### A highly dynamic mobilome characterizes the *Legionella* genomes

Genomic elements like plasmids, genomic islands or transposons constitute the mobilome of a genome. All *Legionella* species analyzed contain many of these mobile elements. For example, *L. hackeliae* possesses a plasmid of 129.88 kb whereas *L. fallonii* (LLAP10) contains two plasmids of 238.76 kb and 14.57 kb, respectively (Table 1). Furthermore, the plasmid present in *L. hackeliae* is identical to the *L. pneumophila* strain Paris plasmid (100% nucleotide identity over the entire length except for two transposases in the strain Paris plasmid; Additional file 8). This suggests that this plasmid has recently moved horizontally between both species, which is a new example of the high rate of gene transfer among *Legionella* genomes [46,47].

In addition to the plasmids identified and their evident exchange among strains and species, a hallmark of the *Legionella* mobilome is the presence of many different type IVA secretion system-encoding regions in the plasmids as well as in genomic island-like regions on the chromosome. Interestingly, these regions often encode *tra*-like genes with considerable homology among the different strains. However, each new strain analyzed contained new regions, underlining the high diversity of these systems in the *Legionella* genomes. Predominant are F-type and P-type IVA systems that code for conjugative pili that allow mating. F-type IVA secretion systems are present on all *L. pneumophila* plasmids, the *L. hackeliae* plasmid, the 238 kb *L. fallonii* plasmid (two systems) and on the chromosomes of *L. pneumophila* strain Philadelphia, *L. longbeachae* and *L. fallonii* (Additional file 9). Each encodes a homologue of the global regulator CsrA, named LvrC, which when present in the chromosome also encodes the *lvrRAB* gene cluster. This was recently described as being involved in the regulation of excision of the ICE Trb1 of *L. pneumophila* strain Corby [48]. Thus, conjugative exchange of DNA has an important role in *Legionella* and is one key factor enabling *Legionella* to rapidly adapt to changing conditions.

The mobility and horizontal transfer of these different regions are further shown when studying the distribution of these systems. For example, the *lvh* cluster, a type IVA system involved in virulence under conditions mimicking the spread of Legionnaires’ disease from environmental niches [49], is also present in *L. micdadei*, in one of the two completely sequenced *L. longbeachae* strains and in five of the completely sequenced *L. pneumophila* strains (Table 2). In addition, the so-called GI-T4SS recently described in strain *L. pneumophila* 130b [13], and first recognized in *Haemophilus influenzae* as a T4SS involved in propagation of genomic islands [50], is believed to play an important role in the evolution and adaptation of *Legionella* [51]. GI-T4SS clusters were found to be conserved in *L. pneumophila*, with two clusters each in strains Corby, Paris, 130b and HL06041035, and one in each of Alcoy, Philadelphia, Lens and Lorraine [51], as well as in strains of *L. longbeachae*, *L. hackeliae*, *L. micdadei* and *L. fallonii* (Table 2). Thus, a heterogeneous distribution among species and strains testifies to the continuous exchange of these elements among *Legionella*, contributing to the plasticity and dynamic nature of their genomes.

**Table 2:** **Distribution of type IV secretion systems in the analyzed** ***Legionella*** **genomes**

**Type IV secretion systems**		**Type IVB**	**Type IVA**			**GI-type**
**Chromosome**		**Dot/Icm**	**P-type**	**F-type**	**Lvh**	
*L. pneumophila*	Paris	1	-	-	1	2
	Lens	1	-	-	1	1
	Philadelphia	1	-	1	1	1
	Corby	1	2	-	-	2
	Lorraine	1	1	-	-	1
	HL06041035	1	-	-	1	2
	130b	1	1		2	2
*L. longbeachae*	NSW-150	1	1	1	-	-
*L. longbeachae*	D-4968	1	-	1	1	1
*L. hackeliae*	ATCC35250	1	-	-	-	1
*L. micdadei*	ATCC33218	1	-	-	1	1
*L. micdadei*	02/42	1	1	-	1	-
*L. fallonii* (*LLAP10*)	ATCC700992	1	-	1	-	1
**Plasmids**						
*L. pneumophila*	Paris	-	-	1	-	-
	Lens	-	-	1	-	-
	Lorraine	-	-	1	-	-
*L. hackeliae*		-	-	1	-	-
*L. fallonii*		-	-	2	-	-

### *L. micdadei* strains from different geographical regions are highly similar except for their mobilome

**Figure 6:**
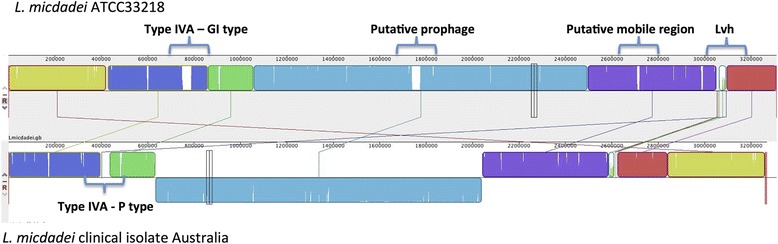
**Genome comparison of two** ***L. micdadei*** **strains** ***.*** The complete genome sequences of the two *L. micdadei* strains included in this study were aligned using the software Mauve. The two strains align perfectly with the exception of three mobile genetic elements specifically present in strain *L. micdadei* ATCC33218 and one specifically present in the Victorian isolate. The specific regions of each genome are indicated. The 'Lvh region' is indicated, as this region is, with a high number of SNPs, quite divergent between the two isolates.

To investigate the genomic diversity of the species *L. micdadei*, we determined the draft genome sequence of a clinical isolate obtained from the Microbiological Diagnostic Unit Public Health Laboratory (MDU), Australia and compared it with the completely sequenced strain *L. micdadei* ATCC 33218. The genome size and GC content of the two *L. micdadei* strains were highly similar (Figure 6). The main differences between the two *L. micdadei* strains were mobile genetic elements. Furthermore, the number of SNPs (1,985 SNPs) was very low, similar to serogroup 1 strains of *L. longbeachae* (1,611 SNPs) [9]. This is strikingly different to *L. pneumophila* where two different strains may contain more than 30 000 SNPs. This suggests that *L. micdadei* and *L. longbeachae* evolved more recently compared to the *L. pneumophila*. Three large regions of the *L. micdadei* ATCC 33218 genome are absent from the Australian isolate (Figure 6). One is a genomic island encoding a GI-T4SS (36 kb), one is the predicted prophage we identified in this study, and another is a smaller cluster of approximately 9 kb that is flanked by three tRNA genes and that contains phage-related genes and a gene associated with abortive infection system (Figure 6). Similarly, in the Australian isolate a cluster absent from the completely sequenced *L. micdadei* strain corresponds to a P-type IVA secretion system. Interestingly, the Lvh region, encoding a T4ASS that is highly conserved among all strains and species analyzed to date, is divergent in the two *L. micdadei* strains with a high number of SNPs (Additional file 10). Thus, the main genetic differences between these two closely related *L. micdadei* strains are mobile genetic elements, further underlining the large extent of horizontal gene transfer that is present in the genus *Legionella*.

### The core set of Dot/Icm effectors is small with only 24 conserved substrates

*L. pneumophila* encodes over 300 proteins that are translocated into the host cell by the Dot/Icm T4SS (Additional file 11). Their conservation is high among different *L. pneumophila* strains, as 77% of these substrates are present in all *L. pneumophila* strains sequenced to date. Interestingly, when the Dot/Icm substrates of *L. pneumophila* and *L. longbeachae* are compared, only 35% (101) are present in both species [9]. Interestingly, the *L. longbeachae* and *L. pneumophila* genomes contain the highest number of common substrates, although *L. fallonii* is phylogenetically closer to *L. pneumophila* than to *L. longbeachae* (Figure 3). When investigating the presence of these substrates in five *Legionella* species by adding the *L. hackeliae*, *L. micdadei* and *L. fallonii* genomes, this revealed that their conservation is very low (Figure 3). With 33 conserved substrates, the lowest number is shared between *L. micdadei* and *L. pneumophila.* This result suggests that the shared substrates might relate to similar environmental niches or virulence properties (*L. pneumophila* and *L. longbeachae*) than to a closer phylogenetic relationship.

The Dot/Icm substrates conserved in all *Legionella* species are probably those indispensable for intracellular replication and are important players in host-pathogen interactions. Most surprisingly, only 24 of the 300 described substrates of *L. pneumophila* are present in all five *Legionella* species and most of these are of yet unknown function (Table 3). However, a third of the conserved substrates contain eukaryotic motifs like ankyrin or Sel-1 domains or TPR repeats. Others were previously defined as ELPs, such as the sphingomyelinase-like phosphodiesterase. Among the substrates that have been investigated further are VipF, which causes growth defects in *S. cerevisae*, and several of the ankyrin repeat motif proteins. VipF inhibits lysosomal protein trafficking [52] and AnkH was shown to play a role in intracellular replication of *L. pneumophila* in macrophages and protozoa and in intrapulmonary proliferation in mice [53]. The function of MavBFNQ and RavC is not known, but they have been recovered in screens for vacuolar localization and have been shown to co-localize with SidC at the *L. pneumophila* vacuole [54].

**Table 3:** **Core of substrates of the Dot/Icm secretion system present in 11** ***Legionella*** **genomes**

**Label**	**Product and associated motif**	**Gene name**	**Amino acid identity** ^**a**^			
			***L. fallonii***	***L. long.***	***L. micdadeii***	***L. hack***
*lpg0021*/*lpp0021*	Protein of unknown function [ribosome associated protein]		**75**	**75**	**68**	**68**
*lpg0103*/*lpp0117*	N-terminal acetyltransferase, GNAT family	*vipF*	**62**	**61**	**50**	**47**
*lpg0107*/*lpp0121* ^b^	Conserved protein of unknown function	*ravC*	**84**	**80**	**72**	**47**
*lpg0140*/*lpp0155*	Membrane protein of unknown function	*cetLP1*	**53**	**45**	**39**	**36**
*lpg0376*/*lpp0443*	Protein of unknown function	*sdhA*	**60**	**48**	**38**	**38**
*lpg0405*/*lpp0471*	Protein of unknown function		**56**	**56**	**50**	**40**
*lpg0483*/*lpp0547*	Protein of unknown function [ankyrin]^c^	*legA12*/*ankC*	**55**	**49**	**31**	**29**
*lpg1356*/*lpp1310*	Putative Beta-lactamase [Sel1-domain]^c^[TPR repeat]^c^	*EnhC* homologue	**80**	**71**	**67**	**69**
*lpg1578*/*lpp4178*	Protein of unknown function		**50**	**37**	**25**	**24**
*lpg1661*/*lpp1632*	Heparan-alpha glucosaminide N-acetyltransferase^d^		**60**	57	47	48
*lpg1663*/*lpp1634*	Protein of unknown function	*cetLP3*	**54**	44	**40**	58
*lpg1752*/*lpp1716*	Protein of unknown function	*mavB*	**41**	**40**	**27**	**26**
*lpg1907*/*lpp1882*	Membrane protein of unknown function [hydrolases]		**54**	**52**	44	49
*lpg2300*/*lpp2248*	Protein of unknown function [ankyrin]^c^	*legA3*/*ankH*/*ankW*	**81**	**76**	**71**	**75**
*lpg2322*/*lpp2270*	Protein of unknown function [ankyrin]	*legA5*/*ankK*	**57**	**51**	**39**	42
*lpg2351*/*lpp2300*	Protein of unknown function	*mavF*	**56**	**50**	**33**	33
*lpg2359*/*lpp2308*	Protein of unknown function [putative GatB/Yqey domain]^c^		**82**	**79**	73	72
*lpg2588*/*lpp2947*	Acid sphingomyelinase-like phosphodiesterase	*legS1*	**64**	**61**	54	33
*lpg2591*/*lpp2644*	Protein of unknown function	*ceg33*	**46**	**47**	**34**	**26**
*lpg2815*/*lpp2867*	Membrane protein of unknown function	*mavN*	**62**	**64**	**54**	**52**
*lpg2832*/*lpp2889*	Protein of unknown function		**72**	**68**	**60**	**62**
*lpg2888*/*lpp2947*	Membrane protein of unknown function [coiled-coil]^c^		**59**	**50**	**34**	**40**
*lpg2936*/*lpp3004*	Ribosomal RNA small subunit methyltransferase E		**67**	**67**	**60**	**60**
*lpg2975*/*lpp3047*	Protein of unknown function	*mavQ*	**58**	**57**	**33**	**30**

^a^Amino acid identity with respect to *L. pneumophila* strain Philadelphia. ^b^ *ravC* has been erroneously named as *lpg0170* in previous publications. ^c^The motif is present in all orthologues. ^d^High prediction for enzymatic activity in all the corresponding orthologues (prediction based on PRIAM EC number). Bold numbers indicate synteny between *L. pneumophila* genes and the corresponding orthologous gene.

SdhA, a *L. pneumophila* effector that is necessary for full virulence of this species, is a particular case. It is present in all *Legionella* analyzed but the similarity with *L. longbeachae* is small and thus below the cutoff established for our orthologous search (at least 65% of the length of the compared protein). However, given that homologues with a significant similarity are present in all species in synteny (except for *L. hackeliae*), and coiled-coil motifs are detected in all, SdhA was also defined as a core effector. Moreover, SdhA has been shown to be necessary for infection of mice and in *Galleria mellonella* [55,56]. Surprisingly, the effector SidJ is not part of the core set of *Legionella* substrates, although its deletion led to a strong replication defect in eukaryotic cells. However, SidJ is present in *L. pneumophila* and *L. longbeachae*, the major human pathogens.

Interestingly, the growth defect of strains lacking SdhA and SidJ seems more important in mice and human macrophages than in amoeba. Replication of the *sdhA* mutant is severely impaired in mouse bone marrow-derived macrophages but less in the amoeba *Dictyostelium discoideum* [56]. Similarly, a *∆sidJ* strain shows significant growth defects in both macrophages and amoebae, but replication in macrophages is affected from the start of the infection, whereas the growth defect in amoebae is evident only after 72 h of infection and was less pronounced [57]. These data may suggest that effectors important in human infection are not necessarily essential in the protozoan hosts and thus certain effectors might be important for human infection even though no growth defect in protozoan infection is detectable.

### Eukaryotic-like proteins are a specific feature of the genus *Legionella*

One feature shared by many of the substrates of the Dot/Icm secretion system is the presence of eukaryotic motifs (EMs). Indeed, of 55 proteins of *L. pneumophila* Philadelphia encoding EMs, 45 (82%) are confirmed substrates of the Dot/Icm secretion system (Additional file 12). Thus, we searched for proteins containing EMs in all sequenced genomes. In the five *Legionella* species we identified 218 proteins with eukaryotic domains (Additional file 13). The genomes of *L. longbeachae* and *L. fallonii* contain nearly twice as many proteins with EMs as the other genomes, probably due to their larger genome size. The ankyrin motif is the most frequent one, followed by long coiled-coil domains. Some EMs that were described remain specific for *L. longbeachae*, such as the PPR repeats, PAM2 domain or the phosphatidylinositol-4-phosphate 5-kinase, indicating that they are probably related to its particular habitat in soil [9]. In contrast, proteins with tubulin-tyrosine ligase domains (LLo2200), probably involved in the posttranslational modification of tubulin [58], are absent only from *L. pneumophila*. With the aim to analyze whether additional eukaryotic motifs not yet identified are present in the *Legionella* genomes, we developed a strategy allowing for a comprehensive scan of all genomes. First we searched the Interpro database for all motifs, which occur in at least 85% of proteins from eukaryotic genomes and only 15% or less in proteins from prokaryotic genomes. Using this criterion, we obtained 8,329 motifs that were considered as eukaryotic (see Materials and methods). All predicted *Legionella* proteins were scanned for these motifs. This approach allowed us to identify 10 EMs not described before in *Legionella*, including thaumatin, RhoGTPase and DM9 domains (Table 4). Interestingly, thaumatin-like proteins accumulate in plants in response to infection by pathogens and possess antifungal activity [59,60] and a *Drosophila* DM9-containing protein is strongly up-regulated after infection of *Drosphila* larvae by *Pseudomonas* species [61]. Many of these new EMs are only present in the newly sequenced genomes, such as synaptobrevin, an intrinsic membrane protein of small synaptic vesicles [62] or the clathrin/coatomer adaptine-like domain that is associated with transport between the endoplasmic reticulum and Golgi [63]. Given their function in eukaryotic organisms, these protein domains might indeed be important in host-pathogen interactions.

**Table 4:** **Genes encoding proteins containing eukaryotic motifs not previously described**

***L. pneumophila***	***L. longbeachae***	***L. micdadei***	***L. hackeliae***	***L. fallonii*** **(LLAP10)**	**Product**	**Eukaryotic motif**
*lpg0095*/*lpp0109*	*llo3322*	*lmi1217*	*lha1000*	*lfa0120*	Cytosolic IMP-GMP specific 5'-nucleotidase	HAD-superfamily hydrolase, 5'-nucleotidase
*lpg0301*/*lpp0379*				*lfa0312#*	Protein of unknown function	DM9 repeat
*lpg0971*/*lpp1033*					Apyrase	Nucleoside phosphatase
*lpg1328* ^a^/*lpp1283*				*lfa0766*	Protein of unknown function	Thaumatin
*lpg1798* ^a^/*lpp1761*	*llo0991*				Protein of unknown function	RhoGTPase
*lpg1905*/*lpp1880*	*llo1247*	*lmi2007*	*lha1318*	*lfa0744*	Apyrase	Nucleoside phosphatase
			*lha0318*		Protein of unknown function	DM9 repeat
	*llo3014*			*lfa2606*	Protein of unknown function	DM9 repeat
			*lha0498*		Protein of unknown function	Synaptobrevin/V-snare
				*lfa3165*	Protein of unknown function	Synaptobrevin/V-snare
	*llo0044*				Protein of unknown function	Thaumatin
		*lmi1344*			Protein of unknown function	Thaumatin
			*lha2013*		Protein of unknown function	Thaumatin
		*lmi0545*	*lha2362*	*lfa2256*	Protein of unknown function	DH/RhoGEF
	*llo0042*			*lfa0022*	Putative serine carboxypeptidase	Peptidase S28
				*lfa1003*	Protein of unknown function	Mitochondrial substrate/solute carrier repeat
		*lmi0716*			Protein of unknown function [ARM repeat]	Clathrin/coatomer_adaptine_like
		*lmi1174*			Protein of unknown function [HEAT-REPEAT DOMAIN] [F-box domain]	Clathrin/coatomer_adaptine_like
		*lmi0037*			Protein of unknown function [coiled-coil]	RhoGTPase
		*lmi0496*		*lfa2567* ^b^	Protein of unknown function	T-complex protein 10
		*lmi3046*	*lha3117* ^b^		Protein of unknown function	Peptidase C65

^a^Gene encoding a protein that has been demonstrated to be secreted by the Dot/Icm secretion system. ^b^Orthologous proteins in which the corresponding motif is not present.

#### Many eukaryotic proteins are indeed transferred horizontally from eukaryotes

Not all proteins we defined as ELPs possess EMs, but certain ones are also considered eukaryotic-like as they show a high homology to eukaryotic proteins over their whole length. One of the best known examples of this type of ELP is the sphingosine-1-phosphate lyase (encoded by the gene *lpp2128*), an enzyme that in eukaryotes catalyzes the irreversible cleavage of sphingosine-1-phosphate, and that has most likely been transferred horizontally from eukaryotes [47,64,65]. With the aim to detect proteins with higher similarity to eukaryotic proteins than to prokaryotic ones and for which we can suggest a eukaryotic origin through phylogenetic analysis, we have developed a pipeline that automatically extracts those proteins from the *Legionella* pan-genome with high similarity to eukaryotic proteins (for details see Materials and methods). Using this pipeline we identified 465 proteins as putative ELPs. For each of these proteins we constructed a phylogenetic tree that was curated and analyzed manually. However, for many of the ELPs a phylogenetic reconstruction did not allow clear demonstration of eukaryotic origin. Some aligned too poorly with their eukaryotic homologues or on just a small domain. This might be due to the fact that genomes of ciliated protozoa and amoeba, the known hosts of *Legionella* from which these ELPs are most likely acquired, are underrepresented in current databases. However, for 40 of the 465 proteins that are suggested to be of eukaryotic origin, the phylogenetic reconstruction clearly showed that they had been acquired by *Legionella* through horizontal gene transfer from eukaryotes (Table 5; Figure S9A-C in Additional file 14).

**Table 5:** **Genes horizontally transferred from eukaryotic genomes to** ***Legionella*** **genomes**

***L. pneumophila***	***L. longbeachae***	***L. micdadei***	***L. hackeliae***	***L. fallonii*** **(LLAP10)**	**Name**	**Product [domain]**
*lpg0095*/*lpp0109*	*llo3322*	*lmi1217*	*lha1000*	*lfa0120*		cytosolic IMP-GMP specific 5'-nucleotidase
*lpg0422* ^a^/*lpp0489*	*llo2801*		*lha1522*	*lfa2690*		glucoamylase
*lpg0971*/*lpp1033*						ecto-ATP diphosphohydrolase II
*lpg1119*/*lpp1120*	*llo1016*			*lfa1837*		map Major acid phosphatase Map (histidine-acid phosphatase)
*lpg1155*/*lpp1157*						pyruvate decarboxylase
*lpg1565*/*lpp1522*	*llo0920*		*lha3027*			thi thiamine biosynthesis protein NMT-1
*lpg1675*/*lpp1647*	*llo3277*	*lmi0628*		*lfa1689*	*purC*	phosphoribosylamidoimidazole-succinocarboxamide synthase
*lpg1905*/*lpp1880*	*llo1247*	*lmi2007*	*lha1318*	*lfa0744*		putative Apyrase
*lpg1950* ^a^/*lpp1932*	*llo1397*				*ralF*	RalF, translocated into host cells by the Dot/Icm system
*lpg2176* ^a^/*lpp2128*			*lha1689*	*lfa3030*		sphingosine-1-phosphate lyase I
*lpg2556* ^a^/*lpp2626*	*llo2218*					protein kinase-like
*lpg2911*	*llo1568*			*lfa0029*	*cysK*	cysteine synthase A, O-acetylserine sulfhydrolase A subunit
*lpg2917*	*llo2243*	*lmi0866*	*lha0637*	*lfa1734*		conserved protein of unknown function [FADPNR domain]
*lpg2951*/*lpp3022*	*llo0076*	*lmi3045*	*lha3116*	*lfa3723*	*cysK*	cysteine synthase A, O-acetylserine sulfhydrolase A subunit
						protein of unknown function [SNARE domain]
		*lmi2545*		*lfa1498*		protein of unknown function [ank domain] [Ras domain] [coiled-coil domain]
	*llo0042*			*lfa0022*		putative Dipeptidyl-peptidase II
	*llo1314*					conserved protein of unknown function [Leucine-rich repeat domain]
	*llo1320*	*lmi2237*	*lha0011*	*lfa1070*		DWF 7-dehydrocholesterol reductase
	*llo1999*					putative Sphingomyelin phosphodiesterase
	*llo2200*	*lmi0200*	*lha3048*	*lfa0156*		protein of unknown function [Tubulin-tyrosine ligase domain)
	*llo2329*					GTP-binding protein ypt1 [Ras small GTPase, Rab type]
	*llo3082*					protein of unknown function [Mitochondrial substrate carrier]
	*llo3182*		*lha1738*			phosphoprotein phosphatase
	*llo3288*			*lfa1897*		protein of unknown function [Ras small GTPase, Rab type]
		*lmi0037*				protein of unknown function [RhoGAP motif] [coiled-coil]
		*lmi0515*				protein of unknown function [cAMP-binding domain-like]
		*lmi0854*				protein of unknown function [Ras GTPase domain]
		*lmi1956*				putative Adenosine deaminase
		*lmi1981*				phosphoprotein phosphatase
			*lha0498*			protein of unknown function [Synaptobrevin]
			*lha0511*			protein of unknown function
			*lha0998*			membrane protein of unknown function [Ras small GTPase, Rab type]
			*lha1423*			protein of unknown function [Cytochrome P450 domain]
			*lha2690*			membrane protein of unknown function [Ras small GTPase, Rab type]
				*lfa0589*		endo-1,3(4)-beta-glucanase
				*lfa1399*		protein of unknown function [H3 methylation by DOT1]
				*lfa1711*		membrane protein of unknown function [Ras small GTPase, Rab type]
				*lfa2465*		membrane protein of unknown function [Ras small GTPase, Rab type]
				*lfa2470*		protein of unknown function [SecA DEAD-like]

^a^Gene encoding a protein that has been demonstrated to be secreted by the Dot/Icm secretion system.

**Figure 7:**
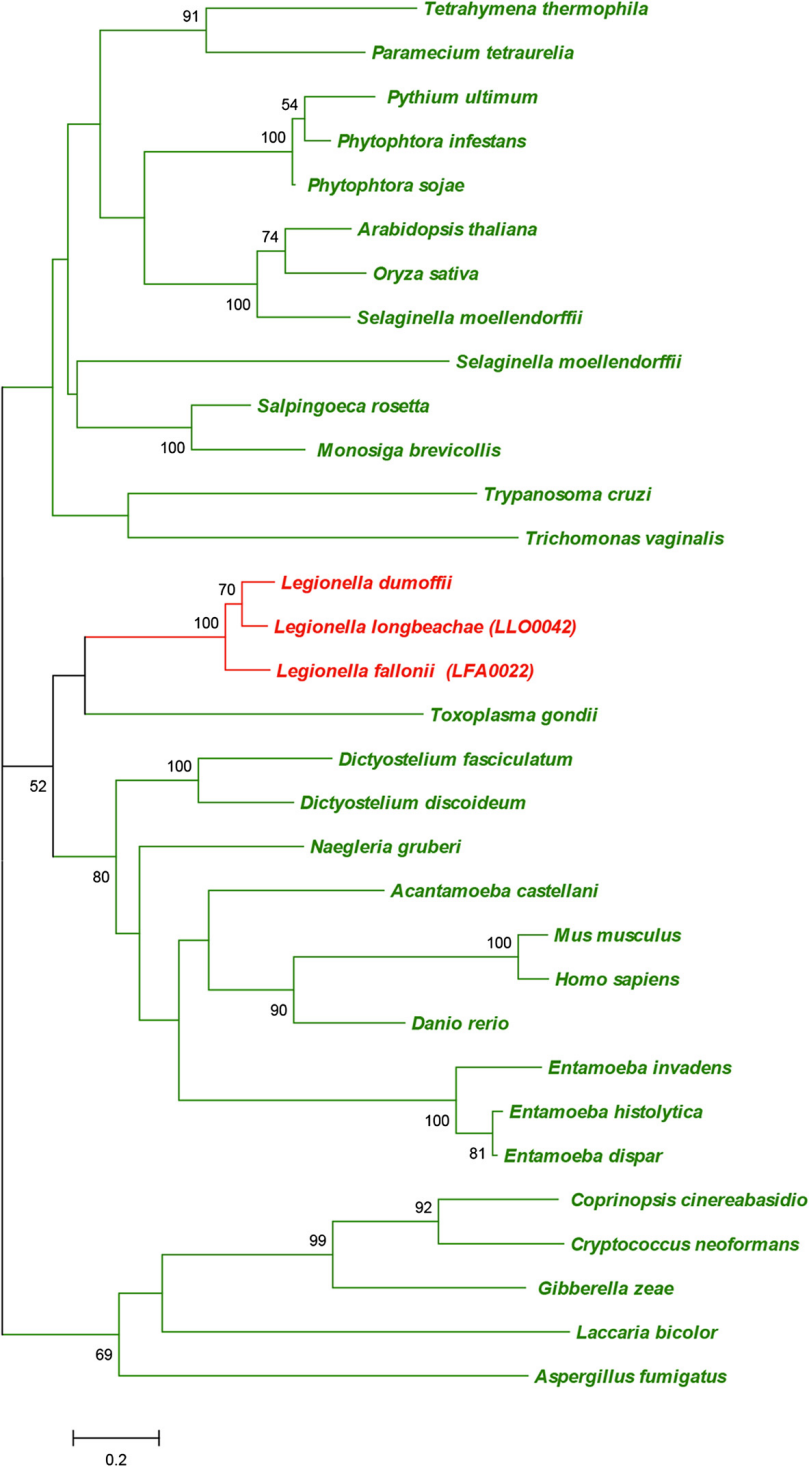
**Phylogenetic analysis shows the eukaryotic origin of the carboxypeptidase S28 family protein (Llo0042/Lfa0022).** The species belonging to bacteria and eukaryotes are shown in red and green, respectively. Numbers next to tree nodes correspond to bootstrap values. The bar at the bottom represents the estimated evolutionary distance.

Among these proteins 27 had not been described before and 15 were identified in the newly sequenced species. A clear case of horizontal gene transfer from eukaryotes is GamA (Lpp0489), a glucoamylase that allows *Legionella* to degrade glycogen during intracellular replication in *A. castellanii* [66]. In addition to already characterized proteins, we identified promising candidates for host-pathogen interactions in this study - for example, a *L. longbeachae* protein containing a tubulin-tyrosine ligase domain (Llo2200; Figure S9A in Additional file 14), a motif involved in addition of a carboxy-terminal tyrosine to α-tubulin as part of a tyrosination-detyrosination cycle that is present in most eukaryotic cells. This tyrosination process regulates the recruitment of microtubule-interacting proteins [67]. It is thus tempting to assume that *Legionella* is able to interfere with or to modulate the recruitment of microtubule-interacting proteins in the host. Another example is the serine carboxypeptidase S28 family protein (Llo0042/Lfa0022; Figure 7). These proteins have been identified exclusively in eukaryotes and are active at low pH, suggesting a function in the phagosome [68].

Taken together, each *Legionella* genome contains many different ELPs and proteins carrying eukaryotic domains that help *Legionella* to establish its intracellular niche. Some of these proteins are specific to one or other *Legionella* species but most are present in all of them, although these proteins are rarely real orthologues. This suggests that the acquisition of these proteins is important for *Legionella* to manipulate the host but that their horizontal acquisition has taken place on multiple occasions.

### Linking virulence properties and gene content

When using THP-1 cells as a model for infection of human macrophages, not all *Legionella* species were able to infect and replicate (Figure 1A). These results correlated with the epidemiology of legionellosis where only certain *Legionella* species are isolated from human disease. With the aim of identifying the genetic bases conferring these differences, we searched for genes that were present in the strains that cause disease but absent in the ones that had not been isolated from humans. This comparative analysis showed that *L. pneumophila*, *L. longbeachae* and *L. micdadei* share 40 genes that are not present in any of the other species. Among those we identified the *hyp* operon (*hypABFCDE* - *lpg2171-75*), necessary for hydrogenase activity in *E. coli* and the cyanobacterium *Synechocystis* [69]. *Legionella* has additional downstream genes encoding for hydrogenases that are unique to these three species. This region is flanked by tRNA genes in *L. micdadei* and *L. longbeachae*, suggesting its acquisition by horizontal gene transfer.

Furthermore, a gene encoding a truncated hemoglobin (*lpp2601*) of group I called trHbN was identified as specific to the human pathogenic strains. Truncated hemoglobins are a family of small oxygen-binding heme proteins [70] that are ubiquitous in plants and present in many pathogenic bacteria such as *Mycobacterium tuberculosis.* Mycbacteria missing trHbNs are severely impaired for nitric oxide detoxification [71], and the expression of this gene is required for *M. tuberculosis* during macrophage infection [72]. The proteins of *M. tuberculosis* and *L. pneumophila* share 30% identity and the important TrHbN residues are conserved in both, indicating a similar biochemical function. Furthermore, the *M. tuberculosis* trHbN shows 40% identity to its eukaryotic homologue in *Tetrahymena thermophila* and the *Legionella* protein 44% to the *T. thermophila* and 46% to the *Paramecium tetraurelia* protein. However, according to an in-depth phylogenetic analyses of truncated hemoglobins in prokaryotic and eukaryotic organisms, it seems that trHbNs are of prokaryotic origin and might have been transferred to eukaryotes [73]. Interestingly, the Lvh system is not part of the genes unique to *L. pneumophila*, *L. longbeachae* and *L. micdadei* as not all *L. pneumophila* strains contain it, but it is uniquely present only in these three species. Finally, of the more than 300 proteins described as translocated by the Dot/Icm secretion system, only two, CegC4 (*lpp2150*/*lpg2200*) and Lem25 (*lpp2487*/*lpg2422*), are exclusive to the three species found in human disease, but their function is not known yet.

Comparing *L. pneumophila* and *L. longbeachae*, the two species responsible for over 95% of human infections, to all other *Legionella* species, showed that 124 genes are specific to these human pathogenic *Legionella*. Among them are 38 substrates of the Dot/Icm secretion system, including RalF (*lpp1932*/*lpg1950*), SidJ (*lpp2094*/*lpg2155*), SidI (*lpp2572*/*lpg2504*), SdeC (*lpp2092*/*lpg2153*), SidE (*lpp2572*/*lpg2504*), SdcA (*lpp2578*/*lpg2510*) and CegC7 (*lpp0286*/*lpg0227*). In addition to the secreted substrates, iron availability seems to be important for the human pathogens as among the specific proteins several are related to iron scavenging or iron storage. These are homologues of PvcA and PvcB (*lpp0236-lpp0237*), the siderophore pyoverdine that is involved in virulence and biofilm formation in the cystic fibrosis pathogen *Pseudomonas aeuroginosa* [74]. In *Legionella* these genes are highly expressed in sessile cells, suggesting their involvement in sessile growth [75]. Furthermore, a bacterioferritin (*lpp2460*) that is present also in *L. micdadei* but highly divergent is specific for the human pathogenic *Legionella*. Bacterioferritin plays a role in iron storage and is involved in protecting cellular components from oxidative damage, thereby playing a role in oxidative stress relief [76,77]. Furthermore, a gene coding for a homologue of the *Yersinia pestis* plasminogen activator (*lpp2452*) that was shown to create transient plasmin activity [78] and the phospholipase C (*lpp1411*) implicated in host killing in a *G. mellonella* model [79] are specific to *L. pneumophila* and *L. longbeachae*.

## Conclusions

The first comprehensive analyses of five species of the genus *Legionella* and the comparison of the genomes of human disease-related strains with non-disease-related strains have provided new insights into the genomic specificities related to adaptation and host-pathogen interactions of this fascinating intracellular bacterium and have identified specific features of the major human pathogenic *Legionella*. Highly dynamic genomes that evolve through frequent horizontal gene transfer, mediated by many and diverse T4SSs and acquisition of different eukaryotic proteins and protein domains at multiple times and stages of their evolution that allow host subversion are a hallmark of this amoeba-associated bacterial genus. The major human-related *Legionella* species, *L. pneumophila* and *L. longbeachae*, contain a set of genes that seems to increase their successful infection of mammalian cells. The key to their success may be a better capacity to subvert host functions to establish a protective niche for intracellular replication due to a specific set of secreted effectors and a higher ability to acquire iron and to resist oxidative damage. The analysis of additional *Legionella* genomes and other intracellular pathogens may allow the future definition of the major common strategies used by intracellular pathogens to cause disease and to understand how environmental pathogens may evolve to become human pathogens.

## Materials and methods

### Bacterial strains and sequence accession numbers

The strains sequenced in this study were *L. hackeliae* strain ATCC35250 (EMBL accession number chromosome: PRJEB7321), *L. micdadei* ATCC 33218 (EMBL accession number chromosome: PRJEB7312) and *L. fallonii* strain LLAP-10 (ATCC700992; EMBL accession number chromosome: PRJEB7322) [25]. We obtained also the draft genome sequence of *L. micdadei* strain 02/42 (SRA accession number SRP047311), a clinical isolate from the Victorian Infectious Disease Research Laboratory (VIDRL). In addition, the genomes of *Legionella* species/strains that had been completely sequenced and published previously were included in the comparative analysis: *L. pneumophila* (strains Paris, Lens, Philadelphia, Corby, Lorraine and HL 0604 1035, Alcoy) [7,8,12,14] and *L. longbeachae* strain NSW150 [9].

### Sequencing and assembly

Strain *L. micdade*i 02/42 was sequenced using the Roche 454 GS-FLX platform, with Titanium chemistry and paired-end reads with an average insert size of 8.9 kb. The resultant reads, with an average length of 215 bp, were assembled using Newbler 2.5.3 (Roche/454) into three scaffolds with a total genome size of 3,266,670 bp (largest scaffold 3,261,115 bp) and an average read coverage of 26. *L. micdadeii* ATCC33218, *L. hackeliae* and *L. fallonii* sequences were determined using a Sanger/Illumina hybrid approach. For the Sanger approach sequencing reactions were performed using the ABI PRISM BigDye Terminator cycle sequencing ready reaction kit and a 3700 or a 3730 Xl Genetic Analyzer (Applied Biosystems, Saint Aubin, Ille de France, France). For *L. micdadei* ATCC33218, *L. hackeliae* and *L. fallonii*, 33,042, 33,042, and 36,240 sequences, respectively, from two libraries were determined. Assembly of the Sanger reads was done with the STADEN package in an iterative manner. We attempted to close remaining gaps with PCR products spanning repeats and regions recalcitrant to sequencing by testing several primer combinations for each gap. The final assemblies consisted of 36,084 reads and PCR products for *L. micdadei* ATCC33218, 33,085 for *L. hackeliae*, and 36,242 for *L. fallonii*. To finish the genome assembly each genome was in addition sequenced to a 60× coverage using an Illumina 2000 HiSeq sequencer and 36 bp reads. The Illumina reads and the programme Icorn [80] were used to correct the assembly and finish the genome.

### Annotation and genome comparison

The newly sequenced genomes of *L. fallonii*, *L. hackeliae* and *L. micdadei* were integrated into the MicroScope platform [81] to perform automatic and expert annotation of the genes, and comparative analysis with the already sequenced and integrated *L. pneumophila* strains. MicrosScope annotation is based on a number of integrated bioinformatic tools: Blast on UniProt and specialized genomic data, InterPro, COG, PRIAM, synteny group computation using the complete bacterial genomes available at NCBI RefSeq, and so on (for more details see [82]). Orthologous groups were established using the program PanOCT [83] with the following parameters: e-value 1e-5, percent identity ≥30, and length of match ≥65. The programs Easyfig and BRIG [84,85] were used for graphical representation of genome regions compared using BLAST. MAUVE [86] was used for aligning and comparing the *L. micdadei* genomes.

### *A. castellanii* and THP1 infection assays

In brief, cultures of *A. castellanii* were grown in PYG712 medium (2% proteose peptone, 0.1% yeast extract, 0.1 M glucose, 4 mM MgSO_4_, 0.4 M CaCl_2_, 0.1% sodium citrate dihydrate, 0.05 mM Fe(NH_4_)_2_(SO_4_)_2_ × 6H_2_O, 2.5 mM NaH_2_PO_3_, 2.5 mM K_2_HPO_3_) at 20°C for 3 days. Then amoeba were washed in infection buffer (PYG 712 medium without proteose peptone, glucose, and yeast extract) and adjusted to 10^5^ to 10^6^ cells/ml. Stationary phase *Legionella* grown on BCYE (Buffer Charcoal Yeast Extract) agar and diluted in water were mixed with *A. castellanii* at a multiplicity of infection MOI of 0.1. After allowing invasion for 1 h at 20°C the *A. castellanii* layer was washed twice with infection buffer (start point of time-course experiment). Intracellular multiplication was monitored using a 300 μl sample, which was centrifuged (14,000 rpm) and vortexed to break up amoeba. The number of colony forming units (CFU) of *Legionella* was determined by plating on BCYE agar. The infections were carried out in duplicates.

The human monocytic cell line THP-1 was maintained in RPMI 1640 medium GlutaMAX medium (Gibco, Invitrogen, Saint Aubin, Ille de France, France), supplemented with 10% fetal bovine serum (BIOWEST, France Nuaille, Maine et Loire , France), in 5% CO_2_ at 37°C. For THP-1 infection, cells were seeded into 24-well tissue culture trays (Falcon, BD lab ware, Altrincham, Manchester, United Kingdom, England) at a density of 1.5 × 10^5^ cells/well and pretreated with 10^−8^ M phorbol 12-myristate 13-acetate (PMA) for 72 h in 5% CO_2_ at 37°C to induce differentiation into macrophage-like adherent cells. Stationary phase *Legionella* were resuspended in RPMI 1640 serum free medium and added to THP-1 cell monolayers at an MOI of 10. After 1 h of incubation cells were treated with 100 μg ml^−1^ gentamycin for 1 h to kill extracellular bacteria. Infected cells were then washed with phosphate-buffered saline (PBS) before incubation with serum-free medium. At 24, 48 and 72 h THP-1 cells were lysed with 0.1% TritonX-100. The amount of *Legionella* was monitored by counting the number of CFU determined by plating on BCYE agar. The infections were carried out in triplicate.

### Cyclase translocation assay

The vector containing RalF-CyaA [29] was transformed into *L. micdadei*, *L. hackeliae and L. fallonii* and strain Paris wild type and its isogenic *ΔdotA::Km* mutant were used as positive and negative controls. Transformant strains were used to infect THP-1 cells previously plated at 1 × 10^5^ cells/well in 24-well tissue culture dishes and pre-treated with 10^−8^ M PMA. After 1 h and 30 minutes following infection cells were washed three times with cold PBS and lysed in 50 mM HCl, 0.1% Triton X-100. Lysates were boiled 5 minutes and neutralized with 0.5 M NaOH. We then added 95% cold ethanol and samples were spun for 5 minutes at maximum speed in a microcentrifuge. Supernatants were transferred in new 1.5 ml tubes and vacuum dried, and cAMP concentrations were measured using the cAMP Biotrak Enzyme immunoassay System (Amersham, United Kingdom, England). Each value was calculated as means of two independent infections ± standard deviations.

### Amoebae plate test

Samples of suspended amoeba were applied to BCYE agar plates as described previously [19]. Stationary-phase bacterial cultures (OD600 > 4.5) were adjusted to an identical OD600 (2.5), series of 10-fold dilutions in sterile H_2_O were prepared and 3 μl of each dilution were spotted onto CYE plates both with amoeba and without amoeba (control plates) and incubated for 3 to 5 days at 30°C or 37°C.

### Detection of new eukaryotic motifs in *Legionella* proteins

To better define the term 'eukaryotic motifs' we searched for the already known EMs in all proteins present in the Pfam database and calculated their occurrence in eukaryotic proteins or prokaryotic proteins. The previously described EMs in *Legionella* showed an occurrence of about 99% in eukaryotic proteins and only 1% in prokaryotic ones, with the ankyrin repeats being the less restricted to eukaryotic proteins (85%). The only exception is Sel-1 domains, which were considered as EMs. Sel-1 domains have now been shown to be highly present also in prokaryotes. However, since this domain is present in many substrates of the Dot/Icm system and it was shown to be implicated in host-pathogen interactions [87], it was taken into account. Based on the frequencies of the typical EMs present in *Legionella* we searched the Interpro database for all motifs that occur in eukaryotes at least to 85%. Using this criterion we obtained 8,329 motifs that can be considered as eukaryotic. These motifs were searched in all proteins predicted in the different *Legionella* genomes. This approach identified 10 eukaryotic motifs previously not described in *Legionella* proteins.

### Detection of genes transferred from eukaryotes to *Legionella*

To detect genes with putative eukaryotic origin we developed a pipeline based on several step filters. This pipeline was applied to one protein of each of the orthologous groups of the pan-proteome of the five studied species to avoid redundancy in the detection process with proteins of the same orthologous group. The first step consisted of discarding the protein families without significant similarity to eukaryotic sequences. This was achieved by a homology search using Blastp with an e-value cutoff of ≤10e^−4^ and a BLOSUM62 matrix with a representative protein of each group of orthologous families of the *Legionella* pan-genome against a database containing 83 genomes representative of all major eukaryotic phyla and certain viruses. In particular, members of *Amoebozoa* and other protist lineages that may be hosts for *Legionella* were included in this database. The results of the first filter led to the recovery of 2,669 proteins of the *Legionella* pan-genome with significant homology to eukaryotic sequences in the database. Then, among these 2,669 protein families those that have closer homologues in bacteria were discarded by searching for homologues against a database containing both eukaryotic and prokaryotic sequences using the same criteria. Only those that had at least a hit against a eukaryotic sequence among the first 25 hits were further selected. This step led to the selection of 465 protein families of the *Legionella* pan-genome representing ELP candidates. Finally, we carried out automatic phylogenetic reconstruction of these 465 proteins and their bacterial and eukaryotic homologues. The different steps of the pipeline were: (1) for each selected putative ELP the corresponding orthologues in other *Legionella* species analyzed where added if present; (2) each group of homologous sequences was aligned with MUSCLE [88]; (3) unambiguously aligned positions were automatically selected using the multiple alignment trimming program BMGE with low stringency parameters [89]; (4) preliminary maximum likelihood trees were obtained using FastTree [90]. We applied a strict filter to select only very likely ELPs. Then each of the 465 trees was manually inspected to select those where the *Legionella* sequences were branching within eukaryotes or were closer to eukaryotic sequences than to prokaryotic ones. This allowed identification of 40 *Legionella* proteins that aligned well with their eukaryotic homologues. For those having a sufficient number of eukaryotic homologues and a sufficient number of positions that could be selected after trimming, we proceeded to phylogenetic analysis by maximum likelihood using LG +4 gamma as the evolutionary model. Then, we selected those trees where the *Legionella* sequences were branching within eukaryotes or were closer to eukaryotic sequences than to prokaryotes. Finally, in order to verify the eventual existence of closer bacterial homologues or additional eukaryotic homologues from representatives not present in our local database, we performed a Blast on the non-redundant database at the NCBI. Alignments were obtained and trimmed, and trees reconstructed as described above.

### Phylogenetic reconstruction

For phylogenetic reconstruction two different data sets were created: one based on the concatenated alignment of 29 housekeeping genes (*lpp0086* (*uvrB*), *lpp0152* (*pgk*), *lpp0419* (*rpoA*), *lpp0467* (*ffh*), *lpp0575* (*serS*), *lpp0749* (*pros*), *lpp0791* (*glyA*), *lpp1020* (*lig*), *lpp1271* (*cysS*), *lpp1399* (*trpS*), *lpp1434* (*aspD*), *lpp1534* (*ruvB*), *lpp1738* (*nrdA*), *lpp1765* (*recA*), *lpp1830* (*tig*), *lpp1837* (*lepA*), *lpp2004* (*metK*), *lpp2006* (*dnaJ*), *lpp2013* (*argS*), *lpp2020* (*eno*), *lpp2662* (*ftsZ*), *lpp2698* (*uvrC*), *lpp2802* (*dnaX*), *lpp2877* (*recN*), *lpp2941* (*metG*), *lpp3002* (*rho*), *lpp3053* (*atpD*), *lpp3055* (*atpA*), *lpp3073* (*thdF*)) and another one based on all ortholgous genes among the studied species and *C. burnetii* as outgroup (816 genes). With these data sets the alignment of amino acids and the alignment of nucleotides based on the amino acid alignment were carried out. Individual genes/proteins were aligned with muscle and concatenated. The nucleotide alignments were cleaned using Gblocks [91]. Trees were constructed using both a distance method (neighbor-joining) implemented in the program MEGA [92] and a likelihood method using the software RaxML [93]. Bootstrap support was determined using 1,000 bootstrap replicates.

### Test for chitinase degradating activity

According to Vadake [40], Whatman filter paper strips were cut to 5 cm × 1 cm. These strips were immersed and air-dried in a solution of p-nitroacetanilide (5 g in 100 ml of ethanol 100%). The procedure was repeated three times to impregnate well the strips with p-nitroacetanilide. *L. fallonii* and *L. pneumophila* (used as negative control) were grown in liquid medium for 24 h and 2 ml of these cultures were transferred to a new sterile tube containing 2 ml of fresh liquid media and the diagnostic strips. These cultures were grown for 2 days at 30°C for *L. fallonii* and 37°C for *L. pneumophila*. After 2 days the development of yellow color on the strip indicated the presence of deacetylase in the corresponding bacterial culture.

### Cellulose detection assays

To visualize the production of cellulose, plates containing *Legionella* BCYE medium supplemented with calcofluor (5%; fluorescent brightener 28; Sigma-Aldrich, Oakville, Ontario, Canada) were prepared. Drops of 5 μl of liquid media containing *L. fallonii* grown for 72 h were spread on the plates and incubated at 30°C for 48 h. The same procedure was carried out for *L. pneumophila* at 37°C as negative control. After incubation plates were visualized under a UV light source.

## Additional files

Additional file 1: Figure S1. The ability of *L. micdadei* to replicate intracellularly in *A. castellanii* is dependent on the temperature. *L. pneumophila* strain Paris wild type (wt) and ∆*dotA* were used as positive and negative controls, respectively. Intracellular replication of *L. micdadei* at 30°C was determined by recording the number of colony-forming units (CFU) through plating on BCYE agar. Blue, wild-type *L. pneumophila* strain Paris; red, ∆*dotA*; orange, *L. micdadei*. Results are expressed as Log10 ratio CFU Tn/T0 and each point represents the mean ± standard deviation of two independent experiments. The error bars represent standard deviation, but some were too small to clearly appear in the figure. Additional file 2: Figure S2. The Dot/Icm-encoding genes are present in all *Legionella* species with variable homology. Graphical representation of Blastp comparisons of the genes encoding the Dot/Icm T4BSS in all strains/species used in this study. *L. pneumophila* strain Philadelphia genes were taken as query. Each color ring represents a species/strain, and each segment in the ring a different gene. The intensity of the color indicates the percentage of amino acid identity. The percentages of identity and their correlation with the color intensity is given in the left panel. Gene names are given in the outside circle. Gene names in red indicate 100% amino acid identity. Additional file 3: Figure S3. Genomic organization of the *L. fallonii* hopanoid encoding gene cluster. Blastx comparisons between clusters encoding genes for hopanoid biosynthesis in the species *Streptomyces coelicolor* and *L. fallonii* (LLAP10)*.* The gray color code represents the Blast matches; the darker the gray the better the blast match. Protein names and their predicted functions are indicated below. Additional file 4: Table S1. Putative mobile regions present in *L. fallonii* (LLAP10) containing a chloramphenicol acetyltransferase gene and a erythromycin esterase gene. Additional file 5: Figure S4.
*L. fallonii* shows chitinase degradation activity*.* Whatman paper strips dipped in a p-nitroacetanilide solution were introduced in liquid cultures of the species *L. hackeliae*, *L. fallonii* and *L. pneumophila* (used as negative control). After 2 days of growth the development of yellow color indicates the presence of chitin deacetylase activity in the bacterial culture. Additional file 6: Table S2. Putative prophage region in the genome of *L. micdadei* strain ATCC33218. Additional file 7: Figure S5. Genomic organization and comparison of the three conserved flagella gene clusters among the five *Legionella* species. Blastx comparison of these clusters in *L. pneumophila*, *L. longbeachae*, *L. hackeliae*, *L. fallonii* (LLAP10) and *L. micdadei*. **(A)** Flagellar region 1. **(B)** Flagellar region 2. **(C)** Flagellar region 3. The gray color code represents the Blast matches; the darker the gray the better the blast match. Additional file 8: Figure S6. The *L. pneumophila* strain Paris plasmid is quasi-identical to the *L. hackeliae* plasmid. Chromosomal organization and Blastn comparison shows 100% nucleotide identity except for the insertion of two transposase-encoding genes in strain Paris. The gray color code represents the Blast matches; the darker the gray the better the blast match. Additional file 9: Figure S7. Comparison of F-type IV secretion systems present in the different *Legionella* species*.* Genomic organization and Blastx comparison between clusters encoding the F-type T4ASS, localized in the chromosome and on plasmids. Presence of the gene *lvrC* is indicated by a red dot. The gray color code represents the Blast matches; the darker the gray the better the blast match. Numbers indicate the chromosomal location. Additional file 10: Figure S8. The Lvh-encoding region of the two sequenced *L. micdadei* strains is highly divergent. The Lvh T4ASS-encoding genes are highly conserved among all *Legionella* Blastx comparisons between the region encoding the Lvh system and the flanking genes in the two *L. micdadei* strains compared in this study. Blast matches are represented with gray lines that are darker as the blast match improves. Additional file 11: Table S3. List of confirmed substrates of the Dot/Icm secretion system. Additional file 12: Table S4. List of eukaryotic organisms and viruses whose genomes have been selected for the construction of the eukaryotic genome database for Blast searches. Additional file 13: Table S5 Orthologous genes encoding eukaryotic motifs in five *Legionella* species. Additional file 14: Figure S9. Phylogenetic reconstruction for *Legionella* proteins that were probably transferred horizontally from eukaryotic organisms. Groups of homologous sequences were aligned with MUSCLE [89], unambiguously aligned positions were automatically selected using the multiple alignment trimming program BMGE [84] with low stringency parameters. After trimming, phylogenetic analysis was done using maximum likelihood.

## Abbreviations

bp: base pair
BCYE: Buffer Charcoal Yeast Extract
CFU: colony forming units
ELP: eukaryotic-like protein
EM: eukaryotic motif
MOI: multiplicity of infection
PBS: phosphate-buffered saline
PMA: phorbol 12-myristate 13-acetate
SNP: single-nucleotide polymorphism
T4SS: type IV secretion system
