# Supplementary figures and images for: Comparative analyses of Legionella species identifies genetic features of strains causing Legionnaires’ disease
Source: Genome Biol. 2014 Nov 3;15(11):505. doi: 10.1186/s13059-014-0505-0 (PMC4256840; doi:10.1186/s13059-014-0505-0)

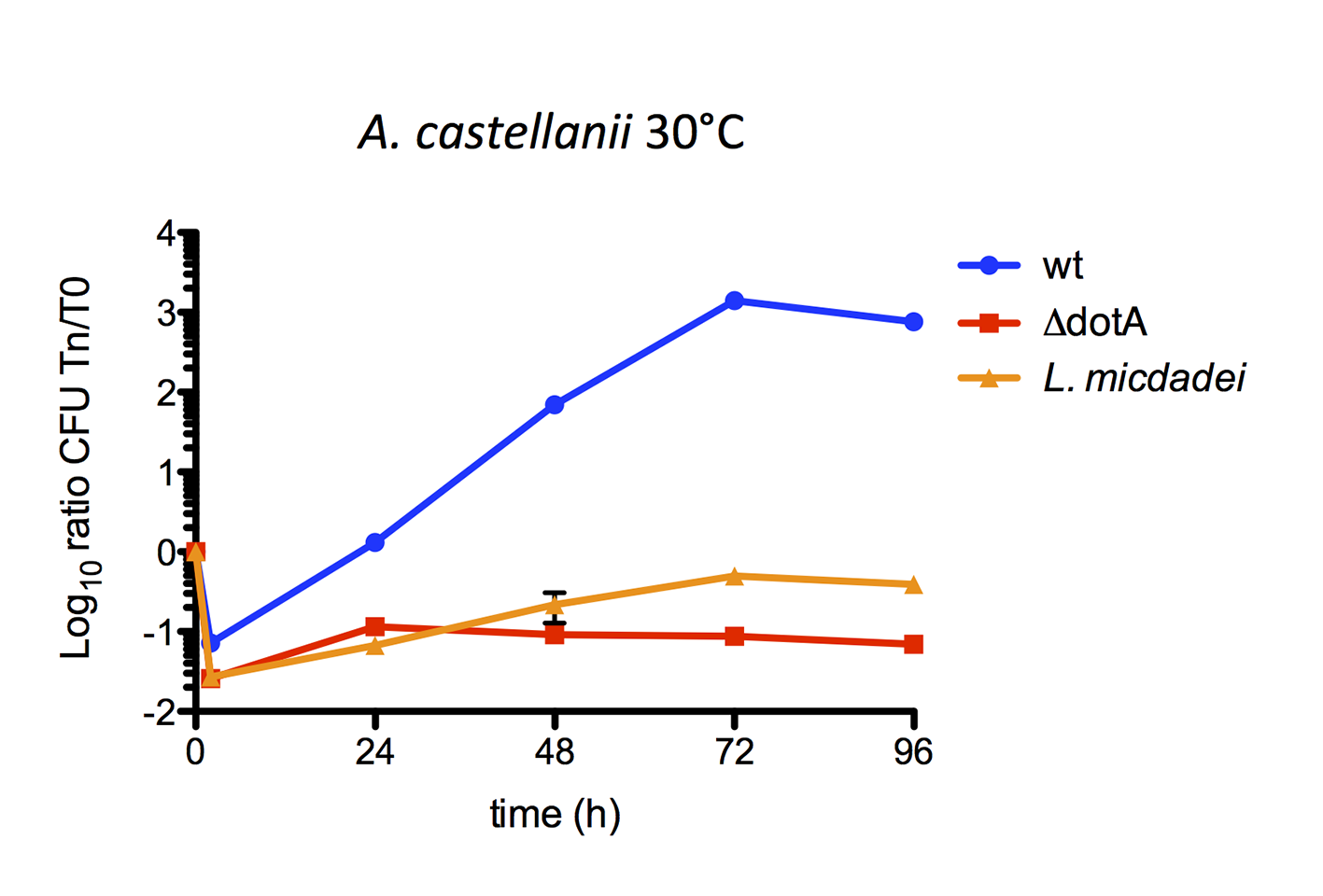

Supplement: Additional file 1: Figure S1. — The ability of L. micdadei to replicate intracellularly in A. castellanii is dependent on the temperature. L. pneumophila strain Paris wild type (wt) and ∆dotA were used as positive and negative controls, respectively. Intracellular replication of L. micdadei at 30°C was determined by recording the number of colony-forming units (CFU) through plating on BCYE agar. Blue, wild-type L. pneumophila strain Paris; red, ∆dotA; orange, L. micdadei. Results are expressed as Log10 ratio CFU Tn/T0 and each point represents the mean ± standard deviation of two independent experiments. The error bars represent standard deviation, but some were too small to clearly appear in the figure. [file 13059_2014_505_MOESM1_ESM.tiff]

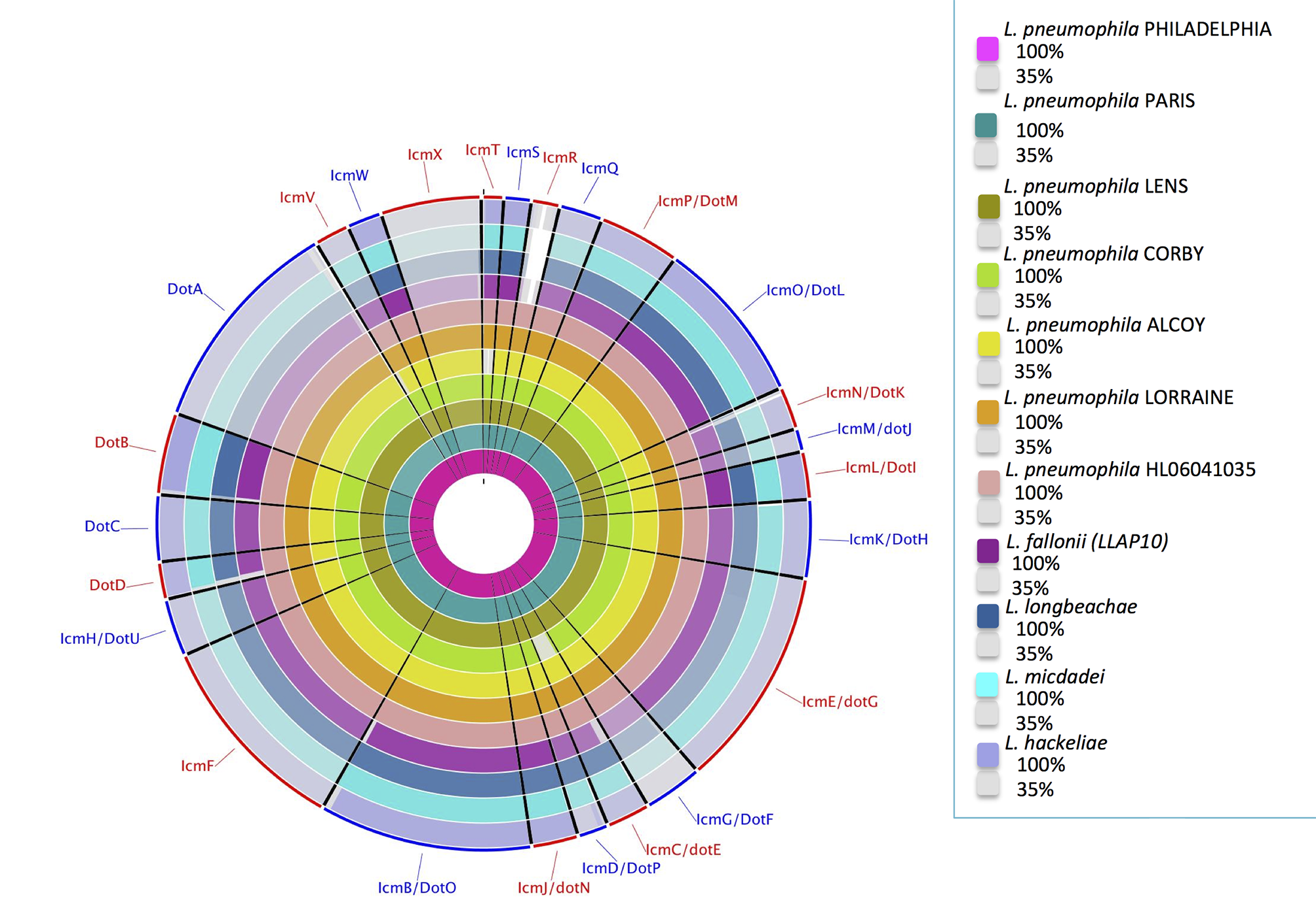

Supplement: Additional file 2: Figure S2. — The Dot/Icm-encoding genes are present in all Legionella species with variable homology. Graphical representation of Blastp comparisons of the genes encoding the Dot/Icm T4BSS in all strains/species used in this study. L. pneumophila strain Philadelphia genes were taken as query. Each color ring represents a species/strain, and each segment in the ring a different gene. The intensity of the color indicates the percentage of amino acid identity. The percentages of identity and their correlation with the color intensity is given in the left panel. Gene names are given in the outside circle. Gene names in red indicate 100% amino acid identity. [file 13059_2014_505_MOESM2_ESM.tiff]

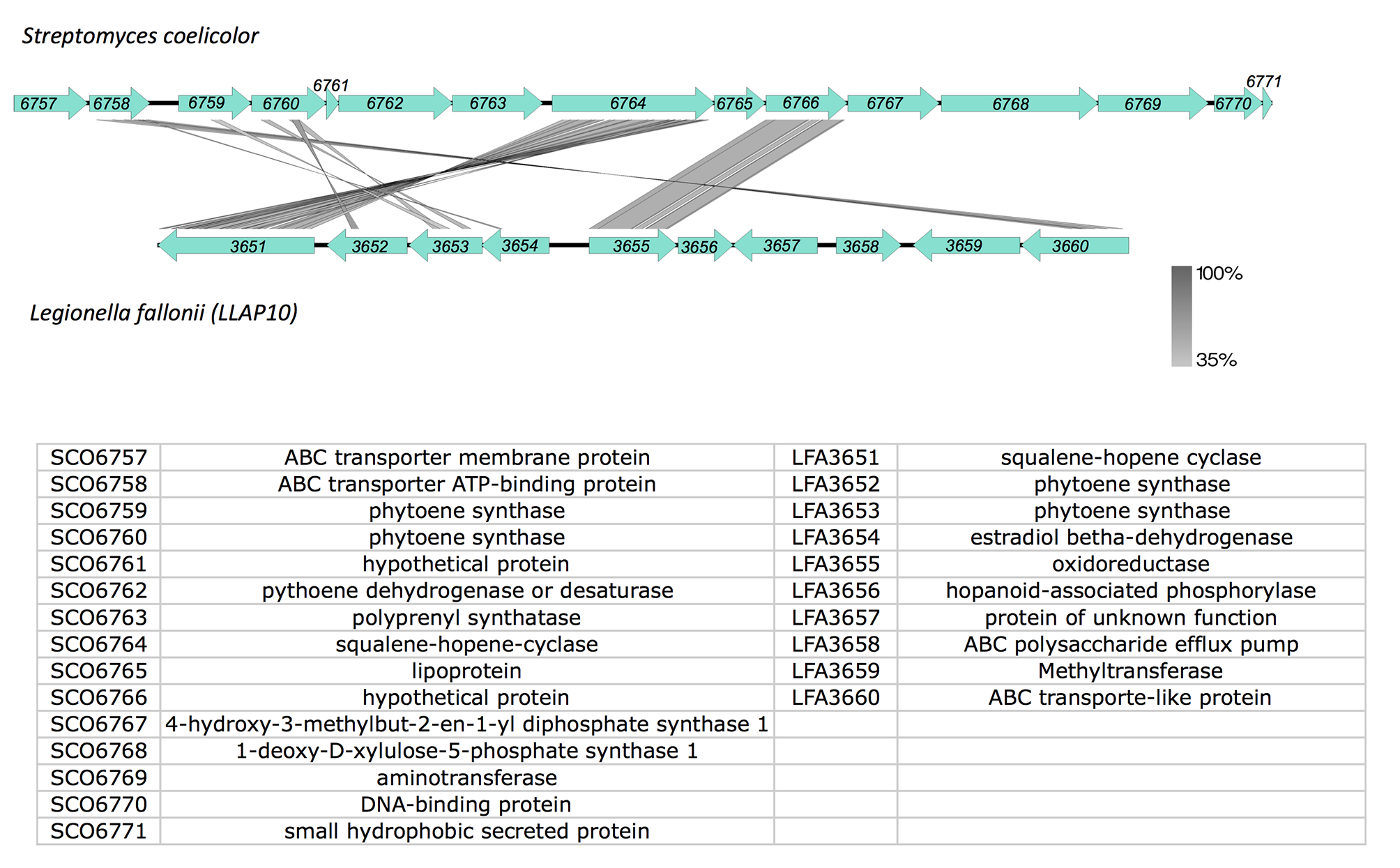

Supplement: Additional file 3: Figure S3. — Genomic organization of the L. fallonii hopanoid encoding gene cluster. Blastx comparisons between clusters encoding genes for hopanoid biosynthesis in the species Streptomyces coelicolor and L. fallonii (LLAP10). The gray color code represents the Blast matches; the darker the gray the better the blast match. Protein names and their predicted functions are indicated below. [file 13059_2014_505_MOESM3_ESM.tiff]

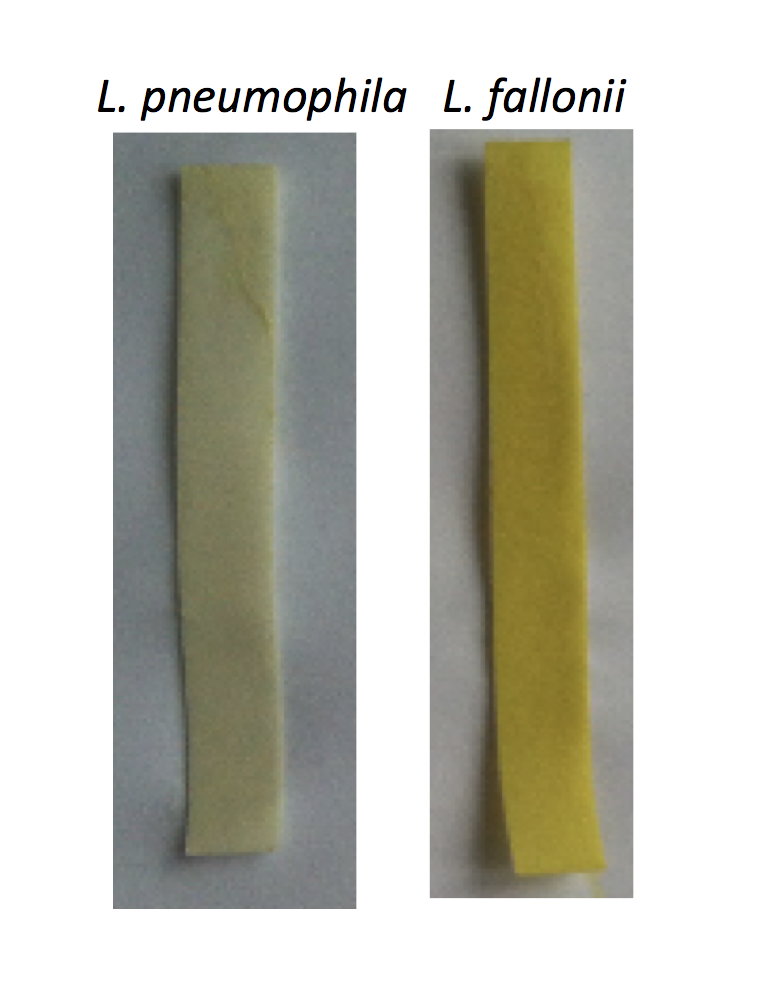

Supplement: Additional file 5: Figure S4. — L. fallonii shows chitinase degradation activity. Whatman paper strips dipped in a p-nitroacetanilide solution were introduced in liquid cultures of the species L. hackeliae, L. fallonii and L. pneumophila (used as negative control). After 2 days of growth the development of yellow color indicates the presence of chitin deacetylase activity in the bacterial culture. [file 13059_2014_505_MOESM5_ESM.tiff]

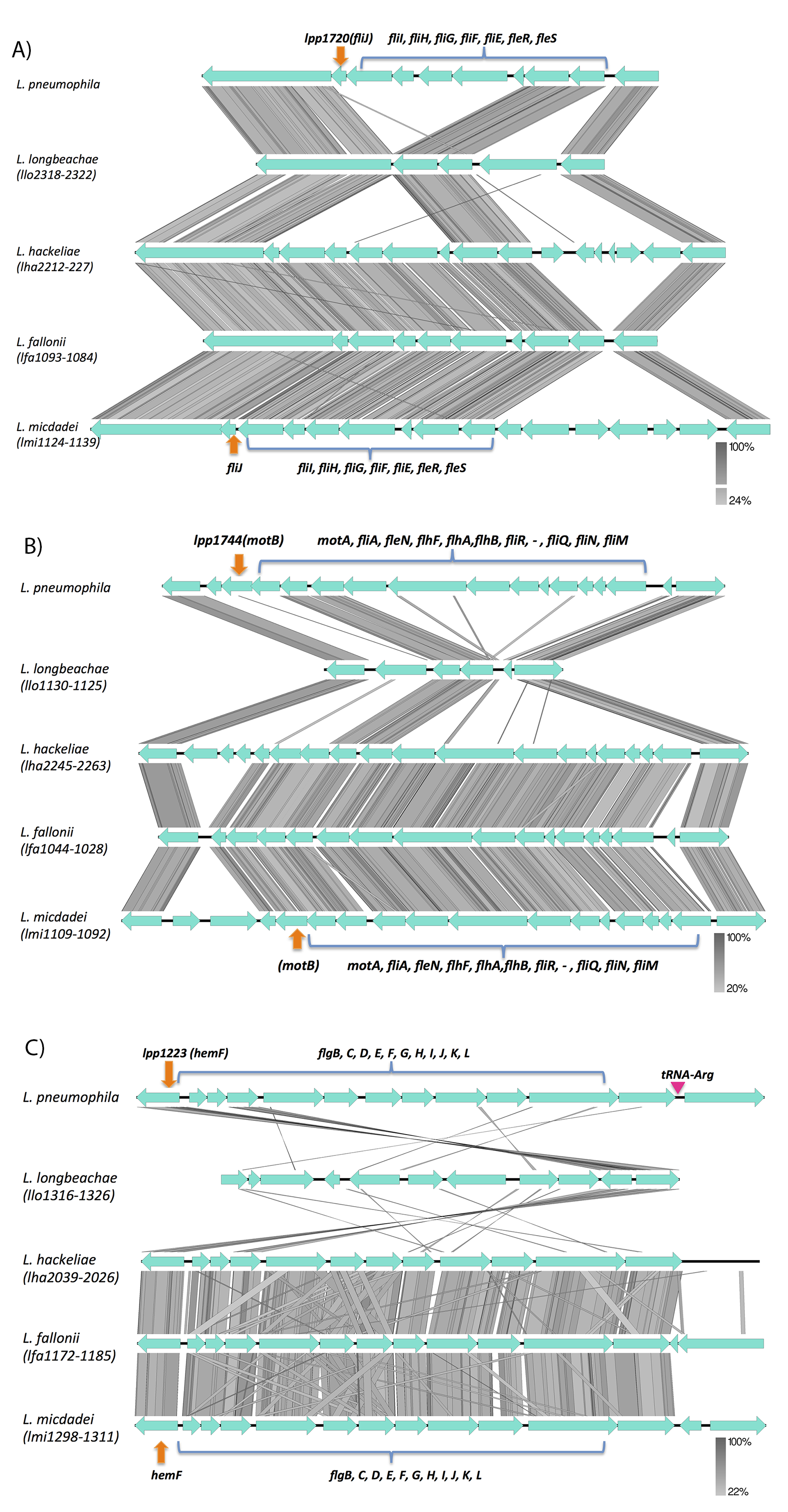

Supplement: Additional file 7: Figure S5. — Genomic organization and comparison of the three conserved flagella gene clusters among the five Legionella species. Blastx comparison of these clusters in L. pneumophila, L. longbeachae, L. hackeliae, L. fallonii (LLAP10) and L. micdadei. (A) Flagellar region 1. (B) Flagellar region 2. (C) Flagellar region 3. The gray color code represents the Blast matches; the darker the gray the better the blast match. [file 13059_2014_505_MOESM7_ESM.tiff]

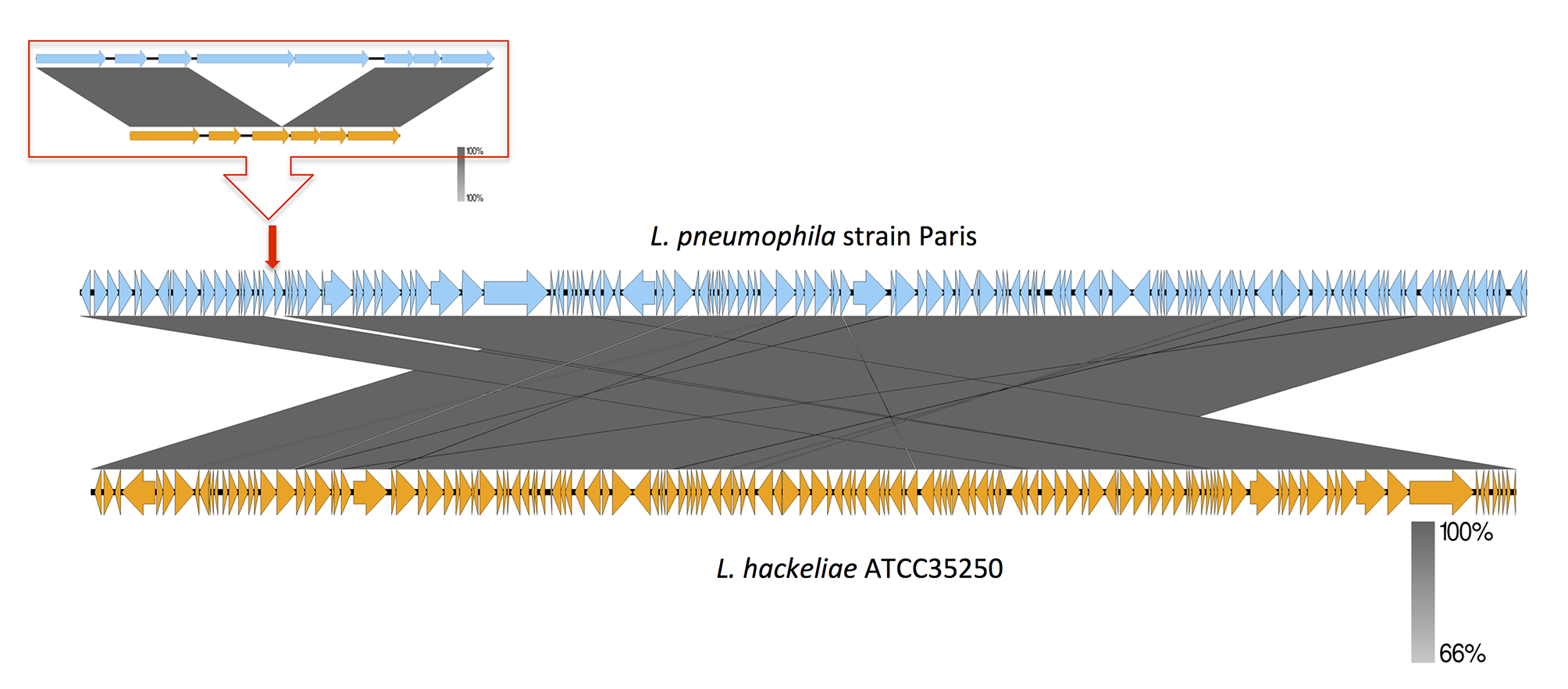

Supplement: Additional file 8: Figure S6. — The L. pneumophila strain Paris plasmid is quasi-identical to the L. hackeliae plasmid. Chromosomal organization and Blastn comparison shows 100% nucleotide identity except for the insertion of two transposase-encoding genes in strain Paris. The gray color code represents the Blast matches; the darker the gray the better the blast match. [file 13059_2014_505_MOESM8_ESM.tiff]

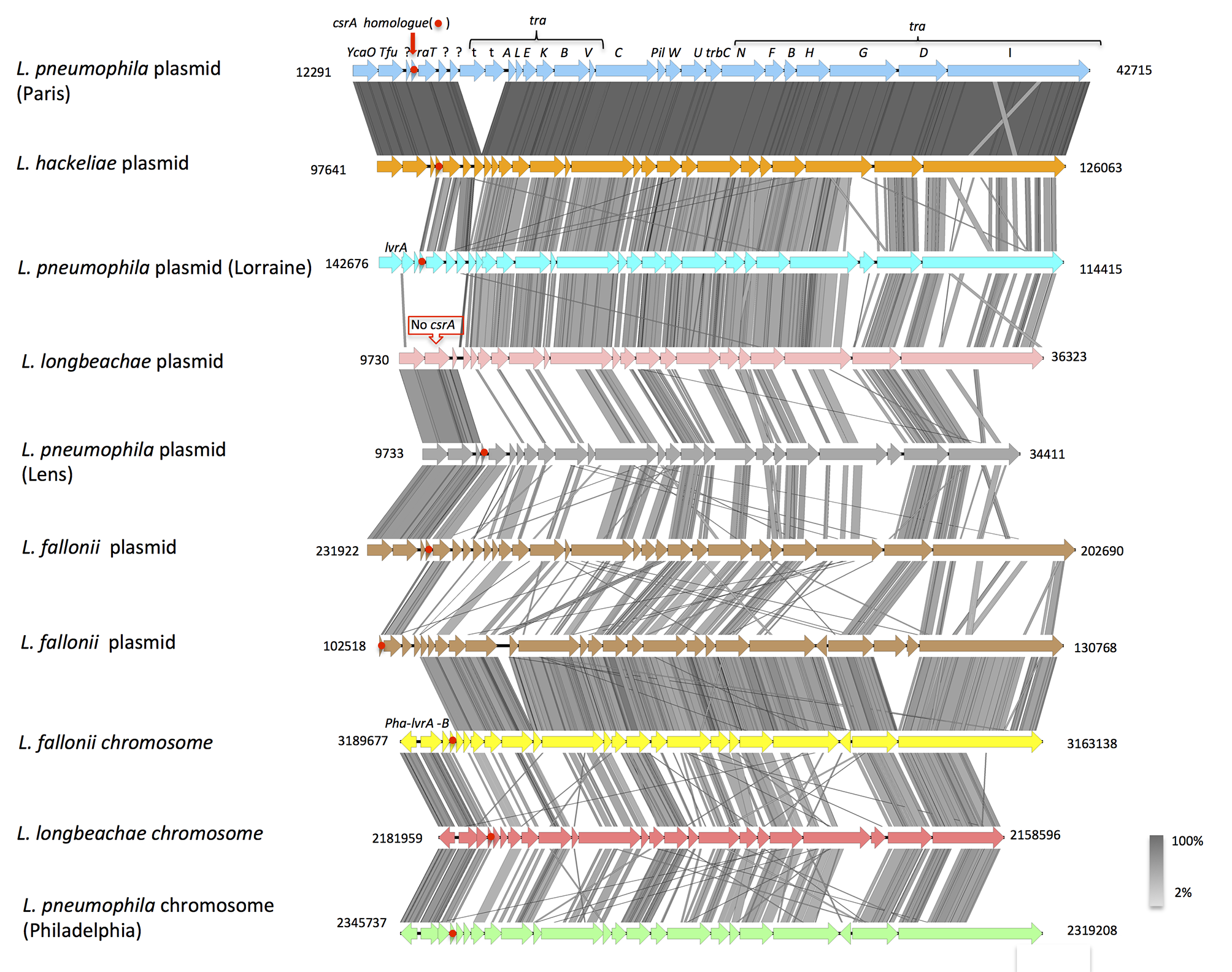

Supplement: Additional file 9: Figure S7. — Comparison of F-type IV secretion systems present in the different Legionella species. Genomic organization and Blastx comparison between clusters encoding the F-type T4ASS, localized in the chromosome and on plasmids. Presence of the gene lvrC is indicated by a red dot. The gray color code represents the Blast matches; the darker the gray the better the blast match. Numbers indicate the chromosomal location. [file 13059_2014_505_MOESM9_ESM.tiff]

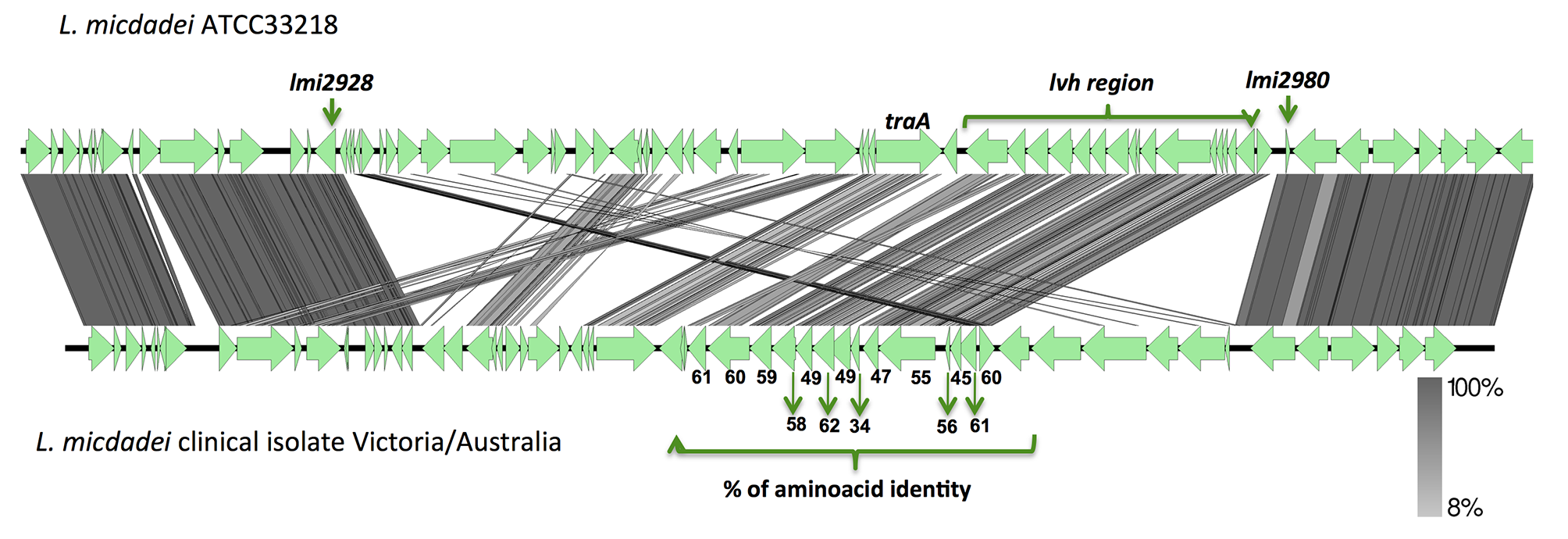

Supplement: Additional file 10: Figure S8. — The Lvh-encoding region of the two sequenced L. micdadei strains is highly divergent. The Lvh T4ASS-encoding genes are highly conserved among all Legionella Blastx comparisons between the region encoding the Lvh system and the flanking genes in the two L. micdadei strains compared in this study. Blast matches are represented with gray lines that are darker as the blast match improves. [file 13059_2014_505_MOESM10_ESM.tiff]

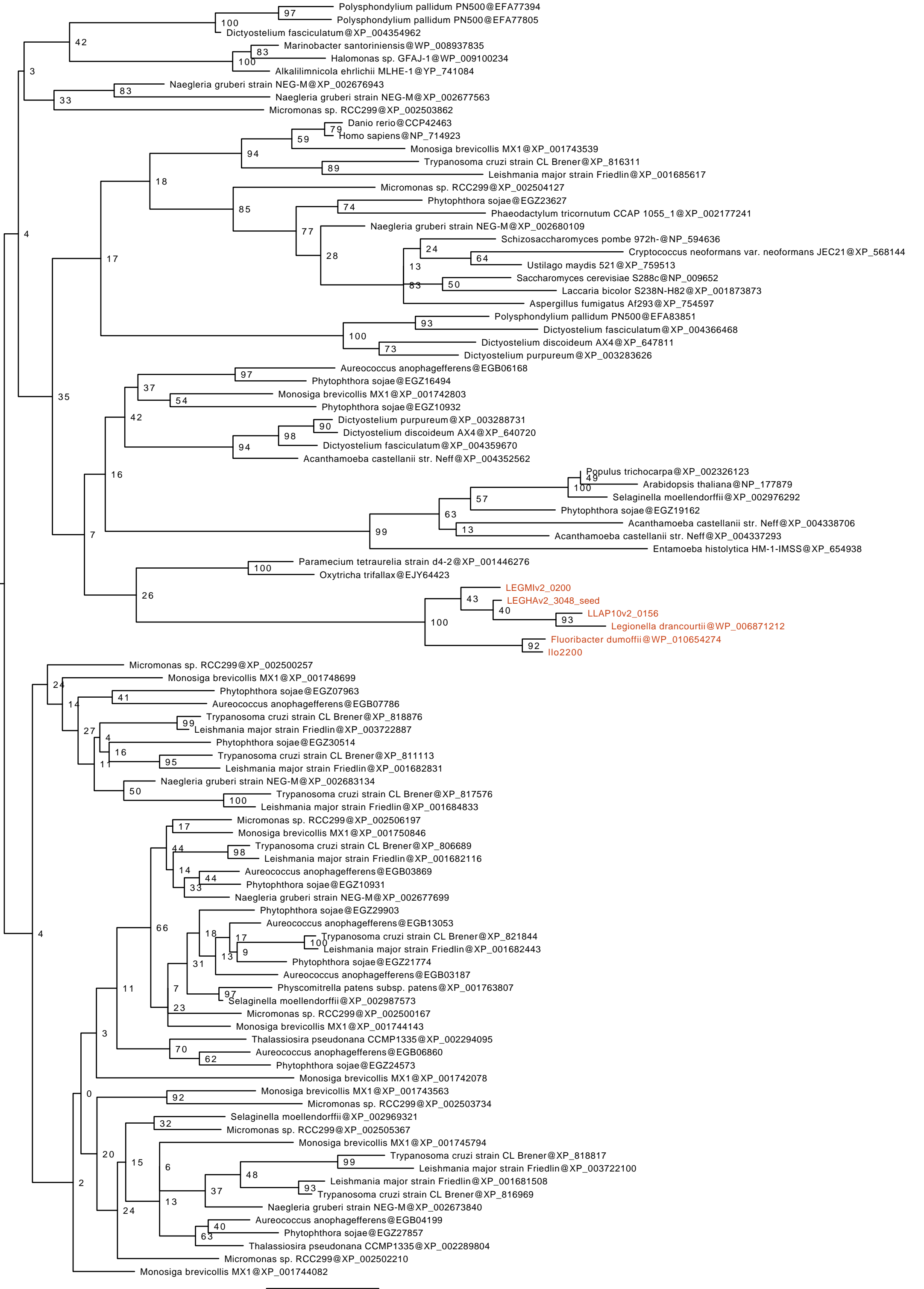

Supplement: Additional file 14: Figure S9. — Phylogenetic reconstruction for Legionella proteins that were probably transferred horizontally from eukaryotic organisms. Groups of homologous sequences were aligned with MUSCLE [89], unambiguously aligned positions were automatically selected using the multiple alignment trimming program BMGE [84] with low stringency parameters. After trimming, phylogenetic analysis was done using maximum likelihood. [file 13059_2014_505_MOESM14_ESM.zip › 1086350395137407_add14.pdf]

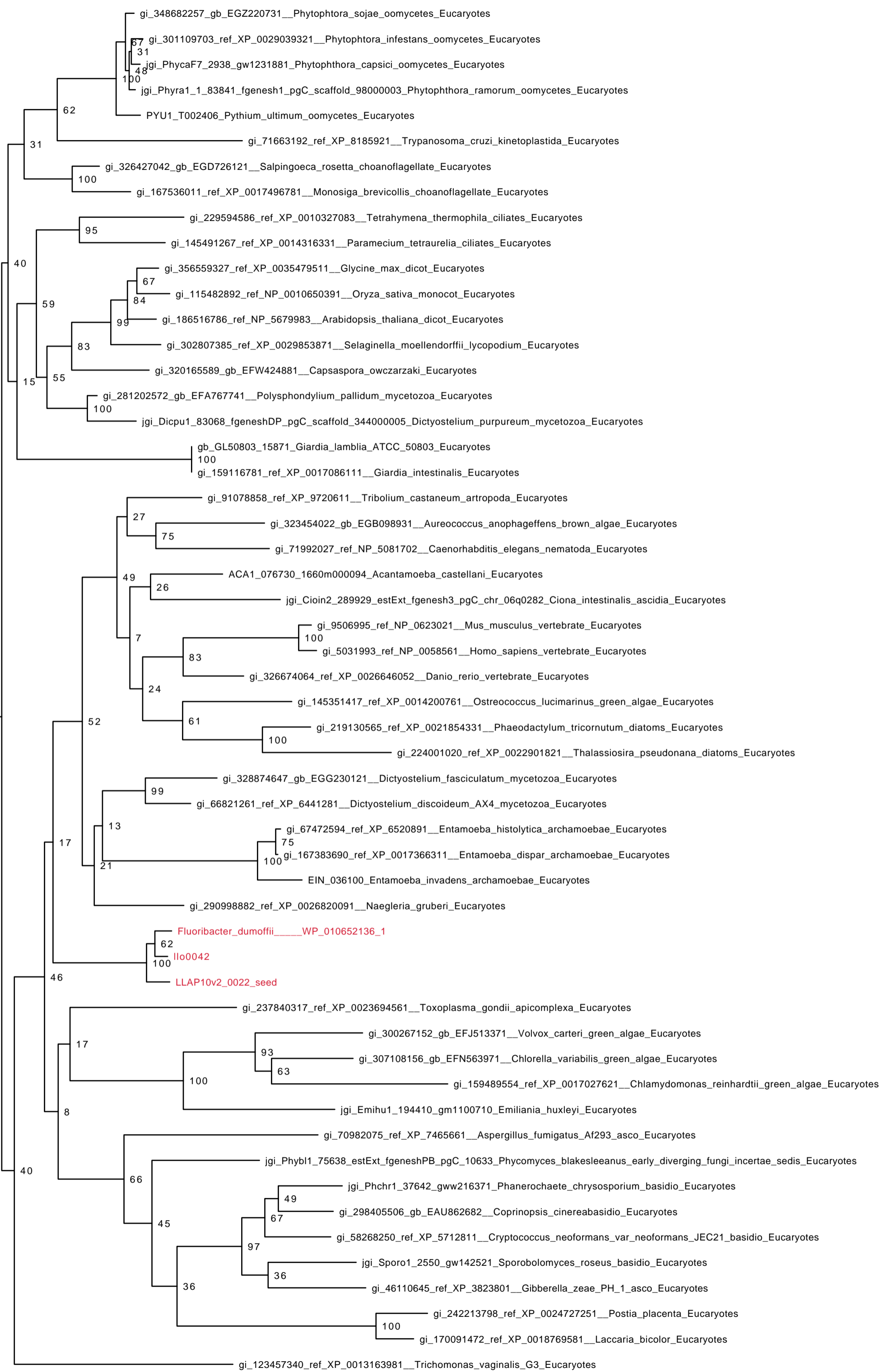

0.4

Supplement: Additional file 14: Figure S9. — Phylogenetic reconstruction for Legionella proteins that were probably transferred horizontally from eukaryotic organisms. Groups of homologous sequences were aligned with MUSCLE [89], unambiguously aligned positions were automatically selected using the multiple alignment trimming program BMGE [84] with low stringency parameters. After trimming, phylogenetic analysis was done using maximum likelihood. [file 13059_2014_505_MOESM14_ESM.zip › 1086350395137407_add15.pdf]

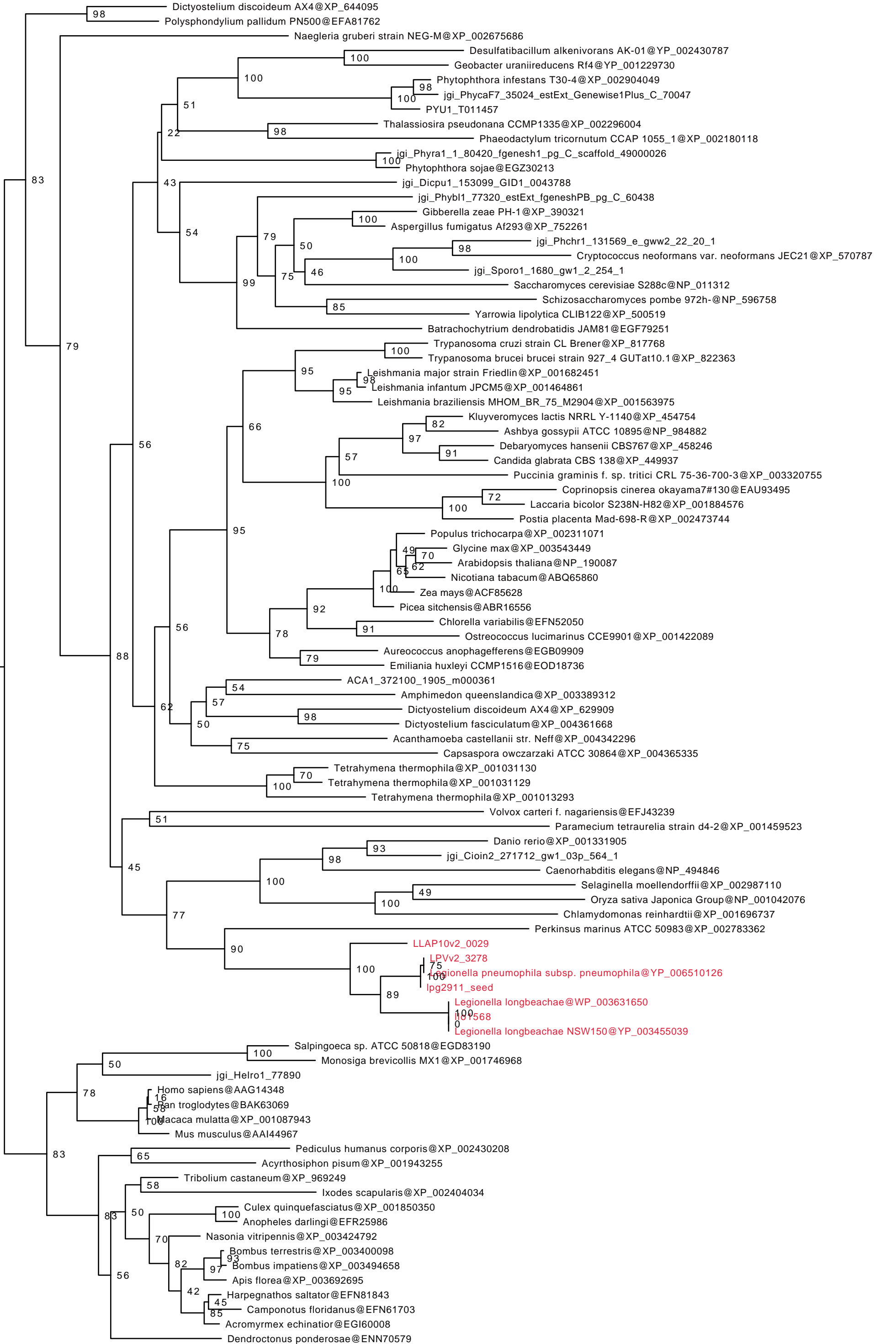

0.3

Supplement: Additional file 14: Figure S9. — Phylogenetic reconstruction for Legionella proteins that were probably transferred horizontally from eukaryotic organisms. Groups of homologous sequences were aligned with MUSCLE [89], unambiguously aligned positions were automatically selected using the multiple alignment trimming program BMGE [84] with low stringency parameters. After trimming, phylogenetic analysis was done using maximum likelihood. [file 13059_2014_505_MOESM14_ESM.zip › 1086350395137407_add16.pdf]
