# Supplementary material for: Comparative analyses of Legionella species identifies genetic features of strains causing Legionnaires’ disease
Source: Genome Biol. 2014 Nov 3;15(11):505. doi: 10.1186/s13059-014-0505-0 (PMC4256840; doi:10.1186/s13059-014-0505-0)
Supplement: Additional file 4: Table S1. — Putative mobile regions present in L. fallonii (LLAP10) containing a chloramphenicol acetyltransferase gene and a erythromycin esterase gene. [file 13059_2014_505_MOESM4_ESM.docx]

**Table S1.** Putative mobile regions present in *L. fallonii* (LLAP10) containing a chloramphenicol acetyltransferase gene and a erythromycin esterase gene.

| ***Label*** | **Gene** | **Product** |
| --- | --- | --- |
| ***lfa0203*** | ***int*** | Integrase |
| ***lfa0204*** |  | Putative protein with box helicase and with type III restriction enzyme motif |
| ***lfa0205*** |  | protein of unknown function |
| ***lfa0206*** | ***alpA*** | Prophage CP4-57 regulatory protein AlpA |
| ***lfa0207*** |  | conserved protein of unknown function |
| ***lfa0208*** |  | conserved protein of unknown function |
| ***lfa0209*** |  | conserved protein of unknown function |
| ***lfa0210*** |  | Death-on-curing family protein |
| ***lfa0211*** |  | CiaB protein |
| ***lfa0212*** |  | exported protein of unknown function [Pentapeptide repeat] |
| ***lfa0213*** |  | exported protein of unknown function [Pentapeptide repeat] |
| ***lfa0214*** |  | traM protein (fragment) |
| ***lfa0215*** |  | conserved protein of unknown function |
| ***lfa0216*** |  | conserved protein of unknown function [ATPase, AAA-4] |
| ***lfa0217*** |  | protein of unknown function |
| ***lfa0218*** |  | putative Transcriptional regulator, TetR family |
| ***lfa0219*** |  | Protochlorophyllide reductase |
| ***lfa0220*** |  | protein of unknown function |
| ***lfa0221*** |  | protein of unknown function |
| ***lfa0222*** |  | conserved protein of unknown function |
| ***lfa0223*** |  | conserved protein of unknown function |
| ***lfa0224*** |  | conserved protein of unknown function |
| ***lfa0225*** |  | conserved protein of unknown function |
| ***lfa0226*** |  | conserved protein of unknown function[macro domain-like] |
| ***lfa0227*** |  | protein of unknown function |
| ***lfa0228*** |  | conserved protein of unknown function [Fic-domain] |
| ***lfa0229*** |  | protein of unknown function |
| ***lfa0230*** |  | protein of unknown function |
| ***lfa0231*** | ***plaB*** | Phospholipase |
| ***lfa0233*** |  | membrane protein of unknown function |
| ***lfa0234*** |  | conserved exported protein of unknown function |
| ***lfa0235*** |  | protein of unknown function |
| ***lfa0236*** |  | conserved protein of unknown function |
| ***lfa0237*** | ***tag*** | DNA-3-methyladenine glycosylase |
| ***lfa0238*** |  | putative arsenate reductase |
| ***lfa0239*** |  | conserved exported protein of unknown function [Glycoside hydrolase domain] |
| ***lfa0240*** |  | protein of unknown function |
| ***lfa0241*** |  | putative Uridine kinase |
| ***lfa0243*** |  | putative Bacterial extracellular solute-binding family protein |
| ***lfa0244*** |  | putative protein translocase subunit SecA [5 coiled-coil domains] |
| ***lfa0246*** |  | protein of unknown function |
| ***lfa0248*** |  | Bacterial extracellular solute-binding protein |
| ***lfa0249*** |  | Glutamine amidotransferase class-I domain protein |
| ***lfa0250*** |  | Kynureninase |
| ***lfa0251*** |  | putative HAD-superfamily hydrolase subfamily IA, variant 3 |
| ***lfa0252*** |  | 67 kDa myosin-cross-reactive antigen family protein |
| ***lfa0253*** | ***ldcC*** | Lysine decarboxylase, constitutive |
| ***lfa0254*** |  | protein of unknown function [coiled-coil domain] |
| ***lfa0255*** |  | protein of unknown function |
| ***lfa0256*** |  | exported protein of unknown function |
| ***lfa0257*** |  | putative FAD/FMN-containing dehydrogenases |
| ***lfa0258*** |  | heme oxygenase |
| ***lfa0259*** |  | Spermidine synthase-like protein |
| ***lfa0260*** |  | membrane protein of unknown function |
| ***lfa0261*** |  | Copper-exporting ATPase |
| ***lfa0262*** | ***fur*** | Fe2 /Zn2 uptake regulation protein |
| ***lfa0263*** |  | protein of unknown function [8 coiled-coil domains] |
| ***lfa0264*** |  | Serine/threonine-protein kinase |
| ***lfa0265*** | ***ung*** | Uracil-DNA glycosylase |
| ***lfa0266*** |  | Beta-lactamase |
| ***lfa0267*** |  | protein of unknown function [coiled-coil domain] |
| ***lfa0268*** |  | protein of unknown function |
| ***lfa0269**** | ***catQ*** | **Chloramphenicol acetyltransferase** |
| ***lfa0270*** |  | putative short-chain dehydrogenase |
| ***lfa0272*** |  | exported protein of unknown function |
| ***lfa0273*** |  | protein of unknown function |
| ***lfa0274*** |  | Adenylyltransferase and sulfurtransferase MOCS3 [Includes: Adenylyltransferase MOCS3 ; Sulfurtransferase MOCS3] |
| ***lfa0275*** |  | putative sulfur carrier protein AF_0552 |
| ***lfa0276*** |  | protein of unknown function [TfuA domain] |
| ***lfa0277*** |  | putative OmpA-like transmembrane domain |
| ***lfa0278*** |  | conserved protein of unknown function [YcaO-like] |
| ***lfa0279*** |  | protein of unknown function [YcaO-like] |
| ***lfa0280*** |  | protein of unknown function [TfuA-like domain] |
| ***lfa0281*** |  | protein of unknown function |
| ***lfa0282*** |  | Sulfotransferase |
| ***lfa0283*** |  | protein of unknown function |
| ***lfa0284*** |  | protein of unknown function [FAD/NAD(P)-binding domain] |
| ***lfa0285*** |  | protein of unknown function |
| ***lfa0286*** |  | Xenobiotic-transporting ATPase |
| ***lfa0287*** |  | Xenobiotic-transporting ATPase |
| ***lfa0288*** |  | conserved exported protein of unknown function [amidase domain] |
| ***lfa0289*** |  | protein of unknown function [coiled-coil domain] |
| ***lfa0290*** |  | conserved protein of unknown function [coiled-coil domain] |
| ***lfa0291*** |  | Metal dependent phosphohydrolase [GAF domain] |
| ***lfa0292*** |  | protein of unknown function [F-BOX/LEUCINE RICH REPEAT PROTEIN] |
| ***lfa1754*** |  | putative 2-isopropylmalate synthase |
| ***lfa1756*** |  | protein of unknown function |
| ***lfa1758*** | ***fadE*** | Acyl-coenzyme A dehydrogenase |
| ***lfa1760*** |  | conserved protein of unknown function |
| ***lfa1761*** |  | conserved membrane protein of unknown function |
| ***lfa1762*** | ***ugpC*** | sn-glycerol-3-phosphate import ATP-binding protein UgpC |
| ***lfa1763*** | ***ugpE*** | sn-glycerol-3-phosphate transport system permease protein ugpE |
| ***lfa1764*** | ***ugpA*** | sn-glycerol-3-phosphate transport system permease protein ugpA |
| ***lfa1765*** |  | conserved protein of unknown function |
| ***lfa1766*** |  | conserved protein of unknown function |
| ***lfa1767*** | ***clcA*** | H(+)/Cl(-) exchange transporter ClcA |
| ***lfa1768*** | ***trpE(G*** | Anthranilate synthase [Includes: Glutamine amidotransferase] |
| ***lfa1769*** | ***gatC*** | Aspartyl/glutamyl-tRNA(Asn/Gln) amidotransferase subunit C |
| ***lfa1770*** | ***gatA*** | Glutamyl-tRNA(Gln) amidotransferase subunit A |
| ***lfa1771*** | ***gatB*** | Aspartyl/glutamyl-tRNA(Asn/Gln) amidotransferase subunit B |
| ***lfa1772*** |  | exported protein of unknown function |
| ***lfamisc_RNA_7*** | | RNaseP_bact_a |
| ***lfa1773*** | ***fis*** | DNA-binding protein fis |
| ***lfa1774*** |  | protein of unknown function |
| ***lfa1775*** |  | exported protein of unknown function |
| ***lfa1776*** | ***iscA*** | Iron-binding protein iscA |
| ***lfa1777*** |  | NifU family iron binding protein, [Fe-S] cluster formation/repair protein IscU, HesB |
| ***lfa1778*** | ***iscS*** | Cysteine desulfurase |
| ***lfa1779*** | ***yfhQ*** | putative methyltransferase |
| ***lfa1780*** | ***suhB*** | Inositol-1-monophosphatase |
| ***lfa1781*** |  | protein of unknown function [ankyrin repeat] |
| ***lfa1782*** |  | protein of unknown function |
| ***lfa1783*** |  | Signal peptide peptidase |
| ***lfa1784*** | ***clpB*** | Chaperone protein ClpB |
| ***lfa1785*** |  | protein of unknown function |
| ***lfa1786*** |  | Major facilitator superfamily MFS_1 |
| ***lfa1787*** |  | conserved exported protein of unknown function |
| ***lfa1788*** |  | exported protein of unknown function |
| ***lfa1789*** | ***def*** | Peptide deformylase 1 |
| ***lfa1790*** | ***gloA*** | Lactoylglutathione lyase |
| ***lfa1791*** |  | conserved exported protein of unknown function |
| ***lfa1792*** |  | protein of unknown function |
| ***lfa1793*** |  | conserved protein of unknown function |
| ***lfa1794*** |  | conserved protein of unknown function |
| ***lfa1795*** |  | protein of unknown function |
| ***lfa1796*** | ***acyP*** | Acylphosphatase |
| ***lfa1797*** |  | Muramoyltetrapeptide carboxypeptidase |
| ***lfa1798*** |  | conserved protein of unknown function |
| ***lfa1799*** |  | putative multidrug resistance protein NorM (Multidrug-efflux transporter) |
| ***lfa1801*** |  | exported protein of unknown function |
| ***lfa1802*** |  | Pentapeptide repeat protein |
| ***lfa1803*** |  | conserved exported protein of unknown function |
| ***lfa1804*** |  | conserved exported protein of unknown function |
| ***lfa1805*** |  | conserved membrane protein of unknown function |
| ***lfa1806*** |  | Radical SAM domain-containing protein |
| ***lfa1807*** |  | protein of unknown function |
| ***lfa1808*** |  | Putative sensory box/GGDEF domain protein (modular protein) |
| ***lfa1809*** |  | protein of unknown function |
| ***lfa1810*** |  | conserved protein of unknown function |
| ***lfa1811*** |  | putative O-antigen biosynthesis protein |
| ***lfa1812*** | ***epsL*** | Uncharacterized sugar transferase epsL |
| ***lfa1813*** |  | conserved protein of unknown function |
| ***lfa1814*** |  | protein of unknown function |
| ***lfa1815*** |  | putative O-antigen acetylase |
| ***lfa1816*** |  | putative ABC-2 |
| ***lfa1817*** | ***rfbE*** | O-antigen export system ATP-binding protein rfbE |
| ***lfa1818*** | ***yvfE*** | Pyridoxal phosphate-dependent enzyme |
| ***lfa1819*** |  | protein of unknown function |
| ***lfa1820*** |  | conserved exported protein of unknown function |
| ***lfa1821*** |  | membrane protein of unknown function |
| ***lfa1822*** |  | exported protein of unknown function |
| ***lfa1823*** | ***fabG*** | 3-oxoacyl-[acyl-carrier-protein] reductase 1 |
| ***lfa1824*** |  | conserved exported protein of unknown function |
| ***lfa1825*** |  | RNA-binding S1 domain-containing protein |
| ***lfa1827*** |  | protein of unknown function |
| ***lfa1828*** |  | DEAD/DEAH box helicase-like protein |
| ***lfa1829*** |  | putative outer membrane efflux protein |
| ***lfa1830*** |  | ABC-2 type transporter |
| ***lfa1831*** |  | putative ABC transporter, ATP-binding protein |
| ***lfa1832*** |  | putative lipoprotein |
| ***lfa1834*** | ***pncA*** | Pyrazinamidase/nicotinamidase |
| ***lfa1835*** | ***pncB*** | Nicotinate phosphoribosyltransferase |
| ***lfa1836*** |  | exported protein of unknown function |
| ***lfa1837*** | ***map*** | Major acid phosphatase |
| ***lfa1838*** |  | conserved protein of unknown function [coiled-coil domain] |
| ***lfa1839*** |  | protein of unknown function |
| ***lfa1840*** |  | similar to IcmL (DotI) protein [Macrophage killing IcmL/DotI domain] |
| ***lfa1841*** |  | conserved protein of unknown function |
| ***lfa1842*** |  | protein of unknown function |
| ***lfa1843*** |  | protein of unknown function [F-box domain containing protein] |
| ***lfa1844*** |  | Diguanylate cyclase (GGDEF) domain protein |
| ***lfa1845*** |  | conserved protein of unknown function [FIST domain, N-terminal] |
| ***lfa1846*** |  | putative Zeta toxin |
| ***lfa1847*** |  | Bacterial/Archaeal transporter family protein |
| ***lfa1848*** |  | membrane protein of unknown function |
| ***lfa1849*** | ***dctA*** | C4-dicarboxylate transport protein 2 |
| ***lfa1850*** |  | protein of unknown function |
| ***lfa1851*** | ***yjcE*** | Na+/H+ antiporter |
| ***lfa1852*** |  | membrane protein of unknown function |
| ***lfa1853*** |  | protein of unknown function [ankyrin domain] |
| ***lfa1854*** |  | protein of unknown function |
| ***lfa1855*** | ***ybaZ*** | putative methylated-DNA methyltransferase |
| ***lfa1856*** |  | conserved protein of unknown function |
| ***lfa1857*** |  | Bacterial periplasmic substrate-binding family protein (fragment) |
| ***lfa1858*** | ***argE*** | Acetylornithine deacetylase |
| ***lfa1859*** |  | protein of unknown function |
| ***lfa1860*** |  | protein of unknown function |
| ***lfa1861*** |  | Phage_integrase |
| ***lfa1862*** |  | Acetyltransferase, GNAT family |
| ***lfa1863*** | ***rimJ*** | Ribosomal-protein-alanine acetyltransferase |
| ***lfa1864*** |  | Alanyl tRNA synthetase |
| ***lfa1865*** |  | conserved protein of unknown function |
| ***lfa1866*** | ***ydcN*** | putative DNA-binding transcriptional regulator |
| ***lfa1867*** | ***usp*** | Universal stress protein (Usp) |
| ***lfa1868*** |  | substrate of the dot/icm secretion system |
| ***lfa1869*** |  | protein of unknown function |
| ***lfa1870*** |  | conserved protein of unknown function [ankyrin domain] |
| ***lfa1871*** | ***ftsH*** | ATP-dependent zinc metalloprotease |
| ***lfa1873*** |  | conserved protein of unknown function [coiled-coil domain] |
| ***lfa1874*** |  | Truncated hydrogenase expression/formation protein (fragment) |
| ***lfa1875*** |  | protein of unknown function |
| ***lfa1876*** |  | Nitroreductase |
| ***lfa1877*** |  | putative Phosphoribosyltransferase |
| ***lfa1878*** |  | conserved membrane protein of unknown function |
| ***lfa1879*** |  | protein of unknown function [ankyrin domain] |
| ***lfa1880*** |  | protein of unknown function |
| ***lfa1881*** |  | protein of unknown function [Leucine rich repeat containing protein] |
| ***lfa1882*** |  | protein of unknown function [Intron-encoded nuclease 2] |
| ***lfamisc_RNA_6*** | | Intron_gpII |
| ***lfa1883*** |  | conserved protein of unknown function [Host attachment protein] |
| ***lfa1884**** |  | **Erythromycin esterase** |
| ***lfa1885*** |  | protein of unknown function [Fic domain] |
| ***lfa1886*** |  | protein of unknown function |
| ***lfa1887*** |  | Aminoglycoside phosphotransferase |
| ***lfa1889*** | ***yacG*** | DNA gyrase inhibitor YacG |
| ***lfa1890*** |  | protein of unknown function |
| ***lfa1891*** |  | protein of unknown function |
| ***lfa1892*** |  | protein of unknown function |
| ***lfa1893*** |  | protein of unknown function |
| ***lfa1894*** |  | protein of unknown function [coiled-coil domain] |
| ***lfa1895*** |  | putative Pyridine nucleotide-disulphide oxidoreductase, NAD-binding region |
| ***lfa1897*** |  | conserved protein of unknown function [ras domain] |
| ***lfa1898*** |  | protein of unknown function |
| ***lfa1899*** |  | Regulator of chromosome condensation, rcc (substrate of the Dot/Icm secretion system) |
| ***lfa1900*** |  | conserved protein of unknown function [Fic domain] |
| ***lfa1901*** |  | protein of unknown function |
| ***lfa1903*** |  | putative Trypsin-like serine proteases |
| ***lfa1904*** |  | protein of unknown function |
| ***lfatRNA38*** | | Thr tRNA |
| ***lfa1905*** | ***rocC*** | Amino acid permease |
| ***lfa1906*** | ***csaA*** | Secretion chaperone CsaA |
| ***lfa1907*** |  | conserved protein of unknown function [ALPHA/BETA HYDROLASE RELATED] |
| ***lfa1908*** | ***capD*** | Capsular polysaccharide biosynthesis capD and UDP-glucose 4-epimerase |
| ***lfa1909*** |  | conserved membrane protein of unknown function |
| ***lfa1910*** |  | UbiA family prenyltransferase |
| ***lfa1911*** |  | substrate of the Dot/Icm secretion system |
| ***lfa1912*** | ***dnaE*** | DNA polymerase III subunit alpha |
| ***lfa1913*** |  | putative excinuclease ABC, C subunit, N-terminal |
| ***lfa1914*** |  | protein of unknown function |
| ***lfa1915*** |  | Peptide ABC transporter, permease protein |
| ***lfa1916*** |  | conserved membrane protein of unknown function |
| ***lfa1917*** |  | conserved protein of unknown function |
| ***lfa1918*** |  | RND efflux system, outer membrane lipoprotein, NodT family protein |
| ***lfa1919*** | ***emrA*** | Multidrug resistance protein A |
| ***lfa1920*** | ***emrB*** | Multidrug resistance protein B |
| ***lfa1921*** |  | protein of unknown function |
| ***lfa1922*** | ***ligA*** | DNA ligase |
| ***lfa1923*** |  | conserved exported protein of unknown function |
| ***lfa1924*** |  | conserved exported protein of unknown function |
| ***lfa1925*** | ***rmuC*** | RmuC family protein |
| ***lfa1926*** |  | Amino acid permease family protein |
| ***lfa1927*** |  | protein of unknown function |

*red predicted antibiotic resistance genes
