# Supplementary material for: Comparative analyses of Legionella species identifies genetic features of strains causing Legionnaires’ disease
Source: Genome Biol. 2014 Nov 3;15(11):505. doi: 10.1186/s13059-014-0505-0 (PMC4256840; doi:10.1186/s13059-014-0505-0)
Supplement: Additional file 6: Table S2. — Putative prophage region in the genome of L. micdadei strain ATCC33218. [file 13059_2014_505_MOESM6_ESM.docx]

**Table S2.** **Putative prophage region in the genome of *L. micdadei* strain ATCC33218.**

| ***Label*** | **Frame** | **Start** | **End** | **Size** | **Product** |
| --- | --- | --- | --- | --- | --- |
| ***lmi1636*** | -3 | 1733248 | 1734738 | 1491 | protein of unknown function |
| ***lmi1637*** | 1 | 1735420 | 1736580 | 1161 | protein of unknown function |
| ***lmi1638*** | -3 | 1736779 | 1738191 | 1413 | protein of unknown function |
| ***lmi1639**** | 2 | 1738655 | 1738918 | 264 | putative phage protein |
| ***lmi1640**** | 3 | 1738920 | 1739633 | 714 | phage replication P family protein |
| ***lmi1641*** | 2 | 1739621 | 1739827 | 207 | protein of unknown function |
| ***lmi1642*** | 1 | 1739788 | 1739949 | 162 | protein of unknown function |
| ***lmi1644*** | 3 | 1740123 | 1740371 | 249 | protein of unknown function |
| ***lmi1645*** | 3 | 1740387 | 1740857 | 471 | putative phage terminase small subunit |
| ***lmi1646*** | 3 | 1741335 | 1741718 | 384 | protein of unknown function |
| ***lmi1647*** | -3 | 1741846 | 1742187 | 342 | protein of unknown function |
| ***lmi1648*** | -2 | 1742273 | 1742650 | 378 | protein of unknown function |
| ***lmi1649*** | 2 | 1743005 | 1743214 | 210 | protein of unknown function |
| ***lmi1650**** | 2 | 1743227 | 1743784 | 558 | Transposon Tn21 resolvase |
| ***lmi1651**** | 2 | 1743827 | 1745365 | 1539 | Large subunit terminase TerL, phage protein [terminase_6 domain] |
| ***lmi1652**** | 1 | 1745530 | 1747623 | 2094 | putative phage portal protein [coiled-coil domain] |
| ***lmi1653*** | 2 | 1747613 | 1747771 | 159 | protein of unknown function |
| ***lmi1654*** | 3 | 1747740 | 1747910 | 171 | protein of unknown function |
| ***lmi1655*** | 2 | 1747907 | 1748113 | 207 | conserved protein of unknown function |
| ***lmi1656*** | 3 | 1748100 | 1748447 | 348 | conserved protein of unknown function |
| ***lmi1657*** | 2 | 1748444 | 1748650 | 207 | protein of unknown function |
| ***lmi1659*** | 1 | 1748755 | 1748907 | 153 | protein of unknown function |
| ***lmi1660**** | 2 | 1748909 | 1749094 | 186 | putative phage protein |
| ***lmi1661*** | 1 | 1749106 | 1749351 | 246 | protein of unknown function |
| ***lmi1662*** | 2 | 1749398 | 1750312 | 915 | protein of unknown function |
| ***lmi1663*** | -2 | 1750331 | 1750540 | 210 | protein of unknown function |
| ***lmi1664*** | 3 | 1750854 | 1751549 | 696 | protein of unknown function |
| ***lmi1665**** | 1 | 1751590 | 1752906 | 1317 | putative phage capside protein [P22_CoatProtein domain] |
| ***lmi1666**** | 2 | 1752962 | 1753828 | 867 | putative phage tail protein |
| ***lmi1667*** | 1 | 1753855 | 1754637 | 783 | protein of unknown function |
| ***lmi1668*** | 3 | 1754709 | 1754891 | 183 | protein of unknown function |
| ***lmi1669*** | 3 | 1754892 | 1756607 | 1716 | protein of unknown function |
| ***lmi1670*** | 2 | 1756607 | 1757110 | 504 | protein of unknown function |
| ***lmi1671**** | 2 | 1757123 | 1757740 | 618 | protein of unknown function [phage DNA injection protein motif] |
| ***lmi1672*** | 1 | 1757740 | 1758948 | 1209 | protein of unknown function |
| ***lmi1673*** | 2 | 1758941 | 1760128 | 1188 | protein of unknown function |
| ***lmi1674*** | 2 | 1760138 | 1762123 | 1986 | protein of unknown function |
| ***lmi1675*** | 3 | 1762161 | 1762523 | 363 | protein of unknown function |
| ***lmi1676*** | 3 | 1762560 | 1763801 | 1242 | protein of unknown function |
| ***lmi1677**** | 1 | 1763815 | 1764597 | 783 | putative phage tail fiber repeat protein |
| ***lmi1678*** | 1 | 1764607 | 1764855 | 249 | protein of unknown function |
| ***lmi1679*** | 3 | 1764852 | 1765382 | 531 | conserved protein of unknown function [Peptidoglycan binding domain] |
| ***lmi1680*** | 1 | 1765375 | 1765743 | 369 | protein of unknown function [VRR-NUC domain] |
| ***lmi1681*** | -3 | 1765798 | 1766127 | 330 | Keratin-associated protein 6-2 (fragment) |
| ***lmi1682**** | -1 | 1766124 | 1766426 | 303 | Putative phage associated protein |
| ***lmi1683*** | -3 | 1766461 | 1766889 | 429 | protein of unknown function |
| ***lmi1684**** | -1 | 1766886 | 1767737 | 852 | putative phage protein (fragment) |
| ***lmi1685*** | -1 | 1767903 | 1768379 | 477 | protein of unknown function |
| ***lmi1686*** | -3 | 1768405 | 1768599 | 195 | protein of unknown function |
| ***lmi1687**** | 3 | 1768746 | 1768946 | 201 | protein of unknown function [Lambda repressor-like, DNA-binding] |
| ***lmi1688*** | 1 | 1768987 | 1769313 | 327 | protein of unknown function |
| ***lmi1689*** | 2 | 1769366 | 1769485 | 120 | conserved protein of unknown function |
| ***lmi1690*** | 1 | 1769491 | 1769706 | 216 | conserved protein of unknown function [coiled-coil] |
| ***lmi1691*** | 2 | 1769795 | 1770016 | 222 | protein of unknown function |
| ***lmi1692*** | -3 | 1770025 | 1770180 | 156 | protein of unknown function |
| ***lmi1693*** | 1 | 1770106 | 1770450 | 345 | protein of unknown function [coiled-coil domain] |
| ***lmi1694*** | 2 | 1770512 | 1770742 | 231 | protein of unknown function [coiled-coil domain] |
| ***lmi1695*** | -1 | 1770744 | 1770911 | 168 | protein of unknown function |
| ***lmi1696*** | -2 | 1771277 | 1771399 | 123 | protein of unknown function |
| ***lmi1697**** | -1 | 1771410 | 1771601 | 192 | Phage transcriptional regulator, AlpA [Prophage CP4-57 regulatory domain] |
| ***lmi1698*** | 3 | 1771944 | 1772111 | 168 | protein of unknown function |
| ***lmi1699*** | -2 | 1772108 | 1772566 | 459 | protein of unknown function |
| ***lmi1700*** | 2 | 1772555 | 1772782 | 228 | protein of unknown function |
| ***lmi1701*** | 2 | 1772816 | 1773013 | 198 | protein of unknown function |
| ***lmi1702*** | 3 | 1773375 | 1773599 | 225 | protein of unknown function |
| ***lmi1703**** | 1 | 1773589 | 1774206 | 618 | putative phage replication protein |
| ***lmi1704*** | -1 | 1774722 | 1775585 | 864 | protein of unknown function |
| ***lmi1705*** | -2 | 1776005 | 1776172 | 168 | conserved protein of unknown function |
| ***lmi1706*** | 2 | 1776347 | 1777666 | 1320 | membrane protein of unknown function |
| ***lmi1707*** | 3 | 1777767 | 1778072 | 306 | conserved exported protein of unknown function |
| ***lmi1708*** | 2 | 1778171 | 1780105 | 1935 | conserved exported protein of unknown function |

*red predicted functions related to prophages
