# Supplementary material for: Comparative analyses of Legionella species identifies genetic features of strains causing Legionnaires’ disease
Source: Genome Biol. 2014 Nov 3;15(11):505. doi: 10.1186/s13059-014-0505-0 (PMC4256840; doi:10.1186/s13059-014-0505-0)
Supplement: Additional file 11: Table S3. — List of confirmed substrates of the Dot/Icm secretion system. [file 13059_2014_505_MOESM11_ESM.docx]

**Table S3**. List of confirmed substrates of the Dot/Icm secretion system

| **Gene name** | **Reference publications** | | |
| --- | --- | --- | --- |
| ***ravA*** | Lifshitz *et al.*2013 | Zhu *et al.* 2011 |  |
| ***cegC1*** | Lifshitz *et al.*2013 | Zhu *et al.* 2011 | Isberg *et al.* 2008 |
| ***-*** | Lifshitz *et al.*2013 | Zhu *et al.* 2011 |  |
| ***ravB*** | Lifshitz *et al.*2013 | Zhu *et al.* 2011 |  |
| ***legA10/ankQ*** | Lifshitz *et al.*2013 | Zhu *et al.* 2011 | Isberg *et al.* 2008 |
| ***-*** | Lifshitz *et al.*2013 | Zhu *et al.* 2011 | Isberg *et al.* 2008 |
| ***-*** | Lifshitz *et al.*2013 | Zhu *et al.* 2011 |  |
| ***ceg2*** | Lifshitz *et al.*2013 | Zhu *et al.* 2011 |  |
| ***ceg3*** | Lifshitz *et al.*2013 | Zhu *et al.* 2011 |  |
| ***-*** | Lifshitz *et al.*2013 | Zhu *et al.* 2011 | Isberg *et al.* 2008 |
| ***lem1*** | Lifshitz *et al.*2013 | Zhu *et al.* 2011 |  |
| ***ceg4*** | Lifshitz *et al.*2013 | Zhu *et al.* 2011 |  |
| ***vipF*** | Lifshitz *et al.*2013 | Zhu *et al.* 2011 | Isberg *et al.* 2008 |
| ***ravC*** | Lifshitz *et al.*2013 |  |  |
| ***cegC2*** | Lifshitz *et al.*2013 | Zhu *et al.* 2011 | Isberg *et al.* 2008 |
| ***-*** | Lifshitz *et al.*2013 | Zhu *et al.* 2011 |  |
| ***sdhB*** | Lifshitz *et al.*2013 |  |  |
| ***cetLP1*** | Lifshitz *et al.*2013 |  |  |
| ***ravD*** | Lifshitz *et al.*2013 | Zhu *et al.* 2011 |  |
| ***legU1*** | Lifshitz *et al.*2013 | Zhu *et al.* 2011 | Isberg *et al.* 2008 |
| ***-*** | Lifshitz *et al.*2013 | Zhu *et al.* 2011 |  |
| ***-*** | Lifshitz *et al.*2013 | Zhu *et al.* 2011 |  |
| ***ceg5*** | Lifshitz *et al.*2013 | Zhu *et al.* 2011 |  |
| ***ravE*** | Lifshitz *et al.*2013 | Zhu *et al.* 2011 |  |
| ***ravF*** | Lifshitz *et al.*2013 | Zhu *et al.* 2011 |  |
| ***ceg6/legK4*** | Lifshitz *et al.*2013 |  |  |
| ***mavR*** | Lifshitz *et al.*2013 |  |  |
| ***ravG*** | Lifshitz *et al.*2013 | Zhu *et al.* 2011 |  |
| ***ceg7*** | Lifshitz *et al.*2013 | Zhu *et al.* 2011 | Isberg *et al.* 2008 |
| ***sidE*** | Lifshitz *et al.*2013 | Zhu *et al.* 2011 | Isberg *et al.* 2008 |
| ***ceg8*** | Lifshitz *et al.*2013 | Zhu *et al.* 2011 |  |
| ***ceg9*** | Lifshitz *et al.*2013 | Zhu *et al.* 2011 |  |
| ***pelA*** | Lifshitz *et al.*2013 |  |  |
|  |  |  | Isberg *et al.* 2008 |
| ***-*** | Lifshitz *et al.*2013 | Zhu *et al.* 2011 |  |
| ***sdbA*** | Lifshitz *et al.*2013 |  |  |
| ***legG2*** | Lifshitz *et al.*2013 | Zhu *et al.* 2011 | Isberg *et al.* 2008 |
| ***ceg10*** | Lifshitz *et al.*2013 | Zhu *et al.* 2011 | Isberg *et al.* 2008 |
| ***lem2*** | Lifshitz *et al.*2013 | Zhu *et al.* 2011 |  |
| ***-*** | Lifshitz *et al.*2013 | Zhu *et al.* 2011 | Isberg *et al.* 2008 |
| ***-*** | Lifshitz *et al.*2013 | Zhu *et al.* 2011 |  |
| ***-*** | Lifshitz *et al.*2013 | Zhu *et al.* 2011 | Isberg *et al.* 2008 |
| ***-*** | Lifshitz *et al.*2013 | Zhu *et al.* 2011 |  |
| ***sdhA*** | Lifshitz *et al.*2013 | Zhu *et al.* 2011 | Isberg *et al.* 2008 |
| ***vipA*** | Lifshitz *et al.*2013 | Zhu *et al.* 2011 | Isberg *et al.* 2008 |
| ***cetLP2*** | Lifshitz *et al.*2013 |  |  |
| ***ceg11*** | Lifshitz *et al.*2013 | Zhu *et al.* 2011 | Isberg *et al.* 2008 |
| ***legA9/ceg12/ankY*** | Lifshitz *et al.*2013 | Zhu *et al.* 2011 | Isberg *et al.* 2008 |
| ***legA7/ankG/ankZ*** | Lifshitz *et al.*2013 | Zhu *et al.* 2011 |  |
| ***-*** | Lifshitz *et al.*2013 | Zhu *et al.* 2011 |  |
| ***legY*** |  | Zhu *et al.* 2011 |  |
| ***legA11/ankJ*** | Lifshitz *et al.*2013 | Zhu *et al.* 2011 | Isberg *et al.* 2008 |
| ***ceg14/sidL*** | Lifshitz *et al.*2013 | Zhu *et al.* 2011 |  |
| ***ceg15*** | Lifshitz *et al.*2013 | Zhu *et al.* 2011 |  |
| ***legA12/ankC*** | Lifshitz *et al.*2013 | Zhu *et al.* 2011 | Isberg *et al.* 2008 |
| ***legD2*** |  | Zhu *et al.* 2011 |  |
| ***-*** | Lifshitz *et al.*2013 | Zhu *et al.* 2011 | Isberg *et al.* 2008 |
| ***ceg17*** | Lifshitz *et al.*2013 | Zhu *et al.* 2011 |  |
| ***sidA*** | Lifshitz *et al.*2013 | Zhu *et al.* 2011 | Isberg *et al.* 2008 |
| ***-*** | Lifshitz *et al.*2013 | Zhu *et al.* 2011 | Isberg *et al.* 2008 |
| ***wipB*** | Lifshitz *et al.*2013 | Zhu *et al.* 2011 | Isberg *et al.* 2008 |
| ***mavS*** | Lifshitz *et al.*2013 |  |  |
| ***ligA*** |  |  |  |
| ***legA8/ankN/ankX*** | Lifshitz *et al.*2013 | Zhu *et al.* 2011 | Isberg *et al.* 2008 |
| ***lem3*** | Lifshitz *et al.*2013 | Zhu *et al.* 2011 |  |
|  |  | Zhu *et al.* 2011 |  |
| ***ravH*** | Lifshitz *et al.*2013 | Zhu *et al.* 2011 |  |
| ***-*** | Lifshitz *et al.*2013 | Zhu *et al.* 2011 |  |
| ***ceg18*** | Lifshitz *et al.*2013 | Zhu *et al.* 2011 | Isberg *et al.* 2008 |
| ***mavT*** | Lifshitz *et al.*2013 |  |  |
| ***ravI*** | Lifshitz *et al.*2013 | Zhu *et al.* 2011 |  |
| ***lidA*** | Lifshitz *et al.*2013 | Zhu *et al.* 2011 | Isberg *et al.* 2008 |
| ***ravJ*** | Lifshitz *et al.*2013 | Zhu *et al.* 2011 |  |
| ***legL1*** | Lifshitz *et al.*2013 | Zhu *et al.* 2011 | Isberg *et al.* 2008 |
| ***-*** | Lifshitz *et al.*2013 | Zhu *et al.* 2011 |  |
| ***sidK*** | Lifshitz *et al.*2013 | Zhu *et al.* 2011 |  |
| ***ravK*** | Lifshitz *et al.*2013 | Zhu *et al.* 2011 |  |
| ***-*** | Lifshitz *et al.*2013 | Zhu *et al.* 2011 |  |
| ***lem4/sdmA*** | Lifshitz *et al.*2013 | Zhu *et al.* 2011 |  |
| ***-*** | Lifshitz *et al.*2013 | Zhu *et al.* 2011 |  |
| ***ravL*** | Lifshitz *et al.*2013 | Zhu *et al.* 2011 |  |
| ***ravM*** | Lifshitz *et al.*2013 | Zhu *et al.* 2011 |  |
| ***lem5*** | Lifshitz *et al.*2013 | Zhu *et al.* 2011 |  |
| ***ravN*** | Lifshitz *et al.*2013 | Zhu *et al.* 2011 |  |
| ***lem6*** | Lifshitz *et al.*2013 | Zhu *et al.* 2011 |  |
| ***ceg19*** | Lifshitz *et al.*2013 | Zhu *et al.* 2011 |  |
| ***-*** | Lifshitz *et al.*2013 | Zhu *et al.* 2011 |  |
| ***ravO*** | Lifshitz *et al.*2013 | Zhu *et al.* 2011 |  |
| ***ceg20*** | Lifshitz *et al.*2013 | Zhu *et al.* 2011 |  |
| ***cegC3*** | Lifshitz *et al.*2013 | Zhu *et al.* 2011 | Isberg *et al.* 2008 |
| ***lem7*** | Lifshitz *et al.*2013 | Zhu *et al.* 2011 |  |
| ***-*** | Lifshitz *et al.*2013 | Zhu *et al.* 2011 |  |
| ***-*** | Lifshitz *et al.*2013 | Zhu *et al.* 2011 | Isberg *et al.* 2008 |
| ***ravP*** | Lifshitz *et al.*2013 | Zhu *et al.* 2011 |  |
| ***ravQ*** | Lifshitz *et al.*2013 | Zhu *et al.* 2011 |  |
| ***-*** | Lifshitz *et al.*2013 | Zhu *et al.* 2011 | Isberg *et al.* 2008 |
| ***ravR*** | Lifshitz *et al.*2013 | Zhu *et al.* 2011 |  |
| ***ceg21*** | Lifshitz *et al.*2013 | Zhu *et al.* 2011 |  |
| ***ravS*** | Lifshitz *et al.*2013 | Zhu *et al.* 2011 |  |
| ***vpdB*** | Lifshitz *et al.*2013 | Zhu *et al.* 2011 | Isberg *et al.* 2008 |
| ***-*** | Lifshitz *et al.*2013 | Zhu *et al.* 2011 | Isberg *et al.* 2008 |
| ***lem8*** | Lifshitz *et al.*2013 | Zhu *et al.* 2011 |  |
| ***legC1*** | Lifshitz *et al.*2013 | Zhu *et al.* 2011 |  |
| ***ravT*** | Lifshitz *et al.*2013 | Zhu *et al.* 2011 |  |
| ***ravW*** | Lifshitz *et al.*2013 | Zhu *et al.* 2011 |  |
|  |  | Zhu *et al.* 2011 |  |
| ***-*** | Lifshitz *et al.*2013 |  |  |
| ***sidG*** | Lifshitz *et al.*2013 | Zhu *et al.* 2011 | Isberg *et al.* 2008 |
| ***-*** | Lifshitz *et al.*2013 |  |  |
| ***lgt1*** | Lifshitz *et al.*2013 |  |  |
| ***LicA*** | Lifshitz *et al.*2013 |  |  |
| ***vpdC*** | Lifshitz *et al.*2013 | Zhu *et al.* 2011 |  |
| ***-*** | Lifshitz *et al.*2013 | Zhu *et al.* 2011 |  |
| ***-*** | Lifshitz *et al.*2013 | Zhu *et al.* 2011 |  |
| ***legK1*** | Lifshitz *et al.*2013 | Zhu *et al.* 2011 |  |
| ***ceg22*** | Lifshitz *et al.*2013 | Zhu *et al.* 2011 |  |
| ***legC5/lgt3*** | Lifshitz *et al.*2013 | Zhu *et al.* 2011 | Isberg *et al.* 2008 |
| ***ravX*** | Lifshitz *et al.*2013 | Zhu *et al.* 2011 |  |
| ***lem9*** | Lifshitz *et al.*2013 | Zhu *et al.* 2011 |  |
| ***lem10*** | Lifshitz *et al.*2013 | Zhu *et al.* 2011 |  |
| ***ravY*** | Lifshitz *et al.*2013 | Zhu *et al.* 2011 |  |
| ***-*** | Lifshitz *et al.*2013 | Zhu *et al.* 2011 |  |
| ***legC6*** | Lifshitz *et al.*2013 | Zhu *et al.* 2011 | Isberg *et al.* 2008 |
| ***lem11*** | Lifshitz *et al.*2013 | Zhu *et al.* 2011 |  |
| ***legL2*** | Lifshitz *et al.*2013 | Zhu *et al.* 2011 | Isberg *et al.* 2008 |
| ***ceg23*** | Lifshitz *et al.*2013 | Zhu *et al.* 2011 | Isberg *et al.* 2008 |
| ***lem12*** | Lifshitz *et al.*2013 | Zhu *et al.* 2011 |  |
| ***-*** | Lifshitz *et al.*2013 | Zhu *et al.* 2011 |  |
| ***sidB*** | Lifshitz *et al.*2013 | Zhu *et al.* 2011 | Isberg *et al.* 2008 |
| ***-*** | Lifshitz *et al.*2013 | Zhu *et al.* 2011 |  |
| ***legL3*** | Lifshitz *et al.*2013 | Zhu *et al.* 2011 | Isberg *et al.* 2008 |
| ***-*** | Lifshitz *et al.*2013 | Zhu *et al.* 2011 |  |
| ***cetLP3*** | Lifshitz *et al.*2013 |  |  |
| ***ceg24*** | Lifshitz *et al.*2013 | Zhu *et al.* 2011 |  |
| ***-*** | Lifshitz *et al.*2013 | Zhu *et al.* 2011 |  |
| ***-*** | Lifshitz *et al.*2013 | Zhu *et al.* 2011 |  |
| ***ravZ*** | Lifshitz *et al.*2013 | Zhu *et al.* 2011 |  |
| ***-*** | Lifshitz *et al.*2013 | Zhu *et al.* 2011 |  |
| ***-*** | Lifshitz *et al.*2013 | Zhu *et al.* 2011 |  |
| ***mavA*** | Lifshitz *et al.*2013 | Zhu *et al.* 2011 |  |
| ***-*** | Lifshitz *et al.*2013 | Zhu *et al.* 2011 | Isberg *et al.* 2008 |
| ***-*** | Lifshitz *et al.*2013 | Zhu *et al.* 2011 |  |
| ***legC3/ppeA*** | Lifshitz *et al.*2013 | Zhu *et al.* 2011 | Isberg *et al.* 2008 |
| ***lem13/ppeB*** | Lifshitz *et al.*2013 | Zhu *et al.* 2011 |  |
| ***-*** | Lifshitz *et al.*2013 | Zhu *et al.* 2011 |  |
| ***-*** | Lifshitz *et al.*2013 | Zhu *et al.* 2011 | Isberg *et al.* 2008 |
| ***legAS4/ankI*** | Lifshitz *et al.*2013 | Zhu *et al.* 2011 | Isberg *et al.* 2008 |
| ***-*** | Lifshitz *et al.*2013 | Zhu *et al.* 2011 | Isberg *et al.* 2008 |
| ***mavB*** | Lifshitz *et al.*2013 | Zhu *et al.* 2011 |  |
| ***-*** | Lifshitz *et al.*2013 | Zhu *et al.* 2011 |  |
| ***rvfA*** | Lifshitz *et al.*2013 | Zhu *et al.* 2011 |  |
| ***mavU*** | Lifshitz *et al.*2013 | Zhu *et al.* 2011 |  |
| ***-*** | Lifshitz *et al.*2013 | Zhu *et al.* 2011 |  |
| ***cetLP4*** | Lifshitz *et al.*2013 |  |  |
| ***ceg25*** | Lifshitz *et al.*2013 |  |  |
| ***lem14*** | Lifshitz *et al.*2013 | Zhu *et al.* 2011 |  |
| ***ylfB/legC2*** | Lifshitz *et al.*2013 | Zhu *et al.* 2011 | Isberg *et al.* 2008 |
| ***-*** | Lifshitz *et al.*2013 | Zhu *et al.* 2011 |  |
| ***legLC8*** | Lifshitz *et al.*2013 | Zhu *et al.* 2011 | Isberg *et al.* 2008 |
| ***-*** | Lifshitz *et al.*2013 | Zhu *et al.* 2011 |  |
| ***-*** | Lifshitz *et al.*2013 | Zhu *et al.* 2011 |  |
| ***lem15*** | Lifshitz *et al.*2013 | Zhu *et al.* 2011 |  |
| ***lem16*** | Lifshitz *et al.*2013 | Zhu *et al.* 2011 |  |
| ***legLC4*** | Lifshitz *et al.*2013 | Zhu *et al.* 2011 | Isberg *et al.* 2008 |
| ***lem17*** | Lifshitz *et al.*2013 | Zhu *et al.* 2011 |  |
| ***ralF*** | Lifshitz *et al.*2013 | Zhu *et al.* 2011 | Isberg *et al.* 2008 |
| ***legC4*** | Lifshitz *et al.*2013 | Zhu *et al.* 2011 | Isberg *et al.* 2008 |
| ***legL5*** | Lifshitz *et al.*2013 | Zhu *et al.* 2011 | Isberg *et al.* 2008 |
| ***-*** | Lifshitz *et al.*2013 | Zhu *et al.* 2011 |  |
| ***lirA*** | Lifshitz *et al.*2013 | Zhu *et al.* 2011 |  |
| ***lirB*** | Lifshitz *et al.*2013 | Zhu *et al.* 2011 |  |
| ***lirC/pieA*** | Lifshitz *et al.*2013 | Zhu *et al.* 2011 |  |
| ***lirD/pieB*** | Lifshitz *et al.*2013 | Zhu *et al.* 2011 |  |
| ***lirE/pieC*** | Lifshitz *et al.*2013 | Zhu *et al.* 2011 |  |
| ***lirF/pieD*** | Lifshitz *et al.*2013 | Zhu *et al.* 2011 |  |
| ***lem18/pieE*** | Lifshitz *et al.*2013 | Zhu *et al.* 2011 |  |
| ***pieF*** | Lifshitz *et al.*2013 | Zhu *et al.* 2011 |  |
| ***legG1/pieG/pele*** | Lifshitz *et al.*2013 | Zhu *et al.* 2011 | Isberg *et al.* 2008 |
| ***setA*** | Lifshitz *et al.*2013 | Zhu *et al.* 2011 |  |
| ***-*** | Lifshitz *et al.*2013 | Zhu *et al.* 2011 |  |
| ***-*** | Lifshitz *et al.*2013 | Zhu *et al.* 2011 |  |
|  |  | Zhu *et al.* 2011 |  |
| ***legK2*** | Lifshitz *et al.*2013 | Zhu *et al.* 2011 | Isberg *et al.* 2008 |
| ***legAU13/ceg27/ankB*** | Lifshitz *et al.*2013 | Zhu *et al.* 2011 | Isberg *et al.* 2008 |
| ***mavC*** | Lifshitz *et al.*2013 | Zhu *et al.* 2011 |  |
| ***-*** | Lifshitz *et al.*2013 | Zhu *et al.* 2011 |  |
| ***-*** | Lifshitz *et al.*2013 | Zhu *et al.* 2011 |  |
| ***sdeC*** | Lifshitz *et al.*2013 | Zhu *et al.* 2011 |  |
| ***sidJ*** | Lifshitz *et al.*2013 | Zhu *et al.* 2011 | Isberg *et al.* 2008 |
| ***sdeB*** | Lifshitz *et al.*2013 | Zhu *et al.* 2011 |  |
| ***sdeA*** | Lifshitz *et al.*2013 | Zhu *et al.* 2011 | Isberg *et al.* 2008 |
| ***-*** | Lifshitz *et al.*2013 |  |  |
| ***lem19*** | Lifshitz *et al.*2013 | Zhu *et al.* 2011 |  |
| ***-*** | Lifshitz *et al.*2013 |  |  |
| ***legS2*** | Lifshitz *et al.*2013 | Zhu *et al.* 2011 | Isberg *et al.* 2008 |
| ***mavD*** | Lifshitz *et al.*2013 | Zhu *et al.* 2011 |  |
| ***cegC4*** | Lifshitz *et al.*2013 | Zhu *et al.* 2011 | Isberg *et al.* 2008 |
| ***legA2*** | Lifshitz *et al.*2013 | Zhu *et al.* 2011 | Isberg *et al.* 2008 |
| ***lem20*** | Lifshitz *et al.*2013 | Zhu *et al.* 2011 |  |
| ***lpnE*** | Lifshitz *et al.*2013 |  |  |
| ***-*** | Lifshitz *et al.*2013 | Zhu *et al.* 2011 |  |
| ***ppgA*** | Lifshitz *et al.*2013 | Zhu *et al.* 2011 |  |
| ***-*** | Lifshitz *et al.*2013 | Zhu *et al.* 2011 |  |
| ***cetLP5*** | Lifshitz *et al.*2013 |  |  |
| ***lem21*** | Lifshitz *et al.*2013 | Zhu *et al.* 2011 |  |
| ***-*** | Lifshitz *et al.*2013 | Zhu *et al.* 2011 |  |
| ***cetLP6*** | Lifshitz *et al.*2013 |  |  |
| ***ylfA/legC7*** | Lifshitz *et al.*2013 | Zhu *et al.* 2011 | Isberg *et al.* 2008 |
| ***legA3/ankH/ankW*** | Lifshitz *et al.*2013 | Zhu *et al.* 2011 | Isberg *et al.* 2008 |
| ***ceg28*** | Lifshitz *et al.*2013 | Zhu *et al.* 2011 |  |
| ***legA5/ankK*** | Lifshitz *et al.*2013 | Zhu *et al.* 2011 | Isberg *et al.* 2008 |
| ***-*** | Lifshitz *et al.*2013 | Zhu *et al.* 2011 | Isberg *et al.* 2008 |
| ***lem22*** | Lifshitz *et al.*2013 | Zhu *et al.* 2011 |  |
| ***mavE*** | Lifshitz *et al.*2013 | Zhu *et al.* 2011 |  |
| ***mavF*** | Lifshitz *et al.*2013 | Zhu *et al.* 2011 |  |
| ***-*** | Lifshitz *et al.*2013 | Zhu *et al.* 2011 |  |
| ***-*** | Lifshitz *et al.*2013 | Zhu *et al.* 2011 |  |
| ***-*** | Lifshitz *et al.*2013 | Zhu *et al.* 2011 |  |
| ***pelF*** | Lifshitz *et al.*2013 |  |  |
| ***-*** | Lifshitz *et al.*2013 | Zhu *et al.* 2011 |  |
| ***sdbC*** | Lifshitz *et al.*2013 | Zhu *et al.* 2011 |  |
| ***legL6*** | Lifshitz *et al.*2013 | Zhu *et al.* 2011 |  |
| ***legL7*** | Lifshitz *et al.*2013 | Zhu *et al.* 2011 | Isberg *et al.* 2008 |
| ***lem23*** | Lifshitz *et al.*2013 | Zhu *et al.* 2011 |  |
| ***-*** | Lifshitz *et al.*2013 | Zhu *et al.* 2011 | Isberg *et al.* 2008 |
| ***ceg29*** | Lifshitz *et al.*2013 | Zhu *et al.* 2011 | Isberg *et al.* 2008 |
| ***vpdA*** | Lifshitz *et al.*2013 | Zhu *et al.* 2011 | Isberg *et al.* 2008 |
| ***lem24*** | Lifshitz *et al.*2013 | Zhu *et al.* 2011 |  |
| ***sdbB*** | Lifshitz *et al.*2013 |  |  |
| ***LegA1*** |  | Zhu *et al.* 2011 |  |
| ***-*** | Lifshitz *et al.*2013 | Zhu *et al.* 2011 |  |
| ***lem25*** | Lifshitz *et al.*2013 | Zhu *et al.* 2011 |  |
| ***mavG*** | Lifshitz *et al.*2013 | Zhu *et al.* 2011 |  |
| ***mavH*** | Lifshitz *et al.*2013 | Zhu *et al.* 2011 |  |
| ***ceg30*** | Lifshitz *et al.*2013 | Zhu *et al.* 2011 |  |
| ***-*** | Lifshitz *et al.*2013 | Zhu *et al.* 2011 |  |
| ***-*** | Lifshitz *et al.*2013 | Zhu *et al.* 2011 |  |
| ***mavI*** | Lifshitz *et al.*2013 | Zhu *et al.* 2011 |  |
| ***legA14/ceg31/ankF*** | Lifshitz *et al.*2013 | Zhu *et al.* 2011 | Isberg *et al.* 2008 |
| ***legA15/ankD*** | Lifshitz *et al.*2013 | Zhu *et al.* 2011 | Isberg *et al.* 2008 |
| ***-*** | Lifshitz *et al.*2013 | Zhu *et al.* 2011 |  |
| ***sidM/drrA*** | Lifshitz *et al.*2013 | Zhu *et al.* 2011 | Isberg *et al.* 2008 |
| ***sidD*** | Lifshitz *et al.*2013 | Zhu *et al.* 2011 | Isberg *et al.* 2008 |
| ***lepB*** | Lifshitz *et al.*2013 | Zhu *et al.* 2011 | Isberg *et al.* 2008 |
| ***sdbB*** | Lifshitz *et al.*2013 |  |  |
| ***mavJ*** | Lifshitz *et al.*2013 | Zhu *et al.* 2011 |  |
| ***ceg32/sidI*** | Lifshitz *et al.*2013 | Zhu *et al.* 2011 |  |
| ***-*** | Lifshitz *et al.*2013 | Zhu *et al.* 2011 |  |
| ***sdjA*** | Lifshitz *et al.*2013 | Zhu *et al.* 2011 | Isberg *et al.* 2008 |
| ***sdeD*** | Lifshitz *et al.*2013 | Zhu *et al.* 2011 |  |
| ***sdcA*** | Lifshitz *et al.*2013 | Zhu *et al.* 2011 |  |
| ***sidC*** | Lifshitz *et al.*2013 | Zhu *et al.* 2011 | Isberg *et al.* 2008 |
| ***lem26*** | Lifshitz *et al.*2013 | Zhu *et al.* 2011 |  |
| ***mavK*** | Lifshitz *et al.*2013 | Zhu *et al.* 2011 |  |
| ***mavL*** | Lifshitz *et al.*2013 | Zhu *et al.* 2011 |  |
| ***lnaB*** | Lifshitz *et al.*2013 | Zhu *et al.* 2011 | Isberg *et al.* 2008 |
| ***lem27*** | Lifshitz *et al.*2013 | Zhu *et al.* 2011 |  |
| ***-*** | Lifshitz *et al.*2013 | Zhu *et al.* 2011 |  |
| ***-*** | Lifshitz *et al.*2013 | Zhu *et al.* 2011 |  |
| ***pelH*** | Lifshitz *et al.*2013 | Zhu *et al.* 2011 |  |
| ***-*** | Lifshitz *et al.*2013 | Zhu *et al.* 2011 |  |
| ***-*** | Lifshitz *et al.*2013 | Zhu *et al.* 2011 |  |
| ***-*** | Lifshitz *et al.*2013 | Zhu *et al.* 2011 |  |
| ***legK3*** | Lifshitz *et al.*2013 | Zhu *et al.* 2011 | Isberg *et al.* 2008 |
| ***mavM*** | Lifshitz *et al.*2013 | Zhu *et al.* 2011 |  |
| ***sidF*** | Lifshitz *et al.*2013 | Zhu *et al.* 2011 | Isberg *et al.* 2008 |
| ***LegS1*** |  | Zhu *et al.* 2011 |  |
| ***ceg33*** | Lifshitz *et al.*2013 | Zhu *et al.* 2011 | Isberg *et al.* 2008 |
| ***lem28/sdmB*** | Lifshitz *et al.*2013 | Zhu *et al.* 2011 |  |
| ***-*** | Lifshitz *et al.*2013 | Zhu *et al.* 2011 |  |
| ***-*** | Lifshitz *et al.*2013 | Zhu *et al.* 2011 |  |
| ***mavV*** | Lifshitz *et al.*2013 | Zhu *et al.* 2011 |  |
| ***-*** | Lifshitz *et al.*2013 | Zhu *et al.* 2011 |  |
| ***legD1*** |  | Zhu *et al.* 2011 |  |
| ***wipA*** | Lifshitz *et al.*2013 | Zhu *et al.* 2011 | Isberg *et al.* 2008 |
|  |  | Zhu *et al.* 2011 |  |
| ***-*** | Lifshitz *et al.*2013 | Zhu *et al.* 2011 | Isberg *et al.* 2008 |
| ***-*** | Lifshitz *et al.*2013 | Zhu *et al.* 2011 |  |
| ***lepA*** | Lifshitz *et al.*2013 | Zhu *et al.* 2011 | Isberg *et al.* 2008 |
| ***lem29*** | Lifshitz *et al.*2013 | Zhu *et al.* 2011 |  |
| ***cetLP7*** | Lifshitz *et al.*2013 |  |  |
| ***mavN*** | Lifshitz *et al.*2013 | Zhu *et al.* 2011 |  |
| ***ceg34*** | Lifshitz *et al.*2013 | Zhu *et al.* 2011 |  |
| ***-*** | Lifshitz *et al.*2013 | Zhu *et al.* 2011 |  |
| ***sidH*** | Lifshitz *et al.*2013 | Zhu *et al.* 2011 | Isberg *et al.* 2008 |
| ***legU2/lubX*** | Lifshitz *et al.*2013 | Zhu *et al.* 2011 | Isberg *et al.* 2008 |
| ***vipD*** | Lifshitz *et al.*2013 | Zhu *et al.* 2011 | Isberg *et al.* 2008 |
| ***-*** | Lifshitz *et al.*2013 | Zhu *et al.* 2011 |  |
| ***-*** | Lifshitz *et al.*2013 | Zhu *et al.* 2011 |  |
| ***legC8/lgt2*** | Lifshitz *et al.*2013 | Zhu *et al.* 2011 | Isberg *et al.* 2008 |
| ***-*** | Lifshitz *et al.*2013 | Zhu *et al.* 2011 |  |
| ***mavO*** | Lifshitz *et al.*2013 | Zhu *et al.* 2011 |  |
| ***mavP*** | Lifshitz *et al.*2013 | Zhu *et al.* 2011 |  |
| ***-*** | Lifshitz *et al.*2013 | Zhu *et al.* 2011 |  |
| ***-*** | Lifshitz *et al.*2013 | Zhu *et al.* 2011 |  |
| ***mavW*** | Lifshitz *et al.*2013 |  |  |
| ***-*** | Lifshitz *et al.*2013 | Zhu *et al.* 2011 |  |
| ***-*** | Lifshitz *et al.*2013 | Zhu *et al.* 2011 |  |
| ***mavQ*** | Lifshitz *et al.*2013 | Zhu *et al.* 2011 |  |
| ***legP*** | Lifshitz *et al.*2013 | Zhu *et al.* 2011 | Isberg *et al.* 2008 |
| ***-*** | Lifshitz *et al.*2013 | Zhu *et al.* 2011 |  |

**NOTE :** Mistakes that were present in the referenced lists have been removed from this updated list: *lpg0041, lpg0170, lpg0963* (erroneously confounded with the effector lpg0693)
