# Supplementary material for: Comparative analyses of Legionella species identifies genetic features of strains causing Legionnaires’ disease
Source: Genome Biol. 2014 Nov 3;15(11):505. doi: 10.1186/s13059-014-0505-0 (PMC4256840; doi:10.1186/s13059-014-0505-0)
Supplement: Additional file 12: Table S4. — List of eukaryotic organisms and viruses whose genomes have been selected for the construction of the eukaryotic genome database for Blast searches. [file 13059_2014_505_MOESM12_ESM.docx]

**Table S4***.* List of eukaryotic organisms and viruses whose genomes have been selected for the construction of the eukaryotic genome database for blast searches.

| ***SuperPhylum*** | ***Taxon*** |
| --- | --- |
| *Alveolata* | *Cryptosporidium parvum Iowa II* |
| *Alveolata* | *Plasmodium falciparum* |
| *Alveolata* | *Theileria annulata* |
| *Alveolata* | *Theileria parva* |
| *Alveolata* | *Toxoplasma Gondii Apicoplast* |
| *Alveolata* | *Paramecium tetraurelia* |
| *Alveolata* | *Tetrahymena thermophila* |
| *Alveolata* | *Bavesia bovis* |
| *Amoebozoa* | *Entamoeba dispar* |
| *Amoebozoa* | *Entamoeba histolytica* |
| *Amoebozoa* | *Entamoeba invadens* |
| *Amoebozoa* | *Entamoeba moshkovskii* |
| *Amoebozoa* | *Acanthamoeba castellanii* |
| *Amoebozoa* | *Dictyostelium discoideum* |
| *Amoebozoa* | *Dictyostelium purpureum* |
| *Amoebozoa* | *Dictyostelium fasciculatum* |
| *Amoebozoa* | *Polysphondylium pallidum* |
| *Excavata* | *Leishmania infantum* |
| *Excavata* | *Leishmania major* |
| *Excavata* | *Leishmania braziliensis* |
| *Excavata* | *Naegleria gruberi* |
| *Excavata* | *Trypanosoma brucei* |
| *Excavata* | *Trypanosoma cruzi* |
| *Excavata* | *Giardia intestinalis* |
| *Excavata* | *Giardia lamblia ATCC* |
| *Excavata* | *Trichomonas vaginalis* |
| *Haptophyta* | *Emiliania huxleyi* |
| *Heterokonta* | *Phaeodactylum tricornutum* |
| *Heterokonta* | *Thalassiosira pseudonana* |
| *Heterokonta* | *Phytophthora capsici* |
| *Heterokonta* | *Phytophthora infestans* |
| *Heterokonta* | *Phytophthora ramorum* |
| *Heterokonta* | *Phytophthora sojae* |
| *Heterokonta* | *Pythium ultimum* |
| *Heterokonta* | *Aureococcus anophagefferens* |
| *Heterokonta* | *Blastocystis hominis* |
| *Opisthokonta* | *Capsaspora owczarzaki* |
| *Opisthokonta* | *Salpingoeca rosetta* |
| *Opisthokonta* | *Ashbya "Eremothecium" gossypii* |
| *Opisthokonta* | *Aspergillus fumigatus Af293* |
| *Opisthokonta* | *Batrachochytrium dendrobatidis* |
| *Opisthokonta* | *Candida glabrata* |
| *Opisthokonta* | *Cryptococcus neoformans var. neoformans JEC21* |
| *Opisthokonta* | *Debaryomyces hansenii CBS767* |
| *Opisthokonta* | *Gibberella zeae* |
| *Opisthokonta* | *Kluyveromyces lactis* |
| *Opisthokonta* | *Laccaria bicolor* |
| *Opisthokonta* | *Phanerochaete chrysosporium* |
| *Opisthokonta* | *Phycomyces blakesleeanus* |
| *Opisthokonta* | *Postia placenta* |
| *Opisthokonta* | *Puccinia graminis* |
| *Opisthokonta* | *Saccharomyces cerevisiae* |
| *Opisthokonta* | *Schizosaccharomyces pombe* |
| *Opisthokonta* | *Sporobolomyces roseus* |
| *Opisthokonta* | *Caenorhabditis elegans* |
| *Opisthokonta* | *Ciona intestinalis* |
| *Opisthokonta* | *Danio rerio* |
| *Opisthokonta* | *Helobdella robusta* |
| *Opisthokonta* | *Homo sapiens* |
| *Opisthokonta* | *Mus Musculus* |
| *Opisthokonta* | *Tribolium castaneum* |
| *Opisthokonta* | *Yarrowia lipolytica* |
| *Opisthokonta* | *Ustilago maydis* |
| *Opisthokonta* | *Coprinopsis cinerea* |
| *Opisthokonta* | *Strongylocentrus purpuratus* |
| *Plantae* | *Chlorella variabilis* |
| *Plantae* | *Ostreococcus lucimarinus* |
| *Plantae* | *Volvox carteri* |
| *Plantae* | *Cyanidioschyzon merolae* |
| *Plantae* | *Arabidopsis thaliana* |
| *Plantae* | *Chlamydomonas reinhardtii* |
| *Plantae* | *Glycine max* |
| *Plantae* | *Oryza sativa* |
| *Plantae* | *Physcomitrella patens* |
| *Plantae* | *Selaginella moellendorffii* |
| *Virus* | *Acanthamoeba polyphaga mimivirus* |
| *Virus* | *Cafeteria roenbergensis virus* |
| *Virus* | *Emiliania huxleyi virus 202* |
| *Virus* | *Emiliania huxleyi virus 203* |
| *Virus* | *Emiliania huxleyi virus 84* |
| *Virus* | *Lausannevirus* |
| *Virus* | *Marseillevirus* |
| *Virus* | *Megavirus chiliensis* |
