# Supplementary material for: Comparative analyses of Legionella species identifies genetic features of strains causing Legionnaires’ disease
Source: Genome Biol. 2014 Nov 3;15(11):505. doi: 10.1186/s13059-014-0505-0 (PMC4256840; doi:10.1186/s13059-014-0505-0)
Supplement: Additional file 13: Table S5 — Orthologous genes encoding eukaryotic motifs in five Legionella species. [file 13059_2014_505_MOESM13_ESM.docx]

**Table S5. Orthologous genes encoding eukaryotic motifs in 5 *Legionella* species**

| ***L. pneumophila* (Philadelpia)**  **(Philadelphia)** | ***L. longbeachae*** | ***L. micdadei*** | ***L. hackeliae*** | ***L. fallonii* (LLAP10)** | **Name** | **Synonym** | **Eukaryotic motif** |
| --- | --- | --- | --- | --- | --- | --- | --- |
|  |  |  |  |  |  |  |  |
| *lpg0038** |  |  |  |  | *ankQ* | *legA10* | ANK |
| *lpg0112* | *llo1394* | *lmi0478* |  |  |  |  | ANK |
| *lpg0402** |  |  | *lha0494* | *lfa0056* | *ankY* | *legA9* | ANK |
| *lpg0403** |  |  | *lha0385* | *lfa2715* | *ankG* | *ankZ, legA7* | ANK |
| *lpg0436** |  |  |  |  | *ankJ* | *legA11* | ANK |
| *lpg0483** | *llo2705* | *lmi0828* | *lha0593* | *lfa2641* | *ankC* | *legA12* | ANK |
| *lpg0695** |  |  |  |  | *ankN* | *ankX legA8* | ANK |
| *lpg1718** |  | *lmi2895* |  | *lfa1741* | *ankI* | *legAS4* | ANK +SET |
| *lpg2131** |  |  |  |  |  |  | ANK |
| *lpg2144** |  |  |  |  | *ankB* | *legAU13, ceg27* | ANK + Fbox |
| *lpg2215** |  |  |  | *lfa1868#* |  |  | ANK |
| *lpg2300** | *llo0584* | *lmi2612* | *lha0726* | *lfa2306* | *ankH* | *legA3, ankW* | ANK |
| *lpg2322** | *llo0570* | *lmi1004#* | *lha0901#* | *lfa2332* | *ankK* | *legA5* | ANK |
| *lpg2416** |  |  |  |  |  |  | ANK |
| *lpg2452** |  |  |  |  | *ankF* | *legA14. ceg31* | ANK |
| *lpg2456** | *llo0365#* |  |  | *lfa1112 #* | *ankD* |  | ANK |
|  | *llo0037* |  |  |  |  |  | ANK |
|  | *llo0087* |  |  | *lfa1986#* |  |  | ANK |
|  | *llo0115* |  |  |  |  |  | ANK |
|  | *llo0246* |  |  |  |  |  | ANK |
|  | *llo0990* |  |  |  |  |  | ANK |
|  | *llo1043* |  |  |  |  |  | ANK |
|  | *llo1142* |  |  |  |  |  | ANK |
|  | *llo1168* |  |  |  |  |  | ANK |
|  | *llo1371* |  |  |  |  |  | ANK |
|  | *llo1395* |  |  |  |  |  | ANK |
|  | *llo1618* |  |  |  |  |  | ANK |
|  | *llo1646* |  |  |  |  |  | ANK |
|  | *llo1651* |  |  |  |  |  | ANK |
|  | *llo1715* |  |  |  |  |  | ANK |
|  | *llo1742* | *lmi2399* |  |  |  |  | ANK |
|  | *llo1894* |  |  |  |  |  | ANK |
|  | *llo2133a* |  |  |  |  |  | ANK |
|  | *llo2133b* |  |  |  |  |  | ANK |
|  | *llo2476* |  |  |  |  |  | ANK |
|  | *llo2668* |  |  |  |  |  | ANK |
|  | *llo3081* |  |  |  |  |  | ANK |
|  | *llo3093* |  |  | *lfa1853* |  |  | ANK |
|  | *llo3343* |  |  |  |  |  | ANK |
|  | *llo3353* |  |  |  |  |  | ANK |
|  |  | *lmi0705* |  |  |  |  | ANK |
|  |  | *lmi0728* |  |  |  |  | ANK |
|  |  | *lmi0891* |  | *lfa3318* |  |  | ANK + STPK + Coiled coil |
|  |  | *lmi1270* |  |  |  |  | ANK |
|  |  | *lmi1792* | *lha1864* |  |  |  | ANK |
|  |  | *lmi1922* |  |  |  |  | ANK |
|  |  | *lmi2155* |  |  |  |  | ANK |
|  |  | *lmi2156* |  |  |  |  | ANK |
|  |  | *lmi2208* |  |  |  |  | ANK |
|  |  | *lmi2222* |  |  |  |  | ANK |
|  |  | *lmi2464* |  |  |  |  | ANK |
|  |  | *lmi2545* |  | *lfa1498* |  |  | ANK + RasGEF |
|  |  | *lmi2663* |  |  |  |  | ANK + Coiled coil |
|  |  | *lmi2776* |  |  |  |  | ANK |
|  |  | *lmi2988* |  |  |  |  | ANK |
|  |  |  |  | *lfa0474* |  |  | ANK |
|  |  |  |  | *lfa0492* |  |  | ANK |
|  |  |  |  | *lfa0515* |  |  | ANK |
|  |  |  |  | *lfa0620* |  |  | ANK |
|  |  |  |  | *lfa0665* |  |  | ANK |
|  |  |  |  | *lfa0668* |  |  | ANK |
|  |  |  |  | *lfa0737* |  |  | ANK |
|  |  |  |  | *lfa1116* |  |  | ANK |
|  |  |  |  | *lfa1470* |  |  | ANK |
|  |  |  |  | *lfa1472* |  |  | ANK |
|  |  |  |  | *lfa1781* |  |  | ANK |
|  |  |  |  | *lfa1870* |  |  | ANK |
|  |  |  |  | *lfa1879* |  |  | ANK |
|  |  |  |  | *lfa2196* |  |  | ANK |
|  |  |  |  | *lfa2471* |  |  | ANK |
|  |  |  |  | *lfa2756* |  |  | ANK |
|  |  |  |  | *lfa3127* |  |  | ANK |
|  |  |  |  | *lfa3228* |  |  | ANK |
|  |  |  |  | *lfa3786* |  |  | ANK |
|  |  |  | *lha0686* |  |  |  | ANK |
|  |  |  | *lha0848* |  |  |  | ANK |
|  |  |  | *lha1268* |  |  |  | ANK |
|  |  |  | *lha1535* |  |  |  | ANK |
|  |  |  | *lha1536* |  |  |  | ANK |
|  |  |  | *lha1626* |  |  |  | ANK |
|  |  |  | *lha1695* |  |  |  | ANK |
|  |  |  | *lha1733* |  |  |  | ANK |
|  |  |  | *lha2367* |  |  |  | ANK |
|  |  |  | *lha3012* |  |  |  | ANK |
|  |  |  | *lha3020* |  |  |  | ANK |
|  |  |  | *lha0100* |  |  |  | ANK + LRR |
|  |  |  |  | *lfa1445* |  |  | ANK + SET |
|  |  |  |  | *lfa2096* |  |  | ANK + STPK |
|  |  |  | *lha0160* |  |  |  | ANK +F box |
|  |  |  |  | *lfa0693* |  |  | ANK +Fbox |
|  |  |  | *lha2960* |  |  |  | ANK +STPK +Coiled coil |
| *lpg0135*#* | *llo2439* |  |  | *lfa1659#* |  |  | Coiled coil |
| *lpg0744#* |  | *lmi0648* | *lha1156#* | *lfa2087 #* |  |  | Coiled coil |
| *lpg1355** |  |  |  |  | *sidG* |  | Coiled coil + spectrin |
| *lpg1588** |  |  |  |  |  |  | Coiled coil |
| *lpg1701** |  |  |  | *lfa1717* | *ppeA* |  | Coiled coil |
| *lpg1702** |  |  |  |  | *ppeB* |  | Coiled coil |
| *lpg1969*#* | *llo3131* |  |  | *lfa2198* | *pieE* |  | Coiled coil |
| *lpg2156** |  |  |  |  |  |  | Coiled coil |
| *lpg2490** |  |  |  |  | *lepB* |  | Coiled coil |
| *lpg2620#* | *llo2762* | *lmi0314* | *lha2911* | *lfa2554* |  |  | Coiled coil |
| *lpg2793** |  |  |  |  | *lepA* |  | Coiled coil |
| *lpg2829** |  |  |  |  | *sidH* |  | Coiled coil |
|  | *llo1458* |  |  |  |  |  | Coiled coil + spectrin |
|  | *llo1954* |  |  |  |  |  | Coiled coil |
|  | *llo2313* |  |  |  |  |  | Coiled coil |
|  | *llo2809* |  |  |  |  |  | Coiled coil + spectrine |
|  | *llo2868* |  |  |  |  |  | Coiled coil |
|  | *llo2870* |  |  |  |  |  | Coiled coil |
|  | *llo3158* |  |  |  |  |  | Coiled coil |
|  |  | *lmi1022* |  |  |  |  | Coiled coil |
|  |  |  |  | *lfa0244* |  |  | Coiled coil |
|  |  |  |  | *lfa0263* |  |  | Coiled coil |
|  |  |  |  | *lfa0332* |  |  | Coiled coil |
|  |  |  |  | *lfa1515* |  |  | Coiled coil |
|  |  |  |  | *lfa1534* |  |  | Coiled coil |
|  |  |  |  | *lfa2081* |  |  | Coiled coil |
|  |  |  |  | *lfa3500* |  |  | Coiled coil |
|  |  |  |  | *lfa3778* |  |  | Coiled coil |
|  |  |  | *lha1446* |  |  |  | Coiled coil |
|  |  |  | *lha1573* |  |  |  | Coiled coil |
|  |  |  | *lha2960* |  |  |  | Coiled coil |
|  |  |  | *lha3213* |  |  |  | Coiled coil |
|  |  |  |  | *lfa1983* |  |  | Coiled-coil |
| *lpg0171** |  |  |  |  |  |  | F-box |
| *lpg1408#* | *llo1494* |  | *lha0088* | *lfa1337#* |  |  | F-box |
| *lpg2224** |  |  |  |  | *ppgA* |  | F-box |
| *lpg2525** |  |  |  |  |  |  | F-box |
|  | *llo1427* | *lmi2723* |  |  |  |  | LRR + putative F-box |
|  | *llo2109* |  |  |  |  |  | F-Box |
|  |  | *lmi1926* |  |  |  |  | putative F-box |
|  |  | *lmi2472* |  |  |  |  | putative F-box |
|  |  |  |  | *lfa0504* |  |  | putative F-box |
|  |  |  |  | *lfa1843* |  |  | F-box |
|  |  | *lmi1175* |  | *lfa3280* |  |  | putative F-box |
| *lpg0945** |  |  |  |  |  |  | putative F-box |
| *lpg1602** |  | *lmi1113* |  |  |  |  | LRR |
| *lpg1660** |  |  |  |  |  |  | LRR |
| *lpg1890** |  |  |  | *lfa2611#* |  |  | LRR |
| *lpg1948** |  |  |  |  |  |  | LRR |
| *lpg1958** |  |  | *lha3152* |  |  |  | LRR |
| *lpg2392** |  |  | *lha0122* |  |  |  | LRR |
|  | *llo0114* |  |  |  |  |  | LRR |
|  | *llo1314* |  |  |  |  |  | LRR |
|  | *llo1643* |  |  | *lfa0081* |  |  | LRR |
|  | *llo2165* |  |  |  |  |  | LRR |
|  | *llo2494* |  |  |  |  |  | LRR |
|  | *llo3116* |  |  |  |  |  | LRR |
|  |  | *lmi0717* |  | *lfa0689* |  |  | LRR + putative F-box |
|  |  | *lmi1129* |  |  |  |  | LRR |
|  |  |  | *lha1823* | *lfa0468* |  |  | LRR |
|  |  |  |  | *lfa0530* |  |  | LRR |
|  |  |  |  | *lfa0545* |  |  | LRR |
|  |  |  |  | *lfa1451* |  |  | LRR |
|  |  |  |  | *lfa1881* |  |  | LRR |
|  |  |  |  | *lfa2158* |  |  | LRR |
|  |  |  |  | *lfa3760* | *atpI* |  | LRR |
|  | *llo2249* |  |  |  |  |  | Miro-like domains |
|  | *llo2424#* |  |  | *lfa1192* |  |  | MIRO |
|  |  | *lmi2158#* | *lha1310 #* | *lfa2000* |  |  | Miro domain |
|  | *llo2352* |  |  |  |  |  | PAM2 |
|  | *llo0793* |  |  |  |  |  | Phosphatidylinositol-4-phosphate 5-kinase |
|  | *llo1404* |  |  |  |  |  | PPR |
|  | *llo2643* |  |  |  |  |  | PPR, coiled-coil |
|  | *llo1892* |  |  |  |  |  | Putative Immunoglobulin I-set domain |
|  | *llo1716* |  |  |  |  |  | Ras-relatedsmall GTPase + Miro-like domain |
|  | *llo2329* |  |  |  |  |  | Ras-related small GTPase |
|  | *llo3288* |  |  | *lfa1897* |  |  | Ras-related small GTPase |
|  |  | *lmi0854* |  |  |  |  | Ras-related small GTPase |
|  |  |  |  | *lfa1711* |  |  | Ras-related small GTPase |
|  |  |  | *lha0998* |  |  |  | Ras-related small GTPase |
|  |  |  | *lha2690* |  |  |  | Ras-related small GTPase |
|  |  |  | *lha2693* |  |  |  | Ras-related small GTPase |
| *lpg0276** | *llo0327* |  |  | *lfa3277* |  |  | RASGEF |
|  | *llo0252* |  |  |  |  |  | RASGEF |
|  |  | *lmi1442* |  |  |  |  | RasGEF |
|  |  |  | *lha1465* |  |  |  | RASGEF |
|  |  |  | *lha2522* |  |  |  | RASGEF |
| *lpg1950** | *llo1397* |  |  |  | *ralF* |  | Sec-7 |
| *lpg0896* | *llo0844* | *lmi1282* | *lha2054* | *lfa0969* |  |  | SEL-1 |
| *lpg1062* |  |  |  |  |  |  | SEL-1 |
| *lpg1172* |  |  |  | *lfa1109* |  |  | SEL1 |
| *lpg1356* | *llo1443* | *lmi1775* | *lha1825* | *lfa1281* |  |  | SEL1 |
| *lpg2222** |  |  | *lha2129* | *lfa3092* |  |  | SEL-1 |
| *lpg2485* | *llo3057* | *lmi0649* | *lha0495* | *lfa3345* |  |  | SEL1 |
| *lpg2639* | *llo2649* | *lmi2478* | *lha0834* | *lfa2584* |  |  | SEL1 |
|  |  | *lmi1065* |  |  |  |  | Sel1 |
|  |  | *lmi3093* | *lha1431* |  |  |  | SET |
|  | *llo2327* |  |  |  |  |  | SH2 |
|  |  |  | *lha2583* |  |  |  | SH2 |
|  | *llo1196* |  |  |  |  |  | Snare |
|  | *llo2381* |  |  |  |  |  | Snare |
| *lpg1868* | *llo1280* | *lmi2333 #* | *lha1002 #* | *lfa2032#* | *yheS* |  | spectrine |
| *lpg1884** |  |  |  |  | *ylfB* |  | Spectrin |
| *lpg1947** |  |  |  |  |  |  | Spectrin |
| *lpg0208* |  |  |  |  |  |  | STPK |
| *lpg1483** | *llo1682* | *lmi1545* |  | *lfa1431* |  |  | STPK |
| *lpg2556*#* | *llo2218* |  |  |  |  |  | STPK |
|  | *llo1139* |  |  |  |  |  | STPK |
|  | *llo1681* |  |  |  |  |  | STPK |
|  | *llo1984* |  |  |  |  |  | STPK |
|  | *llo2984* |  |  |  |  |  | STPK |
|  | *llo3049* |  |  |  |  |  | STPK |
|  |  | *lmi3084* |  |  |  |  | STPK |
|  |  |  |  | *lfa0264* |  |  | STPK |
|  |  |  |  | *lfa0703* |  |  | STPK |
|  |  |  | *lha2947* | *lfa1579* |  |  | STPK |
|  |  |  | *lha2809* | *lfa1669* |  |  | STPK |
|  |  |  |  | *lfa2557* |  |  | STPK |
|  |  |  | *lha0212* |  |  |  | STPK |
|  |  |  | *lha0371* |  |  |  | STPK |
|  |  |  | *lha2268* |  |  |  | STPK |
|  | *llo2200* | *lmi0200* | *lha3048* | *lfa0156* |  |  | TTL |
| *lpg2830** |  |  |  |  | *lubX* | *legU2* | U-box |
|  | *llo0448* |  |  |  |  |  | U-Box |
|  |  | *lmi2124* |  |  |  |  | U-box |
|  |  |  | *lha0244* |  |  |  | U-box |

*Gene encoding a protein confirmed to be secreted by the Dot/Icm secretion system.

**#** Orthologous protein in which the corresponding EM is absent

ANK= ankyrin repeat; STPK= serine-threonine protein kinases; LRR= Leucine reach repeat; PPR= Pentatricopeptide repeat TTL=tubuline-tyrosine ligase
